# Molecular Genetics of Pre-B Acute Lymphoblastic Leukemia Sister Cell Lines during Disease Progression

**DOI:** 10.3390/cimb43030149

**Published:** 2021-11-30

**Authors:** Hilmar Quentmeier, Claudia Pommerenke, Hans G. Drexler

**Affiliations:** Department of Human and Animal Cell Lines, Leibniz-Institute DSMZ—German Collection of Microorganisms and Cell Cultures, 38124 Braunschweig, Germany; claudia.pommerenke@dsmz.de (C.P.); Hans.Drexler101@t-online.de (H.G.D.)

**Keywords:** *MEF2D-BCL9*, *PAX5* R38H, pre-B-ALL

## Abstract

For many years, immortalized tumor cell lines have been used as reliable tools to understand the function of oncogenes and tumor suppressor genes. Today, we know that tumors can comprise subclones with common and with subclone-specific genetic alterations. We sequenced DNA and RNA of sequential sister cell lines obtained from patients with pre-B acute lymphoblastic leukemia at different phases of the disease. All five pairs of cell lines carry alterations that are typical for this disease: loss of tumor suppressors (*CDKN2A*, *CDKN2B*), expression of fusion genes (*ETV6-RUNX1*, *BCR-ABL1*, *MEF2D-BCL9*) or of genes targeted by point mutations (*KRAS* A146T, *NRAS* G12C, *PAX5* R38H). *MEF2D-BCL9* and *PAX* R38H mutations in cell lines have hitherto been undescribed, suggesting that YCUB-4 (*MEF2D-BCL9*), PC-53 (*PAX* R38H) and their sister cell lines will be useful models to elucidate the function of these genes. All aberrations mentioned above occur in both sister cell lines, demonstrating that the sisters derive from a common ancestor. However, we also found mutations that are specific for one sister cell line only, pointing to individual subclones of the primary tumor as originating cells. Our data show that sequential sister cell lines can be used to study the clonal development of tumors and to elucidate the function of common and clone-specific mutations.

## 1. Introduction

For many decades, cell lines have enabled the modeling of human disease in cell culture for a better understanding of a plethora of pathophysiological processes and also, most importantly, for drug screening. Clearly, cell lines represent a useful in vitro tool owing to their easy manipulability and reduced culture costs; furthermore, they constitute a renewable resource, and, provided they are grown under recommended conditions, they retain their characteristic features and the results are reproducible. Nevertheless, a detailed and fully annotated characterization is fundamental before their use. In fact, in many cancer research fields, cell lines offer the most comprehensively characterized platform [1,2].

Additionally, in the domain of hematopoietic tumors, cell lines represent vital and powerful models in experimental systems to unravel the pro-leukemogenic roles of specific genetic mutations. As all leukemia cell lines inevitably carry molecular alterations, it is also essential here for the utility of these models to appropriately detail these genomic changes [3].

Despite the achievement of a complete remission in the vast majority of patients with acute lymphoblastic leukemia (ALL), ALL relapse remains a leading cause of childhood cancer-related death [4]. Relapsed ALL is thought to always be clonally related to the disease at diagnosis. Prior studies suggest that clonal mutations at relapse emerge from relapse-favoring subclones that already existed at diagnosis [4]. Their actual molecular and cellular input to therapy resistance and relapse remain, however, incompletely understood. The field of clonal evolution is an intensely discussed topic, both generally in cancer but also specifically in leukemia [5,6,7].

To obtain further insight into the relative impact of founding genomic alterations and acquired genetic alterations, we set out to examine the gene mutation status and gene expression of paired cell lines procured from ALL patients at diagnosis and at subsequent relapse or at other consecutive stages of their disease, aiming at a better understanding of the ALL relapse processes.

## 2. Material and Methods

### 2.1. Cell Lines

Cell lines NALM-20 and NALM-21 were taken from the stock of the cell lines bank (Leibniz Institut DSMZ—Deutsche Sammlung von Mikroorganismen und Zellkulturen GmbH, Braunschweig, Germany). All other cell lines were supplied for research purposes. Cell lines were authenticated by DNA profiling. Detailed references and cultivation protocols for NALM-20, NALM-27 and PC-53 have been described previously [8]. 

### 2.2. RNA-Sequencing Analysis

Total RNA was extracted via the miRNeasy Mini Kit (Qiagen, Hilden, Germany) including DNase digestion. Library preparation and sequencing steps were commissioned to GATC Biotech (Cologne, Germany). The GATC pipeline included the production of strand-specific (fr-first strand) mRNA libraries, quality control via Applied Biosystems Fragment Analyzer and Nanodrop, and concentration measurement via Qubit fluorometer. The libraries were sequenced on Illumina HiSeq2500 (2 × 151 cycles, paired end run, 8 bp dual indices) with >29 million reads per sample and deposited at ArrayExpress (E-MTAB-11038). Reads were trimmed via fastq-mcf (ea-utils 1.04.807), quality-controlled via FastQC (www.bioinformatics.babraham.ac.uk/projects/fastqc, accessed on 28 May 2019 and aligned by STAR (2.5.3a) [9] to the Gencode Homo sapiens genome (v26) and converted/sorted via samtools (0.1.19) [10]. Counting the reads to each gene was done via HTSeq-count python script (0.8.0) [11]. Data was processed and analyzed in the R/Bioconductor environment (3.3.2/3.3, www.bioconductor.org. Normalization, estimation of dispersions, and testing for differentially expressed genes based on a test assuming a negative binomial data distribution were computed via DESeq2 [12].

Fusion genes were detected by fusionCatcher (0.99.6a beta) (≥1 min-split-read + ≥3 min-span-pairs; hg38) [13]. 

### 2.3. Whole Exome Sequencing (WES) Analysis

DNA was isolated with the High Pure PCR Template Preparation Kit (Roche Diagnostics, Mannheim, Germany). Library preparation (Agilent SureSelect Human All Exon V6, 60 MB) and sequencing steps (2 × 151 bp + 8 bp barcoding, HiSeqX) were commissioned to Genewiz (Leipzig, Germany) and deposited at ArrayExpress (E-MTAB-11039). Insert lengths were aimed to be higher than 250 bp in order to increase the coverage and uniformity in coding regions [14].

Reads were aligned by STAR (2.5.3a) [9] to the human gencode genome (v26). Subsequently, alignment files were processed (samtools 0.1.19), duplicates removed (picard 2.9.2, www.broadinstitute.github.io/picard/, accessed on 28 May 2019), and variants called via GATK tools (3.7, Haplotypecaller) [15] and overlapping VarScan (v2.4.3) [16] results. Mutation effects were annotated via Ensembl VEP (release-84, GRCh38) [17]. Data were processed and analyzed in the R/Bioconductor environment (3.3.2/3.3). Overlapping single nucleotide variations via Haplotypecaller and VarScan were filtered for ≥20 quality, ≥10 depth, ≥0.2 allele frequency, <0.01 MAF, and missense/frameshift/stop gained mutations. 

### 2.4. RT-PCR, Genomic PCR and Sanger Sequencing

cDNA was prepared using the SuperScript II reverse transcriptase kit (Invitrogen, Karlsruhe, Germany). PCR was performed for 36 cycles on a C1000 Thermal Cycler (Bio-Rad, Dreieich, Germany) with an annealing temperature of 59 °C. The PCR primers are listed in Supplementary Table S3 (See Supplementary Materials). After agarose gel electrophoresis, the PCR products were purified with the QIAquick gel extraction kit (Qiagen, Hilden, Germany) and Sanger-sequenced (Eurofins, Ebersberg, Germany). 

### 2.5. Numerical Aberrations

A CytoScan HD Array (Affymetrix, Santa Clara, CA, USA) hybridization analysis was performed to identify numerical aberrations. DNA was prepared using the Qiagen Gentra Puregene Kit (Qiagen, Hilden, Germany). Data were analyzed using the Chromosome Analysis Suite software version 2.0.1.2 (Affymetrix). 

## 3. Results

### 3.1. Mutations and Chromosomal Aberrations

Encoding *ETV6-RUNX1*, t(12;21)(p13;q22) is the prevalent chromosomal rearrangement in pediatric B-ALL [18], detectable in 15–25% of cases [19]. We had found that 0/13 adult pre-B ALL cell lines and 1/13 pediatric pre-B ALL cell lines (REH) expressed the *ETV6-RUNX1* fusion, previously known as *TEL-AML1* [20].

**Figure 1:**
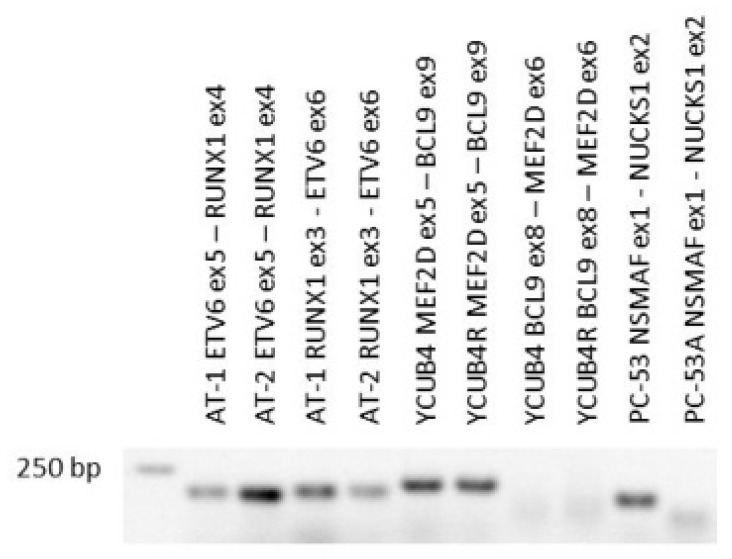
Transcripts of fusion genes in pre-B ALL cell lines. *ETV6-RUNX1*, *RUNX1-ETV6*, *MEF2D-BCL9*, *BCL9-MEF2D* and *NSMAF-NUCKS1* RT-PCR products are shown, separated on an agarose gel.

The RNA-seq data analysis shows that AT-1 and sister cell line AT-2, obtained from a 5 year old boy with pre-B ALL at 1st and 2nd relapse [21], also express the *ETV6* exon 5/*RUNX1* exon 4 fusion (Table 1, Figure 1, Supplementary Figure S1A). The cell lines were also published as SUP-B26/SUP-B28. The reverse *RUNX1* exon 3/*ETV6* exon 6 fusion transcript is also present in both cell lines (Table 1, Figure 1, Supplementary Figure S1B). *ETV6-RUNX1* is commonly detectable at birth. Secondary events are obligatory to induce tumorigenesis [19]. *ETV6* is a partner of gene fusions but is also recurrently deleted in pre-B ALL [22]. Indeed, the *ETV6-RUNX1* positive cell lines AT-1 and AT-2 had lost one *ETV6* allele (Table 1). Thus, they express the fusion but not the wild-type form of *ETV6*.

t(9;22) fusing *BCR* and *ABL1* is the hallmark of chronic myelogenous leukemia (CML), but it also occurs in 3–5% of childhood B-ALL and in 25% of adult ALL cases [19]. NALM-20 and NALM-21 express the *BCR-ABL1* fusion transcript (Table 1) [23]. NALM-20 was raised from a 62-year-old patient at diagnosis and NALM-21 from the same patient at relapse. NALM-27 and NALM-30 are also *BCR-ABL1*-positive (Table 1) [24]. NALM-27 was raised from a 38 year old patient at diagnosis, and the sister cell line NALM-30 was derived at relapse [25].

**Figure 2:**
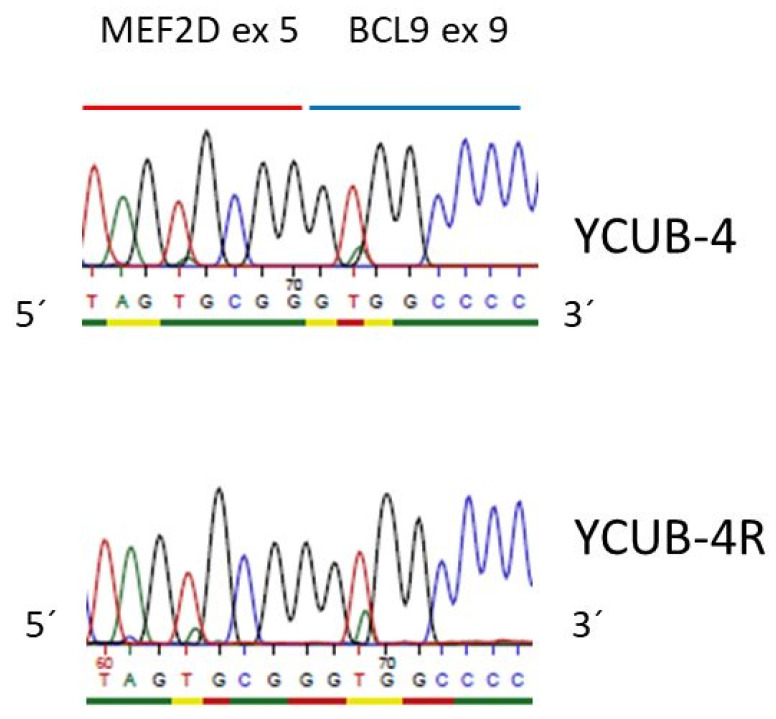
*MEF2D/BCL9* fusion mRNA. Pre-B ALL cell lines YCUB-4 and YCUB-4R express the *MEF2D* exon 5/*BCL9* exon 9 fusion transcript. The MEF2D/BCL9 PCR product was sequenced with the *MEF2D* exon 5 forward primer (Supplementary Table S3). The fusion transcript is in frame.

In 2016, *MEF2D* translocations were described in pre B-ALL, with *BCL9* being the most common fusion partner of *MEF2D* [26]. YCUB-4 and the sister cell line YCUB-4R express the *MEF2D* exon 5/BCL9 exon 9 fusion transcript, resulting from t(1;1)(q21.2;q22) (Table 1, Figure 1 and Figure 2). These cell lines are from a 7 year old boy with pre-B ALL at diagnosis and relapse [27]. The *MEF2D-BCL9* fusion is not the only pre-B ALL characteristic genetic alteration in this pair of cell lines. YCUB-4 and YCUB-4R also show a hemizygous loss of *PAX5* (Table 1). *PAX5* deletions had been found in 56/192 B-progenitor ALL cases [28].

**Figure 3:**
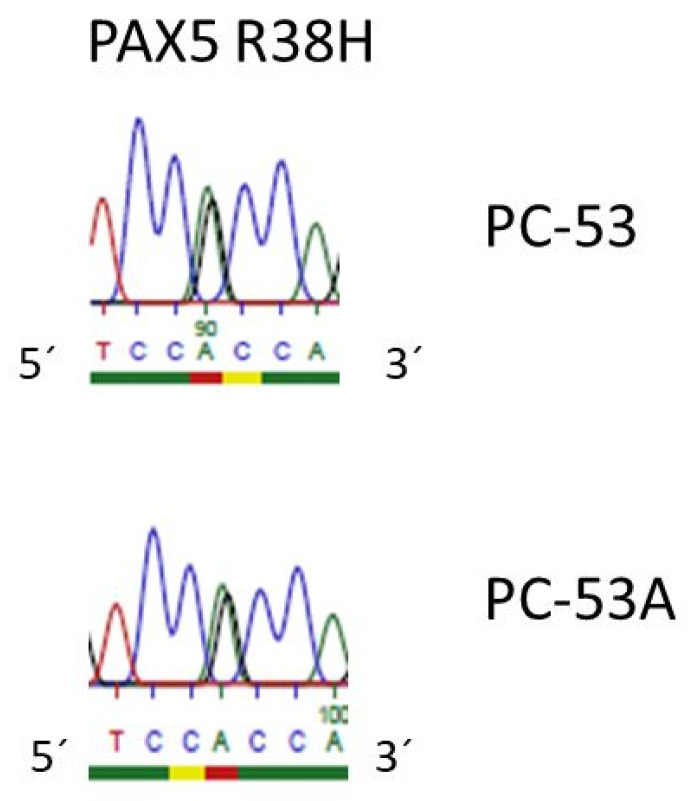
*PAX5* R38H mutation in PC-53 and PC-53A. Genomic PCR and sequencing show that the pre-B ALL cell lines PC-53 and PC-53 carry the *PAX5* R38H (cGc/cAc) mutation (0/1). Sequencing was performed with the *PAX5* intron 1/2 forward primer (Supplementary Table S3).

*PAX5* is also a partner of translocations, and it is the target of point mutations in this disease [28,29]. PC-53, from a 33 year old woman with pre-B ALL at 3rd relapse and the sister cell line PC-53A (at the final, refractory stage) [30], carry the *PAX5* R38H mutation (Table 1, Figure 3). This mutation had been described in the context of pre-B ALL (COSM5986423). Additional mutations specifically occurring in one of the sister cell lines are shown in Supplementary Table S1 (exemplarily for chromosome 14). As assessed by a principal component analysis (PCA), the sister cell lines show closely related gene expression profiles (Supplementary Figure S2).

Focal deletions or sequence mutations of *IKZF1* are recurrent in pediatric ALL [19]. *IKZF1* can be lost as whole or can be subject to partial deletions [31]. NALM-20 and NALM-27, which were derived from two patients, show partial deletions of *IKZF1* (0n) along with their sister cell lines (Table 1, Supplementary Figure S3). Partial *IKZF1* deletions and deletions of *CDKN2A* and *CDKN2B* are markers of disease recurrence in adolescent and adult Philadelphia chromosome-negative pre-B ALL [32]. In pediatric ALL, two-thirds of *BCR-ABL1*-positive cases and a lower proportion (<5–25% depending on the subtype) of *BCL-ABL1*-negative cases carry the *IKZF1* deletion [33]. Noticeably, both pairs of cell lines with an *IKZF1* deletion are from adults and carry the *BCR-ABL1* fusion (Table 1). All five pairs of sister cell lines show deletions of *CDKN2A* and *CDKN2B* (Table 1, Supplementary Figure S4).

*BTG1*, *BTLA*, *NR3C1* and *TP53* are other genes that are recurrently deleted in pre-B ALL [19,32,34]. One allele of these genes is lost in at least one pair of our five sister cell line sets (Table 1).

None of the aberrations listed so far affected only one of the sister cell lines exclusively. Obviously, none of the mutations had developed during tumor progression. Therefore, the mutations described so far did not allow for a description of subclonal development. However, we found other mutations that indeed indicate that such a process did occur.

### 3.2. Subclonal Developments in Sister Cell Lines

The *NSMAF* (ex 1)—*NUCKS1* (ex 2) fusion is expressed in the cell line PC-53 but not in the sister cell line PC-53A (Table 1, Figure 1, Supplementary Figure S5). This fusion has not been reported in the context of pre-B ALL so far. However, the presence of *NSMAF-NUCKS1* in the earlier cell line and absence in the later suggests that *NSMAF-NUCKS1*-positive and -negative clones already existed when cell line PC-53 was raised. The results of the comparative genomic hybridization (CGH) analysis showed that in PC-53 both genes, *NSMAF* and *NUCKS1*, were located in the middle of the transition between two copy numbers (Supplementary Figure S6). These data localize the *NSMAF* and *NUCKS1* translocation breakpoints in cell line PC-53 at the appropriate chromosomal positions. PC-53A does not show these differences in copy numbers (Supplementary Figure S6). Thus, the sister cell lines exhibited molecular differences, providing an explanation for the exclusive expression of the *NSMAF-NUCKS1* (t(1;8)(q32.1;q12.1)) fusion transcript in PC-53 and not in PC-53A.

Because the CGH analysis was suitable for detecting molecular differences between the sister cell lines PC-53 and PC-53A, we performed copy number analyses to elucidate whether other sister cell lines carried clone-specific copy number differences as well. Most aberrations detected by the CGH analysis were found in both of the sister cell lines (Supplementary Table S2; highlighted in green). In 4/5 sister pairs, we found aberrations in the later sister that were not present in the early sister (Supplementary Table S2; highlighted in red). However, we also detected aberrations in the earlier cell line that did not exist in the later cell line (Supplementary Table S2; highlighted in purple). 

The WES data analysis indicated that the sister cell lines AT-1 and AT-2 consisted of subclones with *KRAS* A146T (Gca/Aca) or with *NRAS* G12C (Ggt/Tgt) mutations (Table 1). Both sister cell lines are diploid for these genes (Supplementary Figure S7). Therefore, one would expect 50% reads for the wild-type and mutant versions of the genes, respectively. In AT-2, 50% of reads of *KRAS* were wild type and 50% were mutant (*KRAS* A146T; Gca/Aca) (Table 1, Supplementary Figure S8). However, only 15% of the reads were mutant in cell line AT-1 (Table 1, Supplementary Figure S8). Furthermore, the reads for the *NRAS* mutation did not follow the expected 50% wild-type and 50% mutant scheme. In AT-1, 35% of reads encoding *NRAS* G12C (Ggt/Tgt) were mutant. In AT-2, 3% were mutant (Table 1, Supplementary Figure S9). 

The easiest explanation for these results is that both sister cell lines comprise two subclones, “clone A” being *KRAS* homozygously wild-type (0/0)/*NRAS* heterozygously mutant (0/1) and “clone B” being *KRAS* (0/1)/*NRAS* (0/0). Table 2 shows the proportion of these subclones in the two sister cell lines based on read numbers and under the assumption that the subclones carry wild-type and mutant versions of the genes.

## 4. Discussion

We have previously shown that B-lymphoma cell lines can comprise subclones [35]. Twelve percent of cell lines with immunoglobulin (IG) hypermutations (6/49) consisted of subclones with individual IG mutations [35]. The cell line U-2932 was analyzed in depth. The cell line consists of two subclones with genomic and subgenomic aberrations including common (*BCL2*) and clone-specific (*MYC*) alterations [36]. The differential expression of over 60 genes in the two subclones could be traced back to genomic copy number variations or consequences of the differential expression of the transcription factor *BCL6* [37]. The immunoglobulin hypermutation patterns of both subclones were identified in the DNA from the primary material of the patient, confirming that the two clones of the cell line truly represented subclones of the tumor [36]. 

Immunoglobulin hypermutations occur in the dark zone of the germinal center. In the current study, we studied pre-germinal center B cells. Therefore, a hypermutation analysis could not be applied to identify subclones. Instead, we performed numerical analyses and next generation sequencing analyses. We found disease-characteristic aberrations that were common to both sister cell lines as well as mutations in one of the sisters only. Aberrations that affected both sisters included losses of *CDKN2A*, *CDKN2B*, *ETV6*, *IKZF1* and *PAX5*, all of them recurrent deletions in pre-B ALL (Table 1) [19,28,29,31,32]. *BCR-ABL1*, *ETV6-RUNX1* and *MEF2D-BCL9* are fusions that also occur recurrently in pre-B ALL [19,26]. At least one of the five pairs of cell lines expressed the corresponding fusion transcripts (Table 1). *MEF2D* (ex 5)—*BCL9* (ex 9) in YCUB-4 and YCUB-4R was especially noteworthy. *BCL9* is the most frequent fusion partner of *MEF2D* in pre-B ALL [26]. To our knowledge, this is the first description of a cell line expressing a *MEF2D-BCL9* fusion transcript. What is also novel is the *PAX5* R38H mutation (COSM5986423) in cell lines PC-53 and PC-53A (Table 1). *PAX5* mutations including point mutations are recurrent in B-cell ALL [28], and a cell line carrying such a mutation might help to elucidate the pathogenetic sequelae of the mutation for the cell. 

All point mutations, deletions and fusion transcripts described so far always affected both sister cell lines. When one sister carried a mutation, the other did so as well. Apparently, these mutations, characteristic for the disease, already existed in the originating tumor cell when the earlier sister cell line was established. None of them allowed one to distinguish between early and late sister cell lines. 

The situation was different when we looked at copy number aberrations in toto, i.e., not specifically at deletions known to contribute to the disease. Then, additionally, most deletions and amplifications occurred in both sisters (Supplementary Table S2). However, 4/5 pairs of cell lines showed aberrations in one of the sisters only (Supplementary Table S2). Interestingly, earlier sister cell lines also carried specific aberrations (Supplementary Table S2). The expression of the *NSMAF-NUCKS1* fusion transcript in PC-53 was specific for the earlier sister (Table 1). Abnormalities in the chromosomal regions of *NSMAF* and *NUCKS1* in PC-53, but not in PC-53A, confirmed that the differential expression of the fusion transcript was caused at the molecular level (Supplementary Figure S6). This observation, as well as the observation that sister cell lines exhibit unique copy number alterations not shared by the other sister, suggest that the sister cell lines represent tumor clones that independently developed from an ancestor already carrying the mutations that are common to both sisters (Supplementary Table S2; the common alterations are highlighted in green).

Without DNA from the primary tumor, we could not formally exclude that the sister cell specific aberrations had developed in vitro. However, the WES analysis of *KRAS* and *NRAS* mutations in AT-1 and AT-2 suggests that both serial sister cell lines consist of two clones from the primary tumor. Both mutations, *KRAS* A146T (Gca/Aca; COSM19404) and *NRAS* G12C (Ggt/Tgt; COSM561), were detected in each of the sister cell lines, albeit at different percentages and not with the 50% wt vs. 50% mu read proportion that would be expected if a cell clone carried a wild-type and mutant version of a gene (Table 1, Supplementary Figures S8 and S9 ). It is highly unlikely that two identical mutations have occurred independently in vitro in two cell lines from one patient. Therefore, our data favor the view that two clones, one with the *KRAS* A146T and the other with the *NRAS* G12C mutation, had already existed in the patient. AT-1 and AT-2, established at first and second relapse, comprise both subclones, albeit at different percentages (Table 2).

In sum, the results of the RNA-seq, WES and CGH analyses of five pairs of sequential pre-B ALL cell lines show aberrations characteristic for the disease. Because they are unreported in cell lines so far, the *MEF2D-BCL9* fusion in YCUB-4 and YCUB-4R and the *PAX5* R38H mutation in PC-53 and PC-53A are noteworthy. Mutations specifically occurring in one of the sisters suggest a derivation from individual clones of the primary tumor, whereas common mutations point to the common ancestor. The study shows that sequential sister cell lines allow one to study common and clone-specific mutations.

## Figures and Tables

**Table 1:** Mutations and genomic aberrations in pre-B ALL sister cell lines.

Copy Number	AT-1 (5y)	AT-2 (5y)	NALM-20 (62y)	NALM-21 (63y)	NALM-27 (38y)	NALM-30 (39y)	PC-53 (33y)	PC-53A (34y)	YCUB-4 (7y)	YCUB-4R (7y)
*BTG1* (12q21.33)	2n	2n	**1n**	**1n**	2n	2n	2n	2n	2n	2n
*BTLA* (3q13.2)	2n	2n	**1n**	**1n**	**1n**	**1n**	2n	2n	2n	2n
*CDKN2A* (9p21.3)	**0n**	**0n**	**0n**	**0n**	**0n**	**0n**	**0n**	**0n**	**0n**	**0n**
*CDKN2B* (9p21.3)	**0n**	**0n**	**0n (partial)**	**0n (partial)**	**0n (partial)**	**0n (partial)**	**0n**	**0n**	**0n**	**0n**
*ETV6* (12p13.2)	**1n**	**1n**	2n	2n	2n	2n	2n	2n	2n	2n
*IKZF1* (7p12.2)	2n	2n	**0n (partial)**	**0n (partial)**	**0n (partial)**	**0n (partial)**	2n	2n	2n	2n
*NR3C1* (5q31.3)	2n	2n	2n	2n	2n	2n	**1n**	**1n**	2n	2n
*PAX5* (9p13.2)	2n	2n	2n	2n	2n	1.5–2n	2n	2n	**1n**	**1n**
*TP53* (17p13.1)	2n	2n	2n	2n	2n	2n	**1n**	**1n**	2n	2n
**fusion transcripts**										
*BCR-ABL1*	no	no	**yes**	**yes**	**yes**	**yes**	no	no	no	no
*ETV6-RUNX1*	**yes**	**yes**	no	no	no	no	no	no	no	no
*MEF2D-BCL9*	no	no	no	no	no	no	no	no	**yes**	**yes**
*NSMAF-NUCKS1*	no	no	no	no	no	no	**yes**	**no**	no	no
*RUNX1-ETV6*	**yes**	**yes**	no	no	no	no	no	no	no	no
**point mutations**										
*KRAS* A146T COSM19404	**0/1 (85/15)**	**0/1 (50/50)**	no	no	no	no	no	no	no	no
*NRAS* G12C COSM561	**0/1 (65/35)**	**0/1/ (97/3)**	no	no	no	no	no	no	no	no
*PAX5* R38H COSM5986423	no	no	no	no	no	no	**0/1**	**0/1**	no	no

Aberrations and differences between sister cell lines are highlighted. Fusion transcripts were detected by analyzing RNAseq data with fusion_catcher; all fusion transcripts are in frame. Numeric status by CGH analysis, point mutations by WES analysis. COSM numbers are legacy mutation identifiers identifying existing mutations: 0/1: wild type/mutant; 0/0: wild type/wild type; numbers in brackets (wild type reads/mutant reads).

**Table 2:** The mutational analyses of *KRAS* and *NRAS* suggest that AT-1 and AT-2 are comprised of subclones.

Cell Line	Clone A *KRAS* (0/0)/*NRAS* (0/1)	Clone B *KRAS* (0/1)/*NRAS* (0/0)
AT-1	70%	30%
AT-2	<5%	>95%

Mutations were detected by WES analysis. (0/0) homozygously wild-type; (0/1) wild-type and mutant.

## Data Availability

RNAseq (E-MTAB-11038) and WES data (E-MTAB-11039) were data were deposited at ArrayExpress.

## Supplementary Materials

The following are available online at https://www.mdpi.com/article/10.3390/cimb43030149/s1, Figure S1: Fusion transcripts joining *ETV6* and *RUNX1*, Figure S2: Principal component analysis plot, Figure S3: *IKZF1* deletions, Figure S4: *CDKN2A*/*CDKN2B* deletions, Figure S5: NSMAF/NUCKS1 fusion mRNA, Figure S6: Chromosomal aberrations affecting *NSMAF* and *NUCKS1*, Figure S7: *KRAS* and *NRAS* diploid in AT-1 and AT-2, Figure S8: *KRAS* A146T (Gca/Aca) mutation in AT-1 and AT-2, Figure S9: *NRAS* G12C (Gct/Tgt) mutation in AT-1 and AT-2, Table S1: Mutations and genomic aberrations of chromosome 14 in pre-B ALL sister cell lines, Table S2: Numerical aberrations according to CGH analysis, Table S3: Sequences of PCR primers.
