# Supplementary material for: Molecular Genetics of Pre-B Acute Lymphoblastic Leukemia Sister Cell Lines during Disease Progression
Source: Curr Issues Mol Biol. 2021 Nov 30;43(3):2147–56. doi: 10.3390/cimb43030149 (PMC8929001; doi:10.3390/cimb43030149)
Supplement: Supplementary file 1 [file cimb-43-00149-s001.zip › cimb-1467938 supplementary-Tables S1-S3.pdf]

Supplementary Table S1. Mutations and genomic aberrations of chromosome 14 in pre-B ALL sister cell lines .

id	SYMBOL	Existing_vari	GMAF	Consequenc	cDNA_positi	Protein_posi	Amino_acids	Codons	SIFT	PolyPhen	AT-1	AT-2	NALM-20	NALM-21	NALM-27	NALM-30	PC-53	PC-53A	YCUB-4	YCUB-4R
chr14_100097	EVL	rs200233370		missense_var	581.00	120.00	G/S	Ggc/Agc	tolerated(0.05	probably_damaging(0.998)										
chr14_100149	DEGS2	rs4905937	T:0.1424	missense_var	745.00	57.00	A/T	Gcc/Acc	tolerated(0.37	possibly_damaging(0.449)										
chr14_100159	DEGS2	rs7157599	C:0.2328	missense_var	599.00	8.00	S/N	aGc/aAc	tolerated(0.46	benign(0)	0/1	0/1	1/1	1/1	0/1	0/1	1/1	1/1	1/1	1/1
chr14_100328	SLC25A47	rs35007880	T:0.3273	missense_var	482.00	135.00	R/L	cGg/cTg	tolerated(0.17	possibly_damaging(0.541)					0/1	0/1	1/1	1/1		
chr14_100381	WDR25	rs2181170	T:0.0014	missense_var	488.00	88.00	W/R	Tgg/Cgg	tolerated_low_	benign(0)	1/1	1/1	1/1	1/1	1/1	1/1	1/1	1/1	1/1	1/1
chr14_100381	WDR25	rs2273800	A:0.3530	missense_var	672.00	149.00	H/R	cAt/cGt	tolerated(0.41	benign(0)	0/1	0/1	0/1	0/1	0/1	0/1	1/1	1/1		
chr14_100732	DLK1	rs6575799	A:0.0032	missense_var	543.00	101.00	R/G	Agg/Ggg	tolerated(1)	benign(0)	1/1	1/1					1/1			
chr14_100732	DLK1	rs2273607	A:0.0124	missense_var	552.00	104.00	V/M	Gtg/Atg	tolerated(0.18	benign(0.056)									0/1	0/1
chr14_100734	DLK1	rs1058009	A:0.0555	missense_var	1021.00	260.00	S/N	aGc/aAc	tolerated(0.28	benign(0.003)	0/1	0/1								
chr14_100881	RTL1	rs61993318	T:0.0022	missense_var	3703.00	1215.00	R/H	cGt/cAt	tolerated_low_	benign(0.124)										
chr14_100882	RTL1	rs112608318	T:0.0102	missense_var	2613.00	852.00	A/T	Gcc/Acc	deleterious(0)	probably_dam	0/1	0/1								
chr14_100882	RTL1	rs11623267,C	G:0.2009	missense_var	2601.00	848.00	E/Q	Gag/Cag	tolerated(0.08	possibly_dam	0/1	0/1	0/1	0/1	0/1	0/1				
chr14_100883	RTL1	rs41286560,C	T:0.0078	missense_var	1731.00	558.00	P/T	Cca/Aca	tolerated(0.75	benign(0.003)										
chr14_100883	RTL1	rs200271756		missense_var	1273.00	405.00	N/S	aAt/aGt	tolerated(0.14	benign(0.003)							0/1	0/1		
chr14_100883	RTL1	rs142029300	T:0.0022	missense_var	1254.00	399.00	E/K	Gaa/Aaa	tolerated(0.13	benign(0.147)										
chr14_100884	RTL1	rs149778073	A:0.0046	missense_var	211.00	51.00	P/L	cCa/cTa	tolerated_low_	benign(0.003)			0/1	0/1						
chr14_101983	DYNC1H1			stop_gained	1160.00	332.00	Y/*	taC/taG									0/1			
chr14_102102	HSP90AA1	rs8005905	T:0.1989	missense_var	493.00	71.00	M/L	Atg/Ttg	deleterious_lo	benign(0)	1/1	1/1	1/1	1/1	1/1	1/1	1/1	1/1	1/1	1/1
chr14_102229	MOK	rs2236493	C:0.2993	missense_var	1425.00	398.00	Q/R	cAg/cGg	tolerated(0.18	benign(0)			0/1	0/1	1/1				0/1	0/1
chr14_102326	ZNF839	rs7158731	C:0.4185	missense_var	368.00	118.00	L/P	cTc/cCc	tolerated(1)	benign(0)										
chr14_102326	ZNF839	rs7158139	A:0.4181	missense_var	613.00	200.00	D/N	Gat/Aat	tolerated(1)	benign(0)										
chr14_102348	CINP	rs7011	T:0.1649	missense_var	532.00	164.00	R/H	cGc/cAc	tolerated(0.18	benign(0.003)							1/1	1/1		
chr14_102428	TECPR2	rs1309353	G:0.1396	missense_var	1184.00	320.00	V/I	Gtt/Att	tolerated(1)	benign(0)	1/1		1/1	1/1	1/1	1/1				0/1
chr14_102432	TECPR2	rs2273906	T:0.0429	missense_var	1541.00	439.00	P/S	Ccc/Tcc	tolerated(0.64	benign(0.007)	0/1	0/1								
chr14_102434	TECPR2	rs10149146	G:0.2149	missense_var	2273.00	683.00	I/V	Atc/Gtc	tolerated_low_	benign(0)	0/1	0/1			0/1	0/1	1/1	1/1		
chr14_102452	TECPR2	rs75004775	A:0.0056	missense_var	3691.00	1155.00	S/R	agC/agA	deleterious(0.	benign(0.066)					0/1	0/1				
chr14_102507	ANKRD9	rs750170448		missense_var	788.00	64.00	R/H	cGc/cAc	tolerated(0.14	benign(0.284)					0/1	0/1				
chr14_102507	ANKRD9			missense_var	727.00	44.00	L/M	Ctg/Atg	tolerated(0.06	benign(0.3)									0/1	0/1
chr14_102593	RCOR1			missense_var	494.00	90.00	G/R	Ggc/Cgc	deleterious(0.	probably_damaging(0.98)										0/1
chr14_102875	TRAF3	rs1131877	C:0.4698	missense_var	603.00	129.00	M/T	aTg/aCg	tolerated(0.67	benign(0)	0/1	0/1							0/1	0/1
chr14_103100	EXOC3L4	rs2297067	T:0.2133	missense_var	305.00	77.00	R/W	Cgg/Tgg	deleterious(0)	possibly_dam	0/1	0/1	0/1	0/1	0/1	0/1				
chr14_103100	EXOC3L4	rs2297066	G:0.2422	missense_var	355.00	93.00	D/E	gaC/gaG	tolerated(1)	benign(0)	0/1	0/1	0/1	0/1	0/1	0/1				
chr14_103102	EXOC3L4	rs10131298	A:0.2650	missense_var	630.00	185.00	L/H	cTt/cAt	tolerated(0.08	benign(0.01)	0/1	0/1	0/1	0/1	0/1	0/1				
chr14_103104	EXOC3L4	rs117708804	T:0.0917	missense_var	1202.00	376.00	A/S	Gcc/Tcc	tolerated(0.13	benign(0.055)			0/1	0/1	0/1	0/1				
chr14_103110	EXOC3L4	rs729184	A:0.2288	missense_var	2100.00	675.00	Q/R	cAg/cGg	tolerated(1)	benign(0)	1/1	1/1	1/1	1/1	0/1	0/1	1/1	1/1		
chr14_103110	EXOC3L4	rs744153	C:0.4361	missense_var	2129.00	685.00	Q/E	Caa/Gaa	deleterious(0.	benign(0.207)	1/1	1/1			0/1	0/1	0/1	0/1	1/1	1/1
chr14_103127	TNFAIP2	rs1132339	C:0.4774	missense_var	1483.00	282.00	Q/E	Cag/Gag	deleterious(0)	possibly_damaging(0.571)			0/1	0/1	1/1	1/1	0/1	0/1		
chr14_103563	APOPT1	rs2274268	G:0.2346	missense_var	80.00	27.00	P/A	Cct/Gct	tolerated(0.22	benign(0)										
chr14_103563	APOPT1	rs200407081	A:0.0020	missense_var	144.00	48.00	R/K	aGg/aAg	tolerated(0.24	benign(0.084)					0/1	0/1				
chr14_103699	XRCC3	rs861539,CM	A:0.2169	missense_var	1519.00	241.00	T/M	aCg/aTg	tolerated(0.17	benign(0.19)										
chr14_103915	C14orf2	rs1053419	C:0.1833	missense_var	326.00	26.00	I/V	Ata/Gta	tolerated(1)	benign(0)			0/1	0/1			0/1			
chr14_103940	RD3L	rs35337422	C:0.0525	missense_var	721.00	166.00	I/R	aTa/aGa	deleterious(0)	probably_damaging(1)										
chr14_103941	RD3L	rs17101950	T:0.0306	missense_var	354.00	44.00	L/I	Tta/Ata	tolerated(0.05	possibly_damaging(0.795)			0/1	0/1			0/1	0/1	0/1	0/1
chr14_104093	ASPG	rs1770984	C:0.4127	missense_var	375.00	95.00	C/R	Tgc/Cgc	tolerated(1)	benign(0)	0/1	0/1	1/1	1/1					0/1	0/1
chr14_104093	ASPG	rs1744284	G:0.4125	missense_var	378.00	96.00	L/V	Ctt/Gtt	tolerated(0.13	benign(0)	0/1	0/1	1/1	1/1					0/1	0/1
chr14_104104	ASPG	rs8012505	G:0.1044	missense_var	1124.00	344.00	S/R	agC/agG	deleterious(0.	probably_damaging(0.917)					0/1	0/1			0/1	0/1
chr14_104176	KIF26A	rs3742946	A:0.0196	missense_var	3319.00	1107.00	G/S	Ggc/Agc	tolerated(0.51	benign(0)	0/1	0/1								
chr14_104176	KIF26A	rs117573425	T:0.0254	missense_var	3364.00	1122.00	A/S	Gcc/Tcc	tolerated(0.65	benign(0.001)										
chr14_104709	INF2	rs138577569,	T:0.0020	missense_var	2090.00	660.00	R/W	Cgg/Tgg	deleterious(0.	possibly_damaging(0.877)									0/1	0/1
chr14_104714	INF2	rs3803311	T:0.0090	missense_var	3516.00	1135.00	T/M	aCg/aTg	tolerated_low_	benign(0.288)			0/1	0/1						
chr14_104801	ZBTB42	rs4983387	G:0.4107	missense_var	979.00	232.00	E/K	Gaa/Aaa	tolerated(0.08	possibly_damaging(0.552)			1/1	1/1	1/1	1/1	1/1	1/1	0/1	0/1
chr14_104802	ZBTB42			missense_var	1355.00	357.00	S/L	tCg/tTg	deleterious(0)	probably_damaging(0.999)					0/1	0/1				
chr14_104884	CEP170B	rs773965520,COSM118892		missense_var	1634.00	469.00	R/Q	cGg/cAg	deleterious(0)	probably_damaging(1)							0/1	0/1		
chr14_104885	CEP170B	rs375753389		missense_var	2098.00	624.00	V/M	Gtg/Atg	tolerated(0.2)	benign(0.02)	0/1									
chr14_104927	PLD4	rs2841280	C:0.4119	missense_var	292.00	34.00	E/Q	Gag/Cag	tolerated_low_	benign(0.017)	1/1	1/1	0/1	0/1	0/1	0/1			1/1	1/1
chr14_104927	PLD4			stop_gained	369.00	59.00	W/*	tgG/tgA											0/1	0/1
chr14_104932	PLD4	rs74316182	A:0.0254	missense_var	1315.00	375.00	G/S	Ggc/Agc	tolerated(0.51	benign(0.003)					0/1	0/1	0/1	0/1		
chr14_104939	AHNAK2	rs3742935	G:0.4449	missense_var	16309.00	5397.00	P/A	Cct/Gct	deleterious(0.	possibly_damaging(0.672)			0/1	0/1	0/1	0/1	1/1	1/1		

chr14_104939	AHNAK2	rs2819419	A:0.4091	missense_var	15670.00	5184.00	Y/D	Tac/Gac	tolerated(0.67	benign(0)			0/1	0/1	0/1	0/1	1/1	1/1		
chr14_104940	AHNAK2	rs61421370	T:0.2931	missense_var	15536.00	5139.00	G/E	gGg/gAg	tolerated(0.65	benign(0.062)							0/1	0/1		
chr14_104940	AHNAK2	rs77215854	A:0.0254	missense_var	14581.00	4821.00	P/S	Cct/Tct	tolerated(0.93	benign(0.015)					0/1	0/1	0/1	0/1		
chr14_104941	AHNAK2	rs76611205	A:0.0254	missense_var	14383.00	4755.00	P/S	Cca/Tca	tolerated(0.15	benign(0.031)					0/1	0/1	0/1	0/1		
chr14_104941	AHNAK2	rs4465542	T:0.4449	missense_var	14110.00	4664.00	T/A	Aca/Gca	tolerated(0.99	benign(0)			0/1	0/1	0/1	0/1	1/1	1/1		
chr14_104941	AHNAK2	rs9672139	T:0.4724	missense_var	13726.00	4536.00	M/L	Atg/Ctg	tolerated(1)	benign(0.022)			0/1	0/1			0/1	0/1		
chr14_104942	AHNAK2	rs2819421	A:0.4449	missense_var	13097.00	4326.00	L/P	cTg/cCg	tolerated(1)	benign(0)				0/1	0/1	0/1		1/1		
chr14_104942	AHNAK2	rs11850949	A:0.4692	missense_var	13081.00	4321.00	L/V	Ttg/Gtg	tolerated(1)	benign(0.327)			0/1	0/1				1/1		
chr14_104942	AHNAK2	rs2819422	A:0.4193	missense_var	12953.00	4278.00	V/A	gTg/gCg	tolerated(0.45	benign(0.006)			0/1	0/1	0/1	0/1	1/1	1/1		
chr14_104943	AHNAK2	rs10141053	C:0.2458	missense_var	12001.00	3961.00	M/V	Atg/Gtg	tolerated(1)	benign(0.001)							0/1	0/1		
chr14_104943	AHNAK2	rs10438246	T:0.4441	missense_var	11725.00	3869.00	M/V	Atg/Gtg	tolerated(1)	benign(0)			0/1	0/1	0/1	0/1	1/1	1/1		
chr14_104944	AHNAK2	rs11160825	C:0.4503	missense_var	11497.00	3793.00	D/N	Gac/Aac	tolerated(0.1)	benign(0.125)			0/1	0/1	0/1	0/1	1/1	1/1		
chr14_104944	AHNAK2	rs28380382	C:0.4475	missense_var	11081.00	3654.00	G/E	gGg/gAg	tolerated(0.06	benign(0.158)			0/1	0/1	0/1	0/1	1/1	1/1		
chr14_104945	AHNAK2	rs376878100		missense_var	10546.00	3476.00	P/S	Ccc/Tcc	deleterious(0.	probably_damaging(0.995)							0/1	0/1		
chr14_104945	AHNAK2	rs4264326,rs	A:0.4365	missense_var	10208.00	3363.00	V/A	gTg/gCg	tolerated(1)	benign(0)			0/1	0/1	0/1	0/1	1/1	1/1		
chr14_104945	AHNAK2	rs10438247	G:0.4615	missense_var	10127.00	3336.00	P/L	cCg/cTg	deleterious(0)	probably_damaging(0.909)			0/1	0/1	0/1	0/1	1/1	1/1		
chr14_104945	AHNAK2	rs28714612	G:0.3852	missense_var	9899.00	3260.00	M/T	aTg/aCg	tolerated(0.21	benign(0)							0/1	0/1		
chr14_104945	AHNAK2	rs77154428	A:0.0014	missense_var	9842.00	3241.00	R/H	cGc/cAc	deleterious(0)	benign(0.048)							0/1	0/1		
chr14_104945	AHNAK2	rs2819426	C:0.4329	missense_var	9649.00	3177.00	L/V	Ctg/Gtg	tolerated(1)	benign(0.005)			0/1	0/1	0/1	0/1	1/1	1/1		
chr14_104945	AHNAK2	rs11160826	T:0.0108	missense_var	9648.00	3176.00	D/E	gaC/gaG	tolerated(0.68	benign(0.183)			0/1	0/1	0/1	0/1	1/1	1/1		
chr14_104946	AHNAK2	rs12433837	C:0.4814	missense_var	9367.00	3083.00	V/I	Gtc/Atc	tolerated(1)	benign(0.116)			0/1	0/1	0/1	0/1	1/1	1/1		
chr14_104946	AHNAK2	rs3000771		missense_var	9347.00	3076.00	R/H	cGc/cAc	deleterious(0)	benign(0.033)			0/1	0/1	0/1	0/1	1/1	1/1		
chr14_104946	AHNAK2	rs2582514	G:0.4329	missense_var	8704.00	2862.00	R/S	Cgc/Agc	tolerated(1)	benign(0)			0/1	0/1	0/1	0/1	1/1	1/1		
chr14_104947	AHNAK2	rs57308399	A:0.0110	missense_var	8117.00	2666.00	S/F	tCc/tTc	deleterious(0)	probably_damaging(0.988)					0/1	0/1				
chr14_104947	AHNAK2	rs60754080	A:0.4802	missense_var	7670.00	2517.00	G/V	gGg/gTg	tolerated(1)	benign(0)					0/1		1/1	1/1		
chr14_104947	AHNAK2	rs2819429	T:0.1567	missense_var	7628.00	2503.00	E/A	gAg/gCg	deleterious(0.	benign(0.168)	1/1	1/1	0/1	0/1	1/1	1/1	1/1	1/1	0/1	0/1
chr14_104948	AHNAK2	rs72702027	G:0.4681	missense_var	7279.00	2387.00	P/S	Cca/Tca	tolerated(0.07	possibly_damaging(0.708)			0/1	0/1	0/1	0/1	1/1	1/1		
chr14_104948	AHNAK2	rs2582513	A:0.4369	missense_var	7118.00	2333.00	L/P	cTt/cCt	tolerated(1)	benign(0)			0/1	0/1	0/1	0/1	1/1	1/1		
chr14_104949	AHNAK2	rs141736470	C:0.1238	missense_var	6562.00	2148.00	T/A	Acc/Gcc	tolerated(1)	benign(0.009)	1/1	1/1								
chr14_104949	AHNAK2	rs12890949	T:0.1492	missense_var	6556.00	2146.00	L/V	Ctg/Gtg	tolerated(1)	benign(0.001)	1/1	1/1	0/1	0/1	1/1	1/1	1/1	1/1	1/1	1/1
chr14_104949	AHNAK2	rs199550173	C:0.0621	missense_var	6547.00	2143.00	N/D	Aac/Gac	tolerated(1)	benign(0)	1/1	1/1								
chr14_104949	AHNAK2	rs200601510	A:0.0775	missense_var	6538.00	2140.00	T/S	Acc/Tcc	tolerated(1)	benign(0)	1/1	1/1								
chr14_104949	AHNAK2	rs11846918	C:0.4481	missense_var	6439.00	2107.00	M/V	Atg/Gtg	tolerated(1)	benign(0)			0/1	0/1	0/1	0/1	1/1	1/1		
chr14_104949	AHNAK2	rs11850848	C:0.3630	missense_var	6300.00	2060.00	H/Q	caC/caG	tolerated(1)	benign(0.029)			0/1	0/1	0/1	0/1	1/1	1/1		
chr14_104949	AHNAK2	rs117379881	G:0.2772	missense_var	6163.00	2015.00	A/P	Gca/Cca	tolerated(0.31	probably_damaging(0.953)							0/1	0/1		
chr14_104949	AHNAK2	rs118171013	A:0.3003	missense_var	6160.00	2014.00	P/S	Cct/Tct	tolerated(1)	benign(0)							0/1	0/1		
chr14_104949	AHNAK2	rs59117977	T:0.1322	missense_var	5821.00	1901.00	G/S	Ggc/Agc	tolerated(0.28	benign(0.013)			0/1	0/1						
chr14_104949	AHNAK2	rs2819435,C	T:0.2029	missense_var	5688.00	1856.00	E/D	gaA/gaT	tolerated(1)	benign(0.001)			0/1	0/1	1/1	1/1	1/1	1/1	0/1	0/1
chr14_104949	AHNAK2	rs11851005	G:0.1122	missense_var	5678.00	1853.00	V/A	gTg/gCg	tolerated(0.38	benign(0.019)			0/1	0/1	0/1	0/1			0/1	0/1
chr14_104949	AHNAK2	rs144488514	G:0.0455	missense_var	5624.00	1835.00	V/A	gTg/gCg	tolerated(0.29	benign(0.187)					0/1	0/1			0/1	0/1
chr14_104950	AHNAK2	rs61428154	G:0.0519	missense_var	5233.00	1705.00	D/H	Gac/Cac	tolerated(0.12	probably_damaging(0.991)					0/1	0/1			0/1	0/1
chr14_104950	AHNAK2	rs34752012	G:0.0220	missense_var	5174.00	1685.00	E/A	gAg/gCg	tolerated(0.77	benign(0)							0/1	0/1		
chr14_104950	AHNAK2	rs2013462	A:0.4311	missense_var	4949.00	1610.00	V/A	gTg/gCg	tolerated(1)	benign(0)					0/1	0/1	1/1	1/1		
chr14_104950	AHNAK2	rs77454674,C	G:0.0298	missense_var	4937.00	1606.00	K/T	aAa/aCa	deleterious(0.	probably_damaging(0.976)				0/1						
chr14_104950	AHNAK2	rs187739436,	C:0.0026	missense_var	4872.00	1584.00	F/L	ttC/ttG	tolerated(0.54	benign(0.005)			0/1	0/1						
chr14_104950	AHNAK2	rs61996045	A:0.2816	missense_var	4805.00	1562.00	P/L	cCa/cTa	tolerated(0.11	possibly_damaging(0.66)							0/1	0/1		
chr14_104950	AHNAK2	rs148890906	A:0.0104	missense_var	4708.00	1530.00	P/S	Ccc/Tcc	tolerated(0.06	possibly_damaging(0.871)					0/1	0/1				
chr14_104951	AHNAK2	rs144323040	G:0.0361	missense_var	4512.00	1464.00	E/D	gaG/gaC	deleterious(0.	benign(0.037)					0/1	0/1	1/1	1/1		
chr14_104951	AHNAK2	rs141283979	T:0.0218	missense_var	4327.00	1403.00	E/K	Gag/Aag	tolerated(0.35	possibly_damaging(0.634)							1/1	1/1		
chr14_104951	AHNAK2	rs2396457,C	A:0.3145	missense_var	4142.00	1341.00	S/F	tCt/tTt	deleterious(0)	probably_damaging(0.988)			0/1	0/1	0/1	0/1	1/1	1/1		
chr14_104951	AHNAK2	rs2819440,C	C:0.2165	missense_var	4014.00	1298.00	M/I	atG/atC	tolerated(0.68	benign(0)	0/1	0/1	0/1	0/1	1/1	1/1	1/1	1/1	0/1	0/1
chr14_104951	AHNAK2	rs55650155	A:0.3830	missense_var	3644.00	1175.00	A/V	gCg/gTg	tolerated(1)	benign(0)			0/1	0/1			0/1	0/1		
chr14_104952	AHNAK2	rs55791176	G:0.0018	missense_var	3564.00	1148.00	E/D	gaA/gaC	tolerated(1)	benign(0)			0/1	0/1	0/1	0/1	1/1	1/1		
chr14_104952	AHNAK2	rs11625007		missense_var	3517.00	1133.00	V/I	Gtc/Atc	tolerated(1)	benign(0.006)			0/1	0/1	0/1	0/1	1/1	1/1		
chr14_104952	AHNAK2	rs201268922	T:0.1284	missense_var	3406.00	1096.00	L/I	Ctc/Atc	tolerated(0.74	benign(0.062)				0/1	0/1		0/1			
chr14_104952	AHNAK2	rs151013711	T:0.2039	missense_var	3404.00	1095.00	A/D	gCc/gAc	tolerated(1)	benign(0)				0/1	0/1	0/1	0/1	0/1		
chr14_104952	AHNAK2	rs74090129	C:0.2067	missense_var	2674.00	852.00	M/V	Atg/Gtg	tolerated(0.71	benign(0.015)			0/1	0/1	0/1	0/1	0/1	0/1		
chr14_104953	AHNAK2	rs201524595	G:0.0142	missense_var	2357.00	746.00	G/A	gGc/gCc	tolerated(0.23	possibly_damaging(0.712)							0/1	0/1		
chr14_104953	AHNAK2	rs78204285	A:0.2027	missense_var	2351.00	744.00	P/L	cCg/cTg	tolerated(0.07	benign(0.38)			0/1	0/1			0/1	0/1		
chr14_104953	AHNAK2	rs45448397,C	T:0.0080	missense_var	1774.00	552.00	D/N	Gat/Aat	tolerated(0.52	benign(0.031)							0/1	0/1		

chr14_104953	AHNAK2	rs2278607	C:0.2019	missense_var	1693.00	525.00	T/A	Act/Gct	tolerated(1)	benign(0.013)			0/1	0/1	0/1	0/1	0/1	0/1		
chr14_105149	JAG2	rs9972231	T:0.1200	missense_var	2016.00	538.00	D/N	Gac/Aac	tolerated(0.12	benign(0.025)			0/1	0/1	0/1	0/1			0/1	0/1
chr14_105150	JAG2	rs1057744	C:0.4798	missense_var	1905.00	501.00	E/K	Gag/Aag	tolerated(0.2)	benign(0)							1/1	1/1	0/1	0/1
chr14_105391	PACS2	TMP_ESP_14_105858022_		missense_var	2349.00	725.00	P/L	cCg/cTg	deleterious(0.	possibly_damaging(0.511)							0/1	0/1		
chr14_105464	MTA1	rs4983413	A:0.2849	missense_var	1328.00	372.00	V/I	Gtc/Atc	tolerated(0.2)	benign(0.067)	1/1	1/1	0/1	0/1	0/1	0/1			1/1	1/1
chr14_105469	MTA1	rs13707	G:0.1458	missense_var	2048.00	612.00	A/T	Gcc/Acc	tolerated(0.89	benign(0)	1/1	1/1	1/1	1/1	1/1	1/1	1/1	1/1	1/1	1/1
chr14_105479	CRIP2	rs782276346		missense_var	992.00	236.00	P/S	Ccc/Tcc	tolerated(0.75	possibly_damaging(0.877)			0/1	0/1						
chr14_105479	CRIP2	rs782586339		missense_var	1115.00	277.00	E/K	Gaa/Aaa	deleterious_lc	benign(0.214)	0/1	0/1								
chr14_105488	CRIP1	rs55633823	T:0.0779	missense_var	1116.00	58.00	A/V	gCa/gTa	tolerated(0.06	benign(0.102)							1/1	1/1		
chr14_105497	C14orf80	rs80142487	T:0.0142	missense_var	818.00	231.00	R/W	Cgg/Tgg	tolerated(0.14	possibly_damaging(0.629)					0/1	0/1			0/1	0/1
chr14_105498	C14orf80	rs782021817,COSM136870		missense_var	1116.00	330.00	A/V	gCg/gTg	tolerated(0.34	benign(0)	0/1	0/1								
chr14_106359	IGHV3-33			missense_var	430.00	117.00	R/K	aGa/aAa	tolerated_low	benign(0.047)									0/1	
chr14_106359	IGHV3-33			stop_gained	293.00	71.00	W/*	tgG/tgA				1/1								
chr14_106359	IGHV3-33			missense_var	292.00	71.00	W/S	tGg/tCg	tolerated_low	benign(0)		1/1								
chr14_106762	AC245369.3			missense_var	335.00	93.00	E/K	Gaa/Aaa	tolerated(0.57	benign(0)										
chr14_106770	IGHV2-70	rs17113973	C:0.0046	missense_var	232.00	78.00	K/E	Aaa/Gaa	deleterious(0.	benign(0.172)										
chr14_106770	IGHV2-70	rs2073669	A:0.4615	missense_var	212.00	71.00	L/R	cTc/cGc	tolerated(0.36	benign(0.001)	0/1	1/1								
chr14_186013	OR11H12			missense_var	274.00	68.00	W/R	Tgg/Cgg	tolerated(0.52	benign(0)	1/1						0/1			
chr14_186013	OR11H12	rs61969156		missense_var	338.00	89.00	V/A	gTc/gCc	deleterious(0.	benign(0.335)	0/1	0/1								
chr14_186013	OR11H12	rs202115314		missense_var	341.00	90.00	S/F	tCt/tTt	deleterious(0.	probably_damaging(0.945)								0/1		
chr14_186014	OR11H12	rs138502172		missense_var	360.00	96.00	M/I	atG/atA	deleterious(0.	possibly_dam	0/1	0/1								
chr14_186014	OR11H12	rs2815978		missense_var	386.00	105.00	K/T	aAa/aCa	deleterious(0.	possibly_dam	0/1	0/1			0/1	0/1		0/1		
chr14_186015	OR11H12	rs138051664		missense_var	509.00	146.00	L/H	cTc/cAc	tolerated(1)	benign(0)	0/1	0/1	0/1	0/1	0/1	0/1	0/1	0/1	0/1	
chr14_186017	OR11H12			missense_var	668.00	199.00	R/L	cGa/cTa	tolerated(1)	benign(0)	0/1	0/1	0/1	0/1	0/1	0/1	0/1	0/1	0/1	0/1
chr14_186018	OR11H12	rs61969158		missense_var	791.00	240.00	V/G	gTg/gGg	deleterious(0)	possibly_dam	0/1	0/1	0/1	0/1			0/1	0/1		
chr14_186019	OR11H12	rs201551285		missense_var	874.00	268.00	S/G	Agc/Ggc	tolerated(1)	benign(0)	0/1	0/1			0/1	0/1	0/1	0/1		
chr14_189675	POTEM	rs201262647	A:0.2171	missense_var	72.00	7.00	S/L	tCa/tTa	deleterious_lc	possibly_damaging(0.629)			0/1	0/1	0/1		0/1	0/1		
chr14_189774	POTEM			missense_var	975.00	308.00	V/A	gTt/gCt	tolerated(1)	benign(0)									0/1	0/1
chr14_189854	POTEM	rs781261134		missense_var	1188.00	379.00	L/S	tTa/tCa	deleterious(0.	possibly_dam	0/1	0/1					0/1	0/1		
chr14_194134	POTEG	rs199722113,	G:0.1436	missense_var	1353.00	434.00	P/R	cCt/cGt	deleterious(0.	benign(0.014)			0/1	0/1					0/1	0/1
chr14_194134	POTEG			missense_var	1352.00	434.00	P/T	Cct/Act	tolerated(1)	benign(0)									0/1	0/1
chr14_194163	POTEG	rs200944601		missense_var	1188.00	379.00	L/S	tTa/tCa	tolerated(0.05	possibly_dam	0/1	0/1								
chr14_194287	POTEG			missense_var	702.00	217.00	R/Q	cGg/cAg	tolerated(1)	benign(0)									0/1	
chr14_194338	POTEG	rs79870143		missense_var	488.00	146.00	A/T	Gct/Act	deleterious(0)	probably_dam	0/1	0/1	0/1	0/1		0/1			0/1	0/1
chr14_194339	POTEG	rs76596948		missense_var	431.00	127.00	A/T	Gct/Act	deleterious(0.	probably_damaging(0.964)								0/1		
chr14_194340	POTEG			missense_var	278.00	76.00	S/G	Agc/Ggc	tolerated_low	benign(0)	0/1	0/1			0/1	0/1	0/1			
chr14_194342	POTEG	rs28406802	T:0.2516	missense_var	72.00	7.00	S/L	tCa/tTa	deleterious_lc	possibly_damaging(0.835)		0/1	0/1				0/1	0/1		
chr14_197131	OR11H2			missense_var	877.00	269.00	P/S	Cct/Tct	tolerated(1)	benign(0)	0/1		1/1	1/1	0/1	0/1				
chr14_197131	OR11H2			missense_var	874.00	268.00	S/G	Agc/Ggc	tolerated(1)	benign(0)						0/1				
chr14_197133	OR11H2	rs201024081		missense_var	646.00	192.00	V/I	Gtt/Att	tolerated(0.08	benign(0.017)	0/1	0/1			0/1	0/1	0/1	0/1	0/1	0/1
chr14_197134	OR11H2			missense_var	539.00	156.00	Y/C	tAt/tGt	tolerated(1)	benign(0)	0/1						0/1	0/1		
chr14_197136	OR11H2	rs200825460		missense_var	386.00	105.00	K/T	aAa/aCa	deleterious(0.	probably_damaging(0.932)							0/1	0/1		
chr14_197137	OR11H2	rs759418864		missense_var	260.00	63.00	V/A	gTc/gCc	tolerated(1)	benign(0)								0/1		
chr14_197137	OR11H2			missense_var	218.00	49.00	I/T	aTa/aCa	tolerated(0.76	benign(0)					0/1	0/1				0/1
chr14_197478	OR4Q3	rs17210864	G:0.1096	missense_var	526.00	135.00	T/A	Aca/Gca	tolerated(0.16	benign(0.005)										
chr14_197479	OR4Q3	rs148505982	T:0.0114	stop_gained	601.00	160.00	Q/*	Cag/Tag			0/1	0/1								
chr14_197481	OR4Q3	rs12896533	T:0.2901	missense_var	835.00	238.00	F/L	Ttc/Ctc	tolerated(1)	benign(0)	0/1	0/1	1/1	1/1	1/1	1/1	0/1	0/1	1/1	1/1
chr14_197806	OR4M1	rs2635535	T:0.2544	missense_var	466.00	116.00	T/I	aCa/aTa	tolerated(0.18	benign(0.012)	0/1	0/1			0/1	0/1	0/1	0/1	0/1	0/1
chr14_197807	OR4M1	rs200594443,COSM470995		stop_gained	501.00	128.00	R/*	Cga/Tga					0/1	0/1						
chr14_197810	OR4M1	rs2815960		missense_var	814.00	232.00	G/D	gGt/gAt	deleterious(0)	benign(0.003)	0/1	0/1			0/1	0/1	0/1	0/1	0/1	0/1
chr14_198274	OR4N2	rs72663752	G:0.0727	missense_var	546.00	7.00	T/R	aCa/aGa	deleterious(0)	possibly_dam	0/1	0/1								
chr14_198276	OR4N2	rs11621884	G:0.2075	missense_var	698.00	58.00	P/A	Ccc/Gcc	deleterious(0)	probably_damaging(1)							0/1	0/1	0/1	0/1
chr14_198276	OR4N2	rs2801164		missense_var	753.00	76.00	I/T	aTt/aCt	tolerated(0.1)	benign(0.018)			1/1	1/1	0/1	0/1	0/1	0/1	0/1	0/1
chr14_198278	OR4N2	rs2318279		missense_var	923.00	133.00	P/S	Cct/Tct	tolerated(0.95	benign(0)							0/1	0/1		0/1
chr14_198278	OR4N2	rs17114261		missense_var	929.00	135.00	V/L	Gtc/Ctc	tolerated(0.23	benign(0.007)							0/1	0/1		0/1
chr14_198281	OR4N2	rs117025898	A:0.0421	missense_var	1197.00	224.00	R/H	cGc/cAc	tolerated(0.65	benign(0.001)			0/1	0/1						
chr14_198771	OR4K2	rs12883767	T:0.2790	missense_var	1936.00	307.00	N/I	aAt/aTt	tolerated(0.06	benign(0.14)							0/1			
chr14_199214	OR4K5	rs77615110,C	A:0.1390	missense_var	866.00	281.00	V/I	Gtc/Atc	tolerated(0.7)	benign(0)			0/1	0/1	0/1	0/1			0/1	0/1
chr14_199215	OR4K5	rs17242341	A:0.1386	missense_var	981.00	319.00	R/K	aGa/aAa	tolerated_low	benign(0.091)			0/1	0/1	0/1	0/1			0/1	0/1
chr14_199359	OR4K1	rs12885778	A:0.3219	missense_var	533.00	89.00	R/H	cGc/cAc	tolerated(1)	benign(0)									0/1	0/1

chr14_19936	OR4K1	rs3916626	A:0.1575	missense_var	680.00	138.00	R/Q	cGg/cAg	tolerated(0.41	benign(0)			0/1	0/1	0/1	0/1			0/1	0/1
chr14_19936	OR4K1	rs34608158	A:0.1769	missense_var	1056.00	263.00	S/R	agC/agA	deleterious(0.	benign(0.111)									0/1	0/1
chr14_19936	OR4K1	rs34394400	T:0.2344	missense_var	1177.00	304.00	R/C	Cgt/Tgt	deleterious(0.	benign(0.359)									0/1	0/1
chr14_19936	OR4K1	rs2792146	A:0.4295	missense_var	1178.00	304.00	R/H	cGt/cAt	tolerated(0.2)	benign(0)	0/1	0/1	0/1	0/1	0/1	0/1	0/1	0/1	0/1	0/1
chr14_19975	OR4K15	rs4060024	G:0.3754	missense_var	341.00	89.00	N/S	aAc/aGc	deleterious(0.	possibly_dam	0/1	0/1	0/1	0/1	0/1	0/1				
chr14_19975	OR4K15	rs3861512	T:0.2183	missense_var	410.00	112.00	E/V	gAg/gTg	deleterious(0.	benign(0.273)	0/1	0/1	0/1	0/1	0/1	0/1				
chr14_19975	OR4K15	rs3861513	G:0.2183	missense_var	424.00	117.00	S/A	Tct/Gct	deleterious(0)	possibly_dam	0/1	0/1	0/1	0/1	0/1	0/1				
chr14_19976	OR4K15	rs10135246	A:0.2157	missense_var	839.00	255.00	A/E	gCa/gAa	tolerated(0.21	benign(0.093)	0/1	0/1	0/1	0/1	0/1	0/1				
chr14_19976	OR4K15	rs2153466	C:0.3764	missense_var	986.00	304.00	L/P	cTt/cCt	tolerated(1)	benign(0)	0/1	0/1	0/1	0/1	0/1	0/1				
chr14_19976	OR4K15	rs10135467	G:0.2194	missense_var	1005.00	310.00	I/M	atC/atG	deleterious(0.	probably_dam	0/1	0/1	0/1	0/1	0/1	0/1				
chr14_20002	OR4Q2	rs12587697	A:0.3141	missense_var	416.00	139.00	R/Q	cGg/cAg	tolerated(0.14	benign(0.021)			0/1	0/1	0/1	0/1			0/1	0/1
chr14_20002	OR4Q2	rs12586792	C:0.2901	missense_var	578.00	193.00	F/S	tTt/tCt	tolerated(0.14	possibly_damaging(0.892)			0/1	0/1	0/1	0/1			0/1	0/1
chr14_20014	OR4K14	rs17308108	C:0.0929	missense_var	628.00	145.00	L/R	cTg/cGg	deleterious(0)	probably_damaging(0.973)			0/1	0/1						
chr14_20034	OR4K13	rs76336343	A:0.0048	missense_var	936.00	106.00	L/F	Ctc/Ttc	tolerated(0.78	benign(0.003)	0/1	0/1	0/1	0/1						
chr14_20060	OR4L1	rs1958715	G:0.4633	missense_var	4.00	2.00	D/N	Gat/Aat	tolerated(0.16	benign(0.005)	0/1	0/1	0/1	0/1	0/1	0/1	0/1	0/1	0/1	0/1
chr14_20060	OR4L1	rs1958716	A:0.4633	missense_var	118.00	40.00	M/V	Atg/Gtg	tolerated(0.26	benign(0)	0/1	0/1	0/1	0/1	0/1	0/1	0/1	0/1	0/1	0/1
chr14_20060	OR4L1	rs1959630	G:0.0288	missense_var	156.00	52.00	R/S	agG/agT	tolerated(0.12	benign(0)	1/1	1/1	1/1	1/1	1/1	1/1	1/1	1/1	1/1	1/1
chr14_20060	OR4L1	rs2775253	A:0.4263	missense_var	302.00	101.00	M/K	aTg/aAg	deleterious(0)	possibly_dam	0/1	0/1	0/1	0/1	0/1	0/1	0/1	0/1	0/1	0/1
chr14_20060	OR4L1	rs2775254	A:0.4261	missense_var	325.00	109.00	G/S	Ggt/Agt	deleterious(0)	possibly_dam	0/1	0/1	0/1	0/1	0/1	0/1	0/1	0/1	0/1	0/1
chr14_20117	OR4K17	rs8005245,C	C:0.4275	missense_var	477.00	159.00	K/N	aaG/aaC	deleterious(0)	probably_dam	0/1	0/1	1/1	1/1	0/1	0/1			1/1	1/1
chr14_20197	OR11G2	rs4981822,C	A:0.4427	missense_var	296.00	99.00	I/N	aTc/aAc	deleterious(0)	possibly_dam	0/1	0/1	0/1	0/1	0/1	0/1	0/1	0/1	0/1	0/1
chr14_20197	OR11G2	rs4981088	A:0.4499	missense_var	346.00	116.00	V/I	Gtc/Atc	tolerated(0.06	benign(0.164)	0/1	0/1	0/1	0/1	0/1	0/1	0/1	0/1	0/1	0/1
chr14_20223	OR11H6	rs10140652	A:0.0994	missense_var	20.00	7.00	S/Y	tCt/tAt	tolerated_low	benign(0.015)					0/1	0/1				
chr14_20223	OR11H6	rs9323693,C	G:0.0994	missense_var	94.00	32.00	L/V	Ctc/Gtc	deleterious(0)	benign(0.38)					0/1	0/1				
chr14_20224	OR11H6	rs12891553	C:0.3910	missense_var	320.00	107.00	I/T	aTt/aCt	tolerated(1)	benign(0)	0/1	0/1			0/1	0/1	0/1	0/1	0/1	0/1
chr14_20224	OR11H6	rs17211285	T:0.3894	missense_var	585.00	195.00	L/F	ttG/ttT	tolerated(1)	benign(0)	0/1	0/1			0/1	0/1	0/1	0/1	0/1	0/1
chr14_20224	OR11H6	rs17277221	C:0.3910	missense_var	706.00	236.00	Y/H	Tac/Cac	deleterious(0)	probably_dam	0/1	0/1			0/1	0/1	0/1	0/1	0/1	0/1
chr14_20224	OR11H6	rs61993884	C:0.0439	missense_var	727.00	243.00	V/L	Gtg/Ctg	deleterious(0)	possibly_damaging(0.817)										
chr14_20224	OR11H6	rs17277228	C:0.3910	missense_var	775.00	259.00	C/R	Tgt/Cgt	deleterious(0)	probably_dam	0/1	0/1			0/1	0/1	0/1	0/1	0/1	0/1
chr14_20229	OR11H7	rs4981844	T:0.4453	missense_var	508.00	170.00	G/C	Ggt/Tgt	deleterious(0.	possibly_dam	0/1	0/1			1/1	1/1	0/1	0/1	0/1	0/1
chr14_20230	OR11H7			missense_var	898.00	300.00	A/T	Gcc/Acc	deleterious(0.	possibly_damaging(0.446)			0/1	0/1						
chr14_20243	OR11H4	rs17277270	G:0.0691	missense_var	955.00	301.00	T/S	aCt/aGt	tolerated(1)	benign(0)										
chr14_20301	TTC5	rs3742945	T:0.2544	missense_var	197.00	47.00	Q/R	cAg/cGg	tolerated(0.55	benign(0)	1/1	1/1	0/1	0/1	0/1	0/1	0/1	0/1	0/1	0/1
chr14_20357	PARP2	rs74485011	C:0.0010	missense_var	1468.00	481.00	N/H	Aat/Cat	tolerated(0.17	benign(0.025)					0/1	0/1				
chr14_20368	TEP1	rs2104978	C:0.0345	missense_var	7726.00	2562.00	H/R	cAt/cGt	tolerated(0.1)	probably_damaging(0.977)										
chr14_20369	TEP1	rs938886	C:0.3393	missense_var	7499.00	2486.00	I/M	atC/atG	tolerated_low	benign(0)							0/1	0/1		
chr14_20371	TEP1	rs199992841,	A:0.0002	stop_gained	7131.00	2364.00	R/*	Cga/Tga					0/1	0/1						
chr14_20373	TEP1	rs1713449	T:0.3241	missense_var	6681.00	2214.00	V/I	Gtc/Atc	tolerated(0.13	benign(0.001)							0/1	0/1		
chr14_20378	TEP1	rs8022805	T:0.0308	missense_var	5356.00	1772.00	R/Q	cGg/cAg	deleterious(0.	probably_damaging(0.96)										
chr14_20381	TEP1	rs1713456	T:0.2392	missense_var	4444.00	1468.00	C/Y	tGc/tAc	tolerated(1)	benign(0)					0/1	0/1				
chr14_20382	TEP1	rs144008371	A:0.0006	missense_var	4197.00	1386.00	R/W	Cgg/Tgg	deleterious(0)	possibly_damaging(0.765)										
chr14_20383	TEP1	rs1760904	A:0.3087	missense_var	3624.00	1195.00	S/P	Tca/Cca	tolerated(1)	benign(0)			0/1	0/1	0/1	0/1	0/1	0/1		
chr14_20384	TEP1	rs2228041,C	T:0.0335	missense_var	3505.00	1155.00	R/Q	cGg/cAg	tolerated(1)	benign(0)										
chr14_20384	TEP1	rs1760903	G:0.3666	missense_var	3204.00	1055.00	R/C	Cgt/Tgt	tolerated(0.17	benign(0)			0/1	0/1	0/1	0/1	0/1	0/1		
chr14_20404	TEP1	rs1760898	T:0.3648	missense_var	962.00	307.00	N/K	aaC/aaA	tolerated(0.37	benign(0)					0/1	0/1	0/1	0/1		
chr14_20408	TEP1	rs1760897,C	G:0.3592	missense_var	387.00	116.00	S/P	Tct/Cct	tolerated_low	benign(0)					0/1	0/1	0/1	0/1		
chr14_20428	KLHL33	rs7145318	C:0.3546	missense_var	1769.00	516.00	A/T	Gct/Act	tolerated_low	benign(0)	0/1	0/1	1/1	1/1	1/1	1/1	0/1	0/1		
chr14_20429	KLHL33	rs1953225	C:0.2592	missense_var	1257.00	345.00	E/G	gAg/gGg	tolerated(0.12	benign(0.023)	0/1	0/1	1/1	1/1	1/1	1/1				
chr14_20456	APEX1	rs1130409,C	G:0.3756	missense_var	712.00	148.00	D/E	gaT/gaG	tolerated(1)	benign(0)	1/1	1/1	1/1	1/1	0/1	0/1	0/1	0/1	0/1	0/1
chr14_20472	PNP	rs1049564,C	A:0.2047	missense_var	297.00	51.00	G/S	Ggt/Agt	tolerated(0.98	benign(0)	0/1	0/1					0/1	0/1		
chr14_20510	RNASE10	rs79798350	G:0.0479	missense_var	110.00	31.00	T/A	Aca/Gca	tolerated_low	benign(0)										
chr14_20510	RNASE10	rs2067648	A:0.0479	missense_var	411.00	131.00	S/N	aGt/aAt	tolerated(1)	benign(0)										
chr14_20556	RNASE9	rs1243647	A:0.2470	missense_var	880.00	209.00	S/P	Tcg/Ccg	tolerated_low	benign(0)	0/1	0/1						1/1		
chr14_20556	RNASE9	rs12590446	A:0.4225	missense_var	713.00	153.00	F/S	tTt/tCt	tolerated(0.52	benign(0.001)	0/1	0/1	1/1	1/1	1/1	1/1	1/1	1/1	1/1	1/1
chr14_20640	OR6S1	rs17277522	C:0.4684	missense_var	710.00	237.00	R/H	cGt/cAt	deleterious(0.	possibly_dam	0/1	0/1	0/1	0/1					1/1	1/1
chr14_20641	OR6S1	rs11622969	T:0.4485	missense_var	466.00	156.00	V/I	Gtc/Atc	tolerated(0.05	benign(0.028)	0/1	0/1	0/1	0/1	1/1	1/1	1/1	1/1		
chr14_20641	OR6S1	rs11622794	A:0.4603	missense_var	125.00	42.00	T/I	aCa/aTa	tolerated(0.33	benign(0.015)	0/1	0/1	0/1	0/1	1/1	1/1	1/1	1/1		
chr14_20699	RNASE4	rs3748338	T:0.1276	missense_var	722.00	16.00	T/S	Acc/Tcc	tolerated(0.07	possibly_damaging(0.495)					0/1	0/1			0/1	0/1
chr14_20747	EDDM3A	rs34552133	T:0.1130	missense_var	311.00	62.00	G/C	Ggc/Tgc	deleterious(0.	possibly_damaging(0.907)							0/1	0/1	0/1	0/1
chr14_20770	EDDM3B	rs3827906	G:0.1512	missense_var	111.00	5.00	L/V	Cta/Gta	tolerated(0.13	benign(0.068)			0/1	0/1						

chr14_20781	RNASE6	rs1045922	A:0.3049	missense_var	559.00	89.00	R/Q	cGg/cAg	tolerated(1)	benign(0.003)			0/1	0/1	0/1	0/1			1/1	1/1
chr14_20892	RNASE3	rs2073342,CM	C:0.3594	missense_var	429.00	124.00	T/R	aCg/aGg	tolerated(1)	benign(0)	0/1	0/1	1/1	1/1	0/1	0/1	1/1	1/1	1/1	1/1
chr14_20999	SLC39A2	rs2234632	T:0.2308	missense_var	285.00	43.00	L/R	cTa/cGa	tolerated(1)	benign(0)	0/1	0/1	1/1	1/1	1/1	1/1	1/1	1/1	0/1	0/1
chr14_20999	SLC39A2	rs2234633	C:0.0060	missense_var	300.00	48.00	L/P	cTg/cCg	deleterious(0)	probably_damaging(0.993)							0/1	0/1		
chr14_21000	SLC39A2	rs2234636	C:0.3199	missense_var	500.00	115.00	F/L	Ttt/Ctt	tolerated(1)	benign(0)					0/1	0/1			0/1	0/1
chr14_21043	RNASE7	rs1263872	G:0.1737	missense_var	564.00	103.00	A/P	Gcc/Ccc	tolerated(1)	benign(0)	1/1	1/1	0/1	0/1	1/1	1/1	1/1	1/1	1/1	1/1
chr14_21043	RNASE7	rs1243469	C:0.1789	missense_var	603.00	116.00	H/Y	Cat/Tat	tolerated(1)	benign(0)	1/1	1/1	0/1	0/1	1/1	1/1	1/1	1/1	1/1	1/1
chr14_21057	RNASE8	rs12437266	C:0.4537	missense_var	99.00	10.00	P/S	Ccc/Tcc	tolerated(0.08	benign(0.017)			0/1	0/1	0/1	0/1			0/1	0/1
chr14_21074	ARHGEF40	rs12889267	G:0.1176	missense_var	1004.00	293.00	K/E	Aag/Gag	deleterious(0)	benign(0.175)										
chr14_21078	ARHGEF40	rs61744857	T:0.0601	missense_var	2180.00	685.00	R/C	Cgc/Tgc	tolerated(0.09	benign(0.093)									0/1	0/1
chr14_21081	ARHGEF40	rs7143633	G:0.2196	missense_var	2993.00	956.00	V/L	Gtg/Ctg	tolerated(1)	benign(0)	1/1	1/1			0/1	0/1	1/1	1/1	0/1	0/1
chr14_21082	ARHGEF40	rs756140150		missense_var	3554.00	1143.00	R/W	Cgg/Tgg	deleterious(0)	possibly_dam	0/1	0/1								
chr14_21082	ARHGEF40	rs1958396	T:0.1408	missense_var	3693.00	1189.00	L/S	tTa/tCa	tolerated(0.42	benign(0.003)	1/1	1/1			0/1	0/1	1/1	1/1	1/1	1/1
chr14_21155	OR5AU1	rs7145814	T:0.2768	missense_var	933.00	299.00	I/V	Atc/Gtc	tolerated(1)	benign(0)	0/1	0/1					1/1	1/1	0/1	0/1
chr14_21155	OR5AU1	rs4982419	G:0.3353	missense_var	387.00	117.00	L/F	Ctc/Ttc	deleterious(0)	probably_dam	0/1	0/1					1/1	1/1	0/1	0/1
chr14_21301	RPGRIP1	rs1040904	A:0.0583	missense_var	287.00	96.00	P/Q	cCg/cAg	tolerated(0.7)	benign(0)										
chr14_21302	RPGRIP1	rs6571751	G:0.4772	missense_var	574.00	192.00	K/E	Aaa/Gaa	tolerated(1)	benign(0)							1/1	1/1		
chr14_21321	RPGRIP1	rs147586703,	T:0.0006	missense_var	1753.00	585.00	P/S	Cca/Tca	tolerated_low_	benign(0.051)										
chr14_21328	RPGRIP1	rs3748361	C:0.2953	missense_var	3097.00	1033.00	E/Q	Gag/Cag	tolerated(1)	benign(0)		0/1								
chr14_21431	CHD8	rs10467770	T:0.2528	missense_var	237.00	58.00	V/M	Gtg/Atg	deleterious_lo	benign(0.026)	0/1	0/1	0/1	0/1			0/1	0/1		
chr14_21523	SALL2	rs1263810	G:0.2442	missense_var	2531.00	746.00	R/G	Cgg/Ggg	tolerated(1)	benign(0)	0/1	0/1	0/1	0/1			1/1	1/1	1/1	1/1
chr14_21525	SALL2	rs144885457	A:0.0068	missense_var	798.00	168.00	P/L	cCg/cTg	deleterious_lo	probably_damaging(1)					0/1	0/1				
chr14_21525	SALL2	rs1263811	G:0.2011	missense_var	659.00	122.00	S/P	Tca/Cca	tolerated(0.06	possibly_damaging(0.498)			0/1	0/1			0/1	0/1		
chr14_21569	OR10G3	rs34162196	T:0.0357	missense_var	1166.00	251.00	V/M	Gtg/Atg	tolerated(0.1)	benign(0.107)							0/1	0/1		
chr14_21570	OR10G3	rs17792778	T:0.2887	missense_var	632.00	73.00	S/G	Agc/Ggc	tolerated(0.59	benign(0)	1/1	1/1	1/1	1/1	0/1	0/1	1/1	1/1	0/1	0/1
chr14_21622	TRAV1-1	rs1063365	G:0.2903	missense_var	266.00	67.00	A/G	gCt/gGt	tolerated(0.3)	benign(0.345)			0/1	0/1			0/1	0/1		
chr14_21634	OR10G2	rs41307110		missense_var	804.00	236.00	T/A	Act/Gct	tolerated(1)	benign(0)	1/1	1/1	1/1	1/1	1/1	1/1	0/1	0/1	0/1	0/1
chr14_21634	OR10G2	rs200792644	T:0.0877	missense_var	780.00	228.00	N/H	Aat/Cat	tolerated(0.54	benign(0)	1/1	1/1	1/1	1/1	1/1	1/1	0/1	0/1	0/1	0/1
chr14_21634	OR10G2	rs12894405	C:0.4515	missense_var	723.00	209.00	G/R	Ggg/Agg	deleterious(0)	benign(0.141)			0/1	0/1	0/1	0/1	0/1	0/1		
chr14_21634	OR10G2	rs35963889	G:0.3808	missense_var	658.00	187.00	P/R	cCc/cGc	deleterious(0.	probably_damaging(1)			0/1	0/1	0/1	0/1	0/1	0/1		
chr14_21634	OR10G2	rs10138694	C:0.4505	missense_var	505.00	136.00	R/H	cGc/cAc	tolerated(0.51	benign(0.005)			0/1	0/1	0/1	0/1	0/1	0/1		
chr14_21634	OR10G2	rs41314525	A:0.2342	missense_var	352.00	85.00	L/R	cTg/cGg	tolerated(0.16	benign(0)	1/1	1/1	1/1	1/1	1/1	1/1	0/1	0/1	0/1	0/1
chr14_21665	OR4E2	rs2874103	G:0.2794	missense_var	647.00	118.00	M/V	Atg/Gtg	deleterious(0)	possibly_damaging(0.477)					0/1	0/1				
chr14_21665	OR4E2	rs970382	A:0.2786	missense_var	996.00	234.00	R/Q	cGg/cAg	tolerated(0.08	benign(0.033)					0/1	0/1				
chr14_21665	OR4E2	rs61732411	T:0.1396	missense_var	1017.00	241.00	S/L	tCg/tTg	deleterious(0.	benign(0.031)			0/1	0/1			0/1	0/1		
chr14_21736	TRAV4	rs148839584	T:0.0042	missense_var	129.00	21.00	T/I	aCc/aTc	tolerated(0.38	benign(0.235)										
chr14_21768	TRAV6	rs766738	C:0.1841	missense_var	28.00	7.00	S/R	agC/agG	tolerated_low_	benign(0.003)	1/1	1/1	0/1	0/1	0/1	0/1	1/1	1/1	1/1	1/1
chr14_21783	TRAV7	rs34078918	A:0.0108	missense_var	295.00	99.00	V/M	Gtg/Atg	deleterious(0.	benign(0.407)							0/1	0/1		
chr14_21825	TRAV10	rs34740210	A:0.0118	missense_var	277.00	81.00	G/R	Gga/Aga	deleterious(0.	probably_damaging(0.971)			0/1	0/1						
chr14_21841	TRAV12-1	rs61742979	C:0.0483	missense_var	332.00	110.00	V/A	gTg/gCg	tolerated(1)	benign(0)			0/1	0/1	0/1	0/1				
chr14_21869	TRAV13-1	rs876093	G:0.3195	missense_var	207.00	62.00	G/R	Gga/Aga	deleterious(0.	benign(0.315)	0/1	0/1	1/1	1/1	0/1	0/1	0/1	0/1		
chr14_21888	TRAV12-2	rs17182881	T:0.2540	missense_var	254.00	50.00	G/V	gGt/gTt	tolerated(0.11	benign(0.219)			0/1	0/1	0/1	0/1				
chr14_21888	TRAV12-2	rs10483261	T:0.3980	missense_var	314.00	70.00	F/S	tTc/tCc	tolerated(1)	benign(0)	0/1	0/1	1/1	1/1	0/1	0/1	0/1	0/1		
chr14_21894	TRAV8-4	rs12895616	A:0.3411	missense_var	339.00	71.00	S/T	Tca/Aca	tolerated(0.24	benign(0.134)	0/1	0/1	0/1	0/1			0/1	0/1		
chr14_21894	TRAV8-4	rs12890675	G:0.3411	missense_var	343.00	72.00	A/G	gCg/gGg	tolerated(1)	benign(0)	0/1	0/1	0/1	0/1			0/1	0/1		
chr14_21918	TRAV13-2	rs34656082	T:0.1020	missense_var	87.00	6.00	A/V	gCt/gTt	deleterious(0)	possibly_damaging(0.625)			0/1	0/1			0/1	0/1	0/1	0/1
chr14_21924	TRAV14DV4	rs10140810	C:0.4655	missense_var	224.00	50.00	P/Q	cCa/cAa	tolerated(0.06	benign(0.005)	1/1	1/1	0/1	0/1	1/1	1/1	0/1	0/1	0/1	0/1
chr14_21924	TRAV14DV4	rs8021861	C:0.4659	missense_var	301.00	76.00	Q/E	Cag/Gag	tolerated(0.53	benign(0.003)	1/1	1/1	0/1	0/1	1/1	1/1	0/1	0/1	0/1	0/1
chr14_21941	TRAV9-2	rs2178779	A:0.4996	missense_var	117.00	21.00	D/N	Gat/Aat	tolerated(0.06	benign(0.105)	0/1	0/1	1/1	1/1	0/1	0/1	1/1	1/1	1/1	1/1
chr14_21941	TRAV9-2	rs17112412	G:0.0224	missense_var	166.00	37.00	F/C	tTc/tGc	tolerated(0.19	possibly_damaging(0.753)					0/1	0/1				
chr14_21965	TRAV12-3	rs199963360	C:0.0006	missense_var	80.00	8.00	L/F	ttA/ttC	tolerated(0.19	benign(0.018)										
chr14_21979	TRAV8-6	rs2075484	G:0.2526	missense_var	449.00	77.00	E/K	Gaa/Aaa	tolerated(1)	benign(0)	1/1	1/1	1/1	1/1			0/1	0/1	1/1	1/1
chr14_21979	TRAV8-6	rs2075485	A:0.2981	missense_var	452.00	78.00	S/G	Agc/Ggc	tolerated(1)	benign(0)	1/1	1/1	1/1	1/1			0/1	0/1	1/1	1/1
chr14_22003	TRAV18	rs778281720		missense_var	418.00	105.00	V/M	Gtg/Atg	tolerated(0.09	possibly_dam	0/1	0/1								
chr14_22086	TRAV23DV6	rs2272549	C:0.1727	missense_var	184.00	47.00	S/P	Tca/Cca	deleterious(0.	benign(0.099)			0/1	0/1			0/1	0/1		
chr14_22086	TRAV23DV6	rs372076335		missense_var	353.00	103.00	S/L	tCg/tTg	tolerated(1)	benign(0)	1/1	1/1	1/1	1/1	1/1	1/1	1/1	1/1	1/1	1/1
chr14_22096	TRDV1	rs61730868	T:0.0082	missense_var	38.00	13.00	A/V	gCg/gTg	tolerated(0.3)	benign(0.03)										
chr14_22124	TRAV26-1	rs2272550	A:0.2622	missense_var	282.00	25.00	T/P	Acc/Ccc	tolerated(0.31	benign(0.005)	0/1	0/1	1/1	1/1	0/1	0/1	0/1	0/1	0/1	0/1
chr14_22132	TRAV8-7	rs3811291	G:0.3976	missense_var	208.00	70.00	K/E	Aag/Gag	tolerated(0.06	benign(0.005)			0/1	0/1	1/1	1/1			1/1	1/1
chr14_22148	TRAV27	rs34355797	A:0.0903	missense_var	392.00	99.00	T/P	Act/Cct	tolerated(1)	benign(0)	0/1	0/1	1/1	1/1	1/1	1/1	0/1	0/1	1/1	1/1

chr14_22168	TRAV30	rs11157435	C:0.3213	missense_var	237.00	79.00	D/E	gaC/gaA	tolerated(1)	benign(0.015)	0/1	0/1					0/1	0/1		
chr14_22227	TRAV36DV7	rs4982579	C:0.1234	missense_var	291.00	92.00	F/S	tTc/tCc	tolerated(0.4)	benign(0.007)	0/1	0/1								
chr14_22227	TRAV36DV7	rs12889091	A:0.4960	missense_var	335.00	107.00	I/V	Atc/Gtc	tolerated(0.1)	benign(0)							0/1			
chr14_22272	TRAV38-1	rs2075501	G:0.3450	missense_var	152.00	50.00	N/S	aAt/aGt	tolerated(1)	benign(0)	0/1	0/1	0/1	0/1			0/1	0/1	0/1	0/1
chr14_22422	TRDV2	rs2072615	A:0.1470	missense_var	281.00	36.00	V/I	Gtc/Atc	tolerated(0.11	benign(0.026)										
chr14_22422	TRDV2	rs2072616	C:0.4673	missense_var	370.00	65.00	M/I	atG/atC	tolerated(0.06	benign(0.024)	0/1	0/1	1/1	1/1					0/1	0/1
chr14_22478	TRAJ57	rs1872159	T:0.1841	missense_var	43.00	15.00	T/M	aCg/aTg		unknown(0)									1/1	1/1
chr14_22502	TRAJ38	rs17118924	G:0.0477	missense_var	51.00	18.00	A/G	gCa/gGa		unknown(0)										
chr14_22503	TRAJ37	rs2170810	G:0.4059	missense_var	8.00	4.00	S/G	Agc/Ggc		unknown(0)									1/1	1/1
chr14_22519	TRAJ24	rs1483979	C:0.2204	missense_var	26.00	9.00	F/L	ttC/ttG		unknown(0)	1/1	1/1			1/1	1/1	1/1	1/1	1/1	1/1
chr14_22530	TRAJ14	rs183820284	A:0.0004	missense_var	35.00	12.00	R/K	aGa/aAa		unknown(0)										
chr14_22531	TRAJ13	rs227003	T:0.1881	missense_var	37.00	13.00	T/I	aCt/aTt		unknown(0)							0/1	0/1		
chr14_22535	TRAJ9	rs143973826	C:0.0074	missense_var	47.00	16.00	L/P	cTa/cCa		unknown(0)	1/1	0/1								
chr14_22540	TRAJ5	rs3811196	T:0.0254	missense_var	7.00	3.00	T/M	aCg/aTg		unknown(0)					1/1	1/1			0/1	0/1
chr14_22633	OR6J1	rs1681596	A:0.2097	missense_var	1117.00	279.00	V/L	Gtg/Ttg	deleterious(0.	possibly_damaging(0.558)					0/1	0/1	1/1	1/1		
chr14_22633	OR6J1	rs1681597	G:0.2097	missense_var	1103.00	274.00	L/S	tTg/tCg	tolerated(1)	benign(0)					0/1	0/1	1/1	1/1		
chr14_22634	OR6J1	rs1753430	G:0.4205	missense_var	1030.00	250.00	S/P	Tct/Cct	tolerated(0.2)	benign(0.107)	0/1	0/1	1/1	1/1					1/1	1/1
chr14_22634	OR6J1	rs3829405	T:0.1959	missense_var	959.00	226.00	R/H	cGc/cAc	tolerated(0.24	benign(0.001)					0/1	0/1	0/1	0/1		
chr14_22634	OR6J1	rs1681598	T:0.0142	missense_var	955.00	225.00	V/M	Gtg/Atg	deleterious(0.	benign(0.236)							0/1	0/1		
chr14_22634	OR6J1	rs45470397	T:0.0142	missense_var	695.00	138.00	S/Y	tCt/tAt	tolerated(0.05	benign(0.003)							0/1	0/1		
chr14_22634	OR6J1	rs3751482	C:0.2093	missense_var	652.00	124.00	T/A	Acc/Gcc	tolerated(1)	benign(0)					0/1	0/1	1/1	1/1		
chr14_22634	OR6J1	rs3751484	T:0.1951	missense_var	644.00	121.00	R/H	cGt/cAt	deleterious(0)	benign(0.052)					0/1	0/1	0/1	0/1		
chr14_22634	OR6J1	rs45494902	A:0.0148	missense_var	643.00	121.00	R/C	Cgt/Tgt	deleterious(0.	benign(0.033)							0/1	0/1		
chr14_22767	OXA1L	rs8572	C:0.2282	missense_var	311.00	104.00	A/V	gCa/gTa	tolerated_low	benign(0.059)	1/1	1/1	1/1	1/1	1/1	1/1	1/1	1/1	1/1	1/1
chr14_22813	SLC7A7	rs11568438	A:0.0042	missense_var	798.00	91.00	A/V	gCg/gTg	deleterious(0)	possibly_dam	0/1	0/1								
chr14_22829	MRPL52	rs1135641	G:0.4858	missense_var	44.00	5.00	G/V	gGg/gTg	tolerated_low	benign(0.144)	0/1	0/1	1/1	1/1	0/1	0/1	0/1	0/1		
chr14_22830	MRPL52	rs4982685	T:0.1324	missense_var	86.00	19.00	V/A	gTa/gCa	tolerated(1)	benign(0)	1/1	1/1	1/1	1/1	1/1	1/1	0/1	0/1	1/1	1/1
chr14_22836	MMP14	rs1042703	C:0.1324	missense_var	283.00	8.00	P/S	Ccc/Tcc	tolerated(1)	benign(0)			1/1				0/1	0/1		1/1
chr14_22843	MMP14	rs1042704	A:0.1098	missense_var	1078.00	273.00	D/N	Gat/Aat	tolerated(0.18	benign(0.006)										
chr14_22901	RBM23	rs1127066	C:0.2149	missense_var	1480.00	428.00	F/L	ttC/ttG	tolerated(0.14	benign(0.047)	0/1	0/1					1/1	1/1		
chr14_22902	RBM23	rs199926651	C:0.0010	missense_var	1346.00	384.00	T/A	Act/Gct	tolerated(1)	benign(0)									0/1	0/1
chr14_22906	RBM23	rs72681969,rs	A:0.0144	missense_var	437.00	81.00	R/W	Cgg/Tgg	tolerated(0.06	possibly_damaging(0.551)							1/1	1/1		
chr14_23034	PSMB5	rs11543947	A:0.0359	missense_var	334.00	24.00	R/C	Cgt/Tgt	deleterious(0.	benign(0)	0/1	0/1								
chr14_23042	PSMB11	rs34457782	A:0.0084	missense_var	204.00	49.00	G/S	Ggc/Agc	deleterious(0)	probably_damaging(1)			0/1	0/1						
chr14_23049	CDH24	rs116261083	T:0.0286	missense_var	1759.00	500.00	R/H	cGc/cAc	tolerated(0.42	benign(0.031)					0/1	0/1				
chr14_23080	ACIN1	rs3751501	A:0.0517	missense_var	1761.00	478.00	S/F	tCt/tTt	deleterious_lo	possibly_damaging(0.656)			0/1	0/1						
chr14_23080	ACIN1	rs1885097	G:0.4283	missense_var	1727.00	467.00	S/P	Tct/Cct	tolerated_low	benign(0.001)	0/1	0/1	1/1	1/1					0/1	0/1
chr14_23080	ACIN1	rs941719,COS	C:0.0004	missense_var	1667.00	447.00	A/P	Gct/Cct	tolerated_low	benign(0)	1/1	1/1	1/1	1/1	1/1	1/1	1/1	1/1	1/1	1/1
chr14_23080	ACIN1	rs3811182	T:0.4992	missense_var	1261.00	311.00	I/M	atA/atG	tolerated_low	benign(0)	0/1	0/1	1/1	1/1	0/1	0/1			0/1	0/1
chr14_23276	HOMEZ	rs10131813		missense_var	1069.00	302.00	A/T	Gcc/Acc	tolerated(1)	benign(0)	0/1	0/1	0/1	0/1	0/1	0/1			0/1	0/1
chr14_23308	BCL2L2	rs910332	A:0.0006	missense_var	627.00	133.00	Q/R	cAg/cGg	tolerated(0.19	benign(0)	1/1	1/1	1/1	1/1	1/1	1/1	1/1	1/1	1/1	1/1
chr14_23347	SLC22A17	rs186221654	C:0.0016	missense_var	1618.00	372.00	M/V	Atg/Gtg	tolerated(0.12	benign(0.33)			0/1	0/1						
chr14_23360	EFS	rs2231798	C:0.4557	missense_var	627.00	7.00	T/A	Acc/Gcc	tolerated(1)	benign(0)	0/1	0/1	0/1	0/1						
chr14_23375	IL25	rs148309201	A:0.0026	missense_var	779.00	174.00	R/H	cGt/cAt	deleterious(0)	probably_dam	0/1	0/1								
chr14_23390	MYH6	rs28730771	T:0.0729	missense_var	3459.00	1130.00	A/T	Gcc/Acc	tolerated(0.33	possibly_dam	0/1	0/1	0/1	0/1					0/1	0/1
chr14_23392	MYH6	rs365990	G:0.3748	missense_var	3373.00	1101.00	V/A	gTg/gCg	tolerated(1)	benign(0)			0/1	0/1	0/1	0/1			0/1	0/1
chr14_23407	MYH6	rs28711516,C	T:0.0567	missense_var	237.00	56.00	G/R	Gga/Aga	deleterious(0)	probably_damaging(0.931)										
chr14_23523	ZFHX2	rs223124	G:0.2546	missense_var	6765.00	2137.00	S/R	Agt/Cgt	tolerated(0.08	benign(0.034)	0/1	0/1	0/1	0/1			0/1	0/1	0/1	0/1
chr14_23525	ZFHX2	rs4982766	G:0.3838	missense_var	4990.00	1545.00	V/A	gTt/gCt	tolerated(0.89	benign(0)	0/1	0/1	0/1	0/1			0/1	0/1	0/1	0/1
chr14_23533	ZFHX2	rs3742489	T:0.0940	missense_var	2004.00	550.00	P/T	Cca/Aca	tolerated(0.06	benign(0.001)							0/1	0/1		
chr14_23534	ZFHX2	rs145454038	T:0.0004	missense_var	1209.00	285.00	C/S	Tgc/Agc	tolerated_low	benign(0)					0/1	0/1				
chr14_23557	THTPA	rs34015250	G:0.1392	missense_var	1263.00	176.00	H/R	cAc/cGc	tolerated(0.62	benign(0.042)							0/1		0/1	0/1
chr14_23563	AP1G2	rs12897422	A:0.0435	missense_var	1886.00	377.00	S/F	tCc/tTc	deleterious(0)	probably_damaging(0.944)							0/1	0/1		
chr14_23988	DHRS4L2	rs2273946	G:0.3261	missense_var	132.00	2.00	Q/H	caG/caC	tolerated_low	benign(0)	1/1	1/1	1/1	1/1	1/1	1/1			0/1	0/1
chr14_23988	DHRS4L2	rs147724387	C:0.0022	missense_var	171.00	15.00	K/N	aaG/aaC	tolerated(0.55	benign(0.007)									0/1	0/1
chr14_23989	DHRS4L2	rs2273947	T:0.1883	missense_var	181.00	19.00	M/L	Atg/Ttg	tolerated(0.19	benign(0.017)	1/1	1/1								
chr14_23990	DHRS4L2	rs45579231	C:0.0076	missense_var	307.00	61.00	V/L	Gtc/Ctc	tolerated(0.08	benign(0.037)							0/1	0/1		
chr14_24000	DHRS4L2	rs1811890	T:0.1288	stop_gained	601.00	159.00	R/*	Cga/Tga					0/1	0/1					0/1	0/1
chr14_24064	CARMIL3	rs117092113	A:0.0146	missense_var	3153.00	1000.00	R/H	cGt/cAt	tolerated(0.2)	possibly_damaging(0.749)										
chr14_24064	CARMIL3	rs10146906	A:0.3486	missense_var	3218.00	1022.00	L/M	Ctg/Atg	tolerated(0.48	benign(0)										

chr14_24103	PCK2	rs35618680,C	A:0.0857	missense_var	1830.00	521.00	R/H	cGc/cAc	tolerated(0.07	benign(0.001)	0/1	0/1								
chr14_24118	DCAF11	rs3825584	A:0.3972	missense_var	1347.00	207.00	R/H	cGt/cAt	tolerated(0.41	benign(0)	0/1	0/1	0/1	0/1	0/1	0/1			1/1	1/1
chr14_24118	DCAF11	rs192332230,	A:0.0002	missense_var	1371.00	215.00	R/H	cGc/cAc	deleterious(0.	probably_damaging(0.974)			0/1	0/1						
chr14_24145	PSME2	rs7146672	T:0.0040	missense_var	906.00	89.00	H/P	cAt/cCt	tolerated(1)	benign(0)	1/1	1/1	1/1		1/1	1/1	1/1	1/1	1/1	
chr14_24148	RNF31	rs45466595	A:0.0006	missense_var	735.00	139.00	E/K	Gag/Aag	tolerated(0.87	benign(0.003)										
chr14_24150	RNF31	rs187811103	T:0.0002	missense_var	1710.00	464.00	R/C	Cgc/Tgc	tolerated(0.13	benign(0)										
chr14_24160	RNF31	rs2277484	A:0.0329	missense_var	3501.00	1061.00	V/I	Gta/Ata	tolerated(0.14	probably_damaging(0.978)					1/1	1/1			1/1	1/1
chr14_24184	IPO4	rs11550452	C:0.3155	missense_var	1915.00	580.00	P/A	Cct/Gct	deleterious(0)	probably_damaging(0.973)			1/1	1/1						
chr14_24184	IPO4	rs7146310	A:0.3179	missense_var	1715.00	513.00	A/V	gCc/gTc	tolerated(0.13	benign(0.015)			1/1	1/1						
chr14_24192	TM9SF1	rs10583	T:0.0547	missense_var	1003.00	215.00	R/H	cGt/cAt	tolerated(0.52	benign(0)										
chr14_24267	RABGGTA	rs729421	T:0.3976	missense_var	1742.00	420.00	T/A	Acg/Gcg	tolerated(1)	benign(0)			1/1		0/1	0/1	1/1			
chr14_24291	DHRS1	rs10134537	A:0.0937	missense_var	999.00	241.00	T/I	aCa/aTa	tolerated(0.11	benign(0.157)							0/1	0/1		
chr14_24302	NOP9	rs4280164	A:0.1504	missense_var	1016.00	308.00	S/N	aGt/aAt	deleterious(0.	possibly_damaging(0.639)										
chr14_24310	LTB4R2	rs77369205	T:0.0042	missense_var	308.00	46.00	A/V	gCg/gTg	tolerated(0.61	benign(0.033)			0/1	0/1						
chr14_24336	RIPK3	rs3212254,C(	T:0.1202	missense_var	1694.00	492.00	P/Q	cCa/cAa	tolerated_low_	probably_dam	0/1	0/1	0/1	0/1						
chr14_24369	NFATC4	rs2229309,C[	C:0.2804	missense_var	812.00	223.00	G/A	gGc/gCc	tolerated_low_	benign(0)	0/1	0/1	0/1	0/1			0/1	0/1		
chr14_24376	NFATC4	rs7149586	C:0.3580	missense_var	2731.00	863.00	S/P	Tcc/Ccc	tolerated_low_	benign(0)	0/1	0/1	0/1	0/1			0/1	0/1		
chr14_24409	NYNRIN	rs12437434	T:0.0863	missense_var	1688.00	457.00	T/M	aCg/aTg	tolerated_low_	benign(0)	0/1	0/1	1/1	1/1						
chr14_24409	NYNRIN			missense_var	1781.00	488.00	Q/L	cAa/cTa	deleterious_lc	benign(0)		0/1								
chr14_24414	NYNRIN	rs8017377	A:0.2444	missense_var	3250.00	978.00	A/T	Gct/Act	tolerated_low_	benign(0)	0/1	0/1							0/1	0/1
chr14_24432	KHNYN	rs3742520	C:0.4052	missense_var	921.00	261.00	K/T	aAa/aCa	deleterious(0.	benign(0.007)	0/1	0/1	1/1	1/1			0/1	0/1		
chr14_24432	KHNYN	rs7151995	G:0.0405	missense_var	948.00	270.00	W/L	tGg/tTg	tolerated(0.7)	benign(0)	1/1	1/1	1/1	1/1	1/1	1/1	1/1	1/1	1/1	1/1
chr14_24440	SDR39U1	rs1043831	T:0.2348	missense_var	843.00	270.00	Q/R	cAg/cGg	tolerated(0.24	benign(0)	1/1	1/1	1/1	1/1	1/1	1/1		1/1	1/1	1/1
chr14_24440	SDR39U1	rs3211056	C:0.2348	missense_var	730.00	232.00	L/F	ttG/ttC	tolerated(1)	benign(0.037)	1/1	1/1	1/1	1/1	1/1	1/1	1/1	1/1	1/1	1/1
chr14_24441	SDR39U1	rs11625819	T:0.2348	missense_var	269.00	79.00	I/L	Atc/Ctc	tolerated(0.75	benign(0)	1/1	1/1	1/1		1/1		1/1	1/1	1/1	1/1
chr14_24505	CMA1	rs137883267	A:0.0004	missense_var	706.00	226.00	P/S	Ccc/Tcc	tolerated(0.06	probably_damaging(1)										
chr14_24607	GZMH	rs558566520	T:0.0002	missense_var	482.00	146.00	G/D	gGc/gAc	deleterious(0)	probably_dam	0/1	0/1								
chr14_24607	GZMH	rs140193391		missense_var	325.00	94.00	P/A	Ccc/Gcc	tolerated(1)	benign(0)	0/1	0/1								
chr14_24607	GZMH	rs20545	T:0.0575	missense_var	296.00	84.00	R/Q	cGg/cAg	tolerated(0.1)	benign(0.026)	0/1	0/1								
chr14_24631	GZMB	rs2236338,C[	G:0.2919	missense_var	846.00	247.00	Y/H	Tac/Cac	tolerated(0.24	benign(0.005)	1/1	1/1								
chr14_24632	GZMB	rs11539752,C	C:0.3019	missense_var	387.00	94.00	P/A	Ccc/Gcc	tolerated(1)	benign(0)	1/1	1/1								
chr14_24632	GZMB	rs8192917,C[	C:0.3193	missense_var	271.00	55.00	R/Q	cGa/cAa	tolerated(0.34	benign(0.042)			1/1	1/1	1/1	1/1	1/1	1/1	1/1	1/1
chr14_26448	NOVA1			missense_var	1208.00	297.00	V/G	gTt/gGt	deleterious(0.	possibly_damaging(0.528)										
chr14_29663	PRKD1			stop_gained	783.00	218.00	S/*	tCa/tAa												0/1
chr14_30630	SCFD1	rs229150	A:0.4223	missense_var	415.00	63.00	K/R	aAg/aGg	tolerated(0.12	benign(0.123)	0/1	0/1	0/1	0/1	1/1	1/1			0/1	0/1
chr14_30885	COCH	rs1045644	G:0.4113	missense_var	1111.00	352.00	T/S	aCt/aGt	tolerated(0.61	benign(0)	0/1	0/1	1/1	1/1	0/1	0/1	0/1	0/1	0/1	0/1
chr14_30912	STRN3	rs2273171	C:0.3890	missense_var	1609.00	471.00	N/S	aAt/aGt	tolerated(0.05	benign(0.138)	0/1		1/1	1/1	0/1	0/1	0/1	0/1	0/1	0/1
chr14_31114	HECTD1	rs61976859	T:0.0613	missense_var	6142.00	1885.00	E/K	Gaa/Aaa	tolerated_low_	benign(0.027)										
chr14_31129	HECTD1	rs75345169,C	C:0.0068	missense_var	4765.00	1426.00	I/V	Att/Gtt	tolerated_low_	benign(0)									0/1	0/1
chr14_31321	HEATR5A	rs28396248	G:0.0909	missense_var	3973.00	1263.00	N/T	aAt/aCt	tolerated(0.76	benign(0.003)	0/1	0/1			0/1	0/1				
chr14_31349	HEATR5A	rs3736918	C:0.1949	missense_var	2841.00	886.00	V/M	Gtg/Atg	deleterious(0)	probably_damaging(0.994)					0/1	0/1	1/1	1/1		1/1
chr14_31388	HEATR5A	rs374697149,	T:0.0004	missense_var	1051.00	289.00	R/Q	cGa/cAa	deleterious(0.	possibly_damaging(0.821)										
chr14_31389	HEATR5A	rs7157977,C(	T:0.3109	missense_var	960.00	259.00	A/T	Gcc/Acc	tolerated(0.24	benign(0.003)							1/1		1/1	1/1
chr14_31483	GPR33	rs17097921	G:0.0298	stop_gained	670.00	140.00	R/*	Cga/Tga			1/1	1/1	0/1	0/1	1/1	1/1	1/1	1/1	1/1	1/1
chr14_32545	AKAP6	rs3742926	T:0.1759	missense_var	1180.00	337.00	A/V	gCt/gTt	tolerated(0.32	benign(0)							0/1	0/1	0/1	0/1
chr14_32545	AKAP6			missense_var	1314.00	382.00	D/H	Gac/Cac	deleterious(0.	probably_damaging(0.996)										
chr14_32545	AKAP6	rs17099240,C	G:0.1761	missense_var	1393.00	408.00	N/S	aAt/aGt	tolerated(1)	benign(0)							0/1	0/1	0/1	0/1
chr14_32546	AKAP6	rs45477296	T:0.0347	missense_var	1708.00	513.00	R/L	cGg/cTg	tolerated(0.08	benign(0.018)							0/1	0/1		
chr14_32600	AKAP6	rs34572259	A:0.0030	missense_var	2844.00	892.00	E/K	Gag/Aag	deleterious(0.	probably_damaging(0.986)										
chr14_32822	AKAP6	rs11845640	T:0.2654	missense_var	4645.00	1492.00	A/V	gCg/gTg	tolerated_low_	benign(0.005)					1/1	1/1				
chr14_32823	AKAP6	rs1051695	A:0.2897	missense_var	6273.00	2035.00	N/D	Aac/Gac	tolerated(0.81	benign(0)	0/1	0/1	0/1	0/1	1/1	1/1	0/1	0/1		
chr14_32824	AKAP6	rs4647899	A:0.2684	missense_var	6682.00	2171.00	F/Y	tTc/tAc	tolerated_low_	benign(0.006)	0/1	0/1	0/1	0/1			0/1	0/1		
chr14_33799	NPAS3	rs12434716	C:0.1216	missense_var	1654.00	552.00	A/P	Gcg/Ccg	tolerated(0.48	benign(0)							1/1	1/1		
chr14_33931	EGLN3	rs759224695,COSM575004		missense_var	750.00	141.00	G/S	Ggt/Agt	tolerated(0.13	benign(0.368)									0/1	0/1
chr14_35180	AL121594.3	rs547075844	A:0.0002	missense_var	1583.00	401.00	R/H	cGt/cAt	tolerated(0.25	benign(0)										
chr14_35534	INSM2	rs1958260	A:0.0034	missense_var	828.00	206.00	D/G	gAc/gGc	tolerated(1)	benign(0)		1/1	1/1	1/1	1/1	1/1	1/1		1/1	1/1
chr14_35627	RALGAPA1	rs140406088	A:0.0276	missense_var	7029.00	2217.00	P/L	cCt/cTt	deleterious(0.	probably_damaging(0.999)									0/1	0/1
chr14_35683	RALGAPA1			missense_var	4547.00	1390.00	T/A	Acc/Gcc	tolerated_low_	benign(0)			0/1	0/1	0/1	0/1			0/1	0/1
chr14_36314	MBIP	rs199518397	A:0.0002	stop_gained	599.00	171.00	Q/*	Caa/Taa							0/1	0/1				
chr14_36320	MBIP	rs3168891	T:0.0731	missense_var	154.00	22.00	R/S	agA/agC	tolerated_low_	benign(0)	1/1	1/1	1/1	1/1	1/1	1/1	1/1	1/1	1/1	1/1

chr14_363205	MBIP	rs2899849	A:0.2141	missense_var	108.00	7.00	L/H	cTt/cAt	tolerated_low	benign(0.003)	1/1	1/1	1/1	1/1	1/1	1/1	1/1	1/1	1/1	1/1
chr14_366665	PAX9	rs4904210,CN	C:0.3317	missense_var	943.00	240.00	A/P	Gcg/Ccg	deleterious_lc	benign(0)	0/1	0/1	0/1	0/1	0/1	0/1	0/1	0/1	0/1	0/1
chr14_375914	FOXA1	rs33984772,C	T:0.0383	missense_var	1405.00	448.00	S/N	aGc/aAc	tolerated(0.35	benign(0.005)							0/1	0/1		
chr14_375925	FOXA1	rs7144658,CC	C:0.4169	missense_var	309.00	83.00	A/T	Gcc/Acc	tolerated(0.24	benign(0.013)	1/1	1/1	1/1	1/1	1/1	1/1	0/1	0/1	1/1	1/1
chr14_376223	TTC6	rs181566041	G:0.0062	missense_var	1180.00	108.00	E/G	gAg/gGg	tolerated_low	benign(0.006)			0/1	0/1						
chr14_376227	TTC6			missense_var	1588.00	244.00	S/T	aGc/aCc	tolerated_low	benign(0)					0/1	0/1				
chr14_376227	TTC6	rs117333192	A:0.0096	missense_var	1636.00	260.00	G/E	gGg/gAg	tolerated_low	benign(0.039)										
chr14_376228	TTC6	rs117711529	C:0.0315	missense_var	1661.00	268.00	E/D	gaG/gaC	tolerated(0.26	possibly_damaging(0.783)							0/1	0/1		
chr14_376228	TTC6	rs140258566	T:0.0315	missense_var	1662.00	269.00	S/C	Agc/Tgc	deleterious(0.	possibly_damaging(0.852)							0/1	0/1		
chr14_376228	TTC6	rs117168426	C:0.0315	missense_var	1663.00	269.00	S/T	aGc/aCc	tolerated(0.72	benign(0.283)							0/1	0/1		
chr14_377147	TTC6	rs78042024	A:0.0044	missense_var	2545.00	563.00	G/E	gGg/gAg	tolerated(0.84	benign(0.018)			0/1	0/1	0/1	0/1				
chr14_377249	TTC6	rs34543736	G:0.0343	missense_var	2623.00	589.00	Y/C	tAt/tGt	tolerated(0.23	benign(0)							0/1	0/1		
chr14_377490	TTC6	rs141232166	G:0.0038	missense_var	3414.00	853.00	I/V	Att/Gtt	tolerated(1)	benign(0.006)			0/1	0/1						
chr14_377491	TTC6	rs12586727	G:0.2123	missense_var	3468.00	871.00	I/V	Ata/Gta	tolerated(0.35	benign(0)	0/1	0/1	0/1	0/1	0/1	0/1			1/1	1/1
chr14_378047	TTC6	rs12896790	G:0.0507	missense_var	321.00	87.00	I/S	aTt/aGt	tolerated(0.12	benign(0.029)							0/1			
chr14_378087	TTC6	rs4901284	T:0.2933	missense_var	771.00	237.00	T/I	aCa/aTa	tolerated(0.39	benign(0)					0/1	0/1		0/1		
chr14_390869	SEC23A	rs8018720	G:0.2212	missense_var	1149.00	211.00	L/V	Ctt/Gtt	tolerated(0.51	benign(0)	0/1	0/1			1/1	1/1	0/1	0/1	1/1	1/1
chr14_391810	PNN	rs2180792	A:0.0970	missense_var	1388.00	441.00	T/S	Acc/Tcc	tolerated_low	benign(0.006)	1/1	1/1	1/1	1/1	1/1	1/1	0/1	0/1	1/1	1/1
chr14_391817	PNN	rs13021	G:0.1260	missense_var	2078.00	671.00	S/G	Agt/Ggt	deleterious_lc	probably_dam	0/1	0/1	1/1	1/1						
chr14_392469	MIA2	rs11845046	G:0.2382	missense_var	617.00	141.00	I/M	atA/atG	tolerated(0.23	benign(0.005)	0/1	0/1	1/1	1/1	1/1	1/1			0/1	0/1
chr14_392473	MIA2	rs762661827,COSM176762		missense_var	948.00	252.00	R/W	Cgg/Tgg	deleterious(0.	possibly_dam	0/1	0/1								
chr14_392527	MIA2			missense_var	1768.00	525.00	V/D	gTc/gAc	tolerated(0.23	benign(0.003)					0/1	0/1				
chr14_392528	MIA2	rs10134365	C:0.3107	missense_var	1833.00	547.00	D/H	Gat/Cat	deleterious(0.	benign(0.003)	0/1	0/1	1/1	1/1	1/1	1/1			0/1	0/1
chr14_392674	CTAGE5	rs7140561	T:0.0875	missense_var	353.00	6.00	V/A	gTt/gCt	tolerated(1)	benign(0)			1/1	1/1	1/1			0/1		
chr14_393084	CTAGE5	rs1950952	G:0.4641	missense_var	1429.00	365.00	E/Q	Gaa/Caa	tolerated(0.15	benign(0.12)	0/1	0/1							0/1	0/1
chr14_393488	CTAGE5	rs1140952	G:0.3846	missense_var	2446.00	704.00	I/V	Atc/Gtc	tolerated(0.99	benign(0.001)	0/1	0/1	1/1	1/1	1/1	1/1	0/1	0/1	0/1	0/1
chr14_445049	FSCB	rs11621923		missense_var	2294.00	668.00	A/S	Gct/Tct	tolerated(0.29	benign(0.051)	0/1	0/1			1/1	1/1			1/1	1/1
chr14_445050	FSCB	rs8009274		missense_var	2216.00	642.00	A/T	Gcc/Acc	tolerated(0.14	benign(0.134)				0/1						
chr14_445057	FSCB	rs3825632	T:0.4401	missense_var	1561.00	423.00	D/E	gaT/gaA	tolerated(1)	benign(0)	0/1	0/1			1/1	1/1			1/1	1/1
chr14_445057	FSCB	rs1959379	A:0.2712	missense_var	1517.00	409.00	P/S	Cca/Tca	tolerated(0.15	benign(0.003)	0/1	0/1	1/1	1/1						
chr14_445058	FSCB	rs3825630	G:0.2712	missense_var	1431.00	380.00	L/P	cTt/cCt	tolerated(0.12	benign(0.003)	0/1	0/1	1/1	1/1						
chr14_445063	FSCB	rs45478391,rs	A:0.1080	missense_var	972.00	227.00	P/L	cCg/cTg	tolerated(0.62	benign(0.015)							0/1	0/1		
chr14_445064	FSCB	rs3809429	T:0.2716	missense_var	877.00	195.00	H/Q	caC/caA	deleterious(0)	benign(0.275)	0/1	0/1	1/1	1/1						
chr14_449344	KLHL28	rs35728857	C:0.0012	missense_var	1291.00	363.00	I/V	Atc/Gtc	tolerated(0.29	benign(0.003)	0/1	0/1								
chr14_449636	TOGARAM1	rs3825629	C:0.1883	missense_var	1429.00	416.00	E/Q	Gaa/Caa	tolerated(1)	benign(0)			0/1	0/1						
chr14_449639	TOGARAM1	rs3742591	G:0.0543	missense_var	1714.00	511.00	L/V	Ctg/Gtg	deleterious(0)	probably_damaging(0.996)			0/1	0/1						
chr14_451753	FANCM	rs1367580	T:0.2322	missense_var	2717.00	878.00	V/L	Gta/Tta	tolerated(1)	benign(0)			1/1	1/1						
chr14_451755	FANCM	rs148871932	G:0.0050	missense_var	2834.00	917.00	I/V	Att/Gtt	tolerated(0.51	benign(0)			0/1	0/1						
chr14_451816	FANCM	rs78211950	G:0.1014	missense_var	4463.00	1460.00	I/V	Ata/Gta	tolerated(0.89	benign(0.007)			0/1	0/1						
chr14_451962	FANCM	rs3736772	G:0.1062	missense_var	5519.00	1812.00	P/A	Ccg/Gcg	tolerated(0.09	benign(0.001)			0/1	0/1						
chr14_452244	MIS18BP1	rs145716748	G:0.0056	missense_var	2644.00	729.00	S/P	Tct/Cct	tolerated(1)	benign(0)			0/1	0/1						
chr14_452268	MIS18BP1	rs34101857	G:0.1070	missense_var	2208.00	583.00	E/D	gaA/gaC	deleterious(0.	benign(0.007)			0/1	0/1						
chr14_452427	MIS18BP1	rs138267500	T:0.0050	missense_var	1093.00	212.00	A/T	Gca/Aca	tolerated(0.29	benign(0)			0/1	0/1						
chr14_469574	MDGA2	rs12590500	A:0.1669	missense_var	1822.00	608.00	V/F	Gtt/Ttt	deleterious(0.	benign(0.222)			0/1	0/1					0/1	0/1
chr14_470969	MDGA2	rs77910307	C:0.0014	missense_var	931.00	311.00	I/V	Att/Gtt	tolerated(0.7)	benign(0.003)									0/1	0/1
chr14_496074	LRR1	rs17121605	A:0.1883	missense_var	611.00	96.00	I/N	aTt/aAt	tolerated(1)	benign(0)										
chr14_496078	LRR1	rs7148147	T:0.1723	missense_var	1009.00	229.00	R/W	Cgg/Tgg	deleterious(0.	possibly_damaging(0.745)										
chr14_496349	DNAAF2	rs2985684	G:0.4932	missense_var	267.00	62.00	E/D	gaG/gaC	tolerated(0.07	possibly_dam	0/1	0/1					1/1	1/1	0/1	0/1
chr14_497958	NEMF	rs142838359	G:0.0010	missense_var	3030.00	860.00	K/N	aaA/aaC	tolerated(0.13	benign(0.001)	0/1	0/1								
chr14_498322	NEMF	rs3100906	T:0.0727	missense_var	1219.00	257.00	S/C	Agc/Tgc	deleterious(0.	probably_dam	1/1	1/1	1/1	1/1	1/1	1/1	1/1	1/1	1/1	1/1
chr14_501142	VCPKMT	rs142528336	C:0.0012	missense_var	567.00	188.00	Y/D	Tat/Gat	deleterious(0)	possibly_damaging(0.852)										
chr14_501163	VCPKMT	rs11157729	T:0.2500	missense_var	193.00	63.00	A/D	gCc/gAc	tolerated(0.27	benign(0.035)			0/1	0/1	0/1	0/1				
chr14_502653	L2HGDH	rs373172891	C:0.0004	missense_var	1572.00	392.00	I/V	Att/Gtt	tolerated(0.36	benign(0.122)				0/1						
chr14_503120	L2HGDH	rs2275591	A:0.4273	missense_var	451.00	18.00	L/R	cTt/cGt	tolerated_low	benign(0.01)	0/1	0/1	0/1	0/1	0/1	0/1				
chr14_503214	ATP5S	rs2275592	C:0.4311	missense_var	433.00	18.00	P/L	cCg/cTg	tolerated_low	benign(0)	0/1	0/1	0/1	0/1	0/1	0/1				
chr14_503301	CDKL1	rs9323183	C:0.0200	missense_var	1016.00	330.00	L/V	Cta/Gta	deleterious(0.	possibly_damaging(0.492)							0/1	0/1		
chr14_503324	CDKL1	rs7161563,CC	G:0.4181	missense_var	851.00	275.00	Q/E	Caa/Gaa	tolerated(1)	benign(0)	0/1	0/1	0/1	0/1	0/1	0/1				
chr14_504350	MAP4K5	rs17780143	A:0.0535	missense_var	2217.00	633.00	T/M	aCg/aTg	deleterious(0)	probably_dam	0/1	0/1								
chr14_504439	MAP4K5	rs35768475	G:0.0054	missense_var	1738.00	473.00	K/N	aaG/aaC	deleterious(0.	probably_damaging(0.999)										
chr14_506655	SAV1	COSM3496194,COSM4051		missense_var	497.00	45.00	P/T	Cca/Aca	deleterious(0.	benign(0.034)	0/1	0/1								

chr14_50735	NIN	rs2295847	C:0.1933	missense_var	5991.00	1934.00	Q/E	Caa/Gaa	deleterious(0.	benign(0.054)	0/1	0/1	0/1	0/1	1/1	1/1			0/1	0/1
chr14_50752	NIN	rs61755995,C	A:0.0321	missense_var	5028.00	1613.00	R/C	Cgt/Tgt	deleterious(0.	possibly_damaging(0.855)					0/1	0/1				
chr14_50757	NIN	rs2073347	C:0.2356	missense_var	4150.00	1320.00	G/E	gGg/gAg	tolerated(1)	benign(0)	1/1	1/1	0/1	0/1	1/1	1/1			1/1	1/1
chr14_50757	NIN	rs12882191	T:0.2151	missense_var	3565.00	1125.00	Q/P	cAg/cCg	tolerated(1)	benign(0)	1/1	1/1	0/1	0/1	1/1	1/1			1/1	1/1
chr14_50979	TRIM9	rs2275462,C	T:0.4101	missense_var	3081.00	653.00	L/F	ttA/ttC	tolerated(0.11	benign(0.077)	1/1	1/1	0/1	0/1			0/1	0/1	0/1	0/1
chr14_51022	TRIM9	rs61755582	T:0.0150	missense_var	2134.00	338.00	V/I	Gtc/Atc	tolerated(0.28	benign(0.184)	0/1	0/1								
chr14_52029	NID2	rs2273430	C:0.0811	missense_var	2279.00	760.00	G/V	gGg/gTg	tolerated(1)	benign(0)	1/1	1/1	1/1	1/1	1/1	1/1	1/1	1/1	1/1	1/1
chr14_52040	NID2	rs3742536	A:0.1420	missense_var	1966.00	656.00	S/P	Tca/Cca	tolerated(1)	benign(0)	0/1	0/1	1/1	1/1	1/1	1/1	1/1	1/1	0/1	0/1
chr14_52053	NID2	rs2101919	C:0.4289	missense_var	1358.00	453.00	G/D	gGt/gAt	tolerated(0.71	benign(0.001)	0/1	0/1	0/1	0/1	0/1	0/1	1/1	1/1		
chr14_52068	NID2	rs3920038	G:0.1957	missense_var	65.00	22.00	P/Q	cCg/cAg	tolerated(0.41	benign(0)	1/1	1/1	1/1	1/1	1/1	1/1	1/1	1/1	1/1	1/1
chr14_52267	PTGDR	rs572021133,	T:0.0002	missense_var	253.00	51.00	R/W	Cgg/Tgg	deleterious(0.	benign(0.438)	0/1	0/1								
chr14_52470	TXNDC16	rs7155490	T:0.2206	missense_var	1828.00	486.00	E/K	Gaa/Aaa	tolerated(0.21	benign(0.024)	0/1	0/1	0/1	0/1	0/1	0/1			0/1	0/1
chr14_52553	GPR137C	rs147785041	A:0.4199	missense_var	79.00	27.00	S/G	Agc/Ggc	tolerated(0.41	benign(0)							0/1		0/1	0/1
chr14_52553	GPR137C			missense_var	170.00	57.00	Y/F	tAc/tTc	deleterious(0.	possibly_damaging(0.828)										
chr14_52632	GPR137C	rs762063	A:0.4123	missense_var	742.00	248.00	V/I	Gtc/Atc	tolerated(0.22	benign(0.003)			0/1	0/1			0/1	0/1	0/1	0/1
chr14_53152	DDHD1	rs117525276	G:0.0723	missense_var	317.00	106.00	G/A	gGt/gCt	tolerated_low	benign(0.206)	0/1	0/1								
chr14_53950	BMP4	rs17563,CM0	G:0.3257	missense_var	849.00	152.00	V/A	gTg/gCg	tolerated(0.72	benign(0.007)					1/1	1/1			1/1	1/1
chr14_55138	LGALS3	rs4644,CM08	A:0.2933	missense_var	452.00	64.00	P/H	cCt/cAt	deleterious(0)	probably_damaging(0.997)					1/1	1/1				
chr14_55138	LGALS3	rs4652,CM11	A:0.4303	missense_var	553.00	98.00	T/P	Acc/Ccc	tolerated(1)	benign(0)			0/1	0/1	1/1	1/1			0/1	0/1
chr14_55169	DLGAP5	rs17128275	A:0.1176	missense_var	1623.00	469.00	T/I	aCa/aTa	deleterious(0.	probably_damaging(0.942)			0/1	0/1					0/1	0/1
chr14_55177	DLGAP5	rs8010791	G:0.1176	missense_var	1189.00	324.00	Q/H	caA/caC	deleterious(0.	probably_damaging(0.958)			0/1	0/1					0/1	0/1
chr14_55188	DLGAP5	rs2274271	T:0.3622	missense_var	423.00	69.00	G/E	gGg/gAg	tolerated(1)	benign(0)			0/1	0/1					0/1	0/1
chr14_55351	FBXO34	rs1045002,C	A:0.3317	missense_var	1654.00	470.00	I/N	aTt/aAt	tolerated(0.67	benign(0.007)					1/1	1/1				
chr14_55351	FBXO34	rs3742569	C:0.3978	missense_var	1843.00	533.00	L/P	cTg/cCg	tolerated(0.28	benign(0)					1/1	1/1				
chr14_55440	TBPL2	rs8019270	C:0.3153	missense_var	163.00	31.00	R/P	cGg/cCg	tolerated_low	benign(0)	1/1	1/1	1/1	1/1			1/1	1/1	1/1	1/1
chr14_55617	KTN1	rs137964512	G:0.0018	missense_var	762.00	232.00	T/A	Act/Gct	tolerated(0.18	benign(0.003)										
chr14_56633	TMEM260	rs1041316,rs	G:0.2959	missense_var	1816.00	565.00	S/N	aGt/aAt	tolerated(1)	benign(0)	1/1	1/1		0/1	1/1	1/1	1/1		0/1	0/1
chr14_57471	C14orf105	rs1152530	T:0.1064	missense_var	866.00	275.00	Y/C	tAc/tGc	tolerated(0.09	benign(0.003)	1/1	1/1	1/1	1/1	1/1	1/1	1/1		1/1	1/1
chr14_58096	C14orf37	rs2273442	C:0.4888	missense_var	2032.00	613.00	Q/E	Caa/Gaa	tolerated(1)	benign(0)					0/1	0/1	1/1	1/1	0/1	0/1
chr14_58133	C14orf37	rs147981947	A:0.0008	missense_var	1680.00	495.00	E/D	gaG/gaT	deleterious(0.	possibly_damaging(0.721)							0/1	0/1		
chr14_58138	C14orf37	rs61741199	A:0.0701	missense_var	1229.00	345.00	T/I	aCa/aTa	tolerated(0.14	benign(0.066)							0/1	0/1		
chr14_58139	C14orf37	rs3829765	A:0.3413	missense_var	482.00	96.00	T/I	aCc/aTc	tolerated(0.07	possibly_damaging(0.494)					0/1	0/1	0/1	0/1		
chr14_58270	PSMA3	rs745454675		missense_var	689.00	200.00	I/T	aTa/aCa	tolerated(0.57	benign(0.015)			0/1	0/1						
chr14_58364	ARID4A	rs1051858	G:0.3494	missense_var	2708.00	779.00	T/A	Aca/Gca	tolerated_low	benign(0)	0/1	0/1	0/1	0/1					1/1	1/1
chr14_58470	KIAA0586	rs1748986	T:0.0317	missense_var	2931.00	896.00	L/P	cTt/cCt	tolerated(0.22	benign(0)	1/1	1/1	1/1	1/1	1/1	1/1	1/1	1/1	1/1	1/1
chr14_58487	KIAA0586	rs1617510	C:0.0260	missense_var	3569.00	1109.00	P/A	Cct/Gct	tolerated(1)	benign(0.001)		1/1		1/1	1/1		1/1			
chr14_58492	KIAA0586	rs3783697	G:0.0236	missense_var	4323.00	1360.00	D/G	gAt/gGt	deleterious(0)	possibly_damaging(0.453)									0/1	0/1
chr14_58543	KIAA0586	rs28535751	C:0.4531	missense_var	4947.00	1568.00	L/P	cTg/cCg	tolerated_low	benign(0)										
chr14_58646	DACT1	rs17832998	T:0.2696	missense_var	1415.00	464.00	A/V	gCt/gTt	tolerated(0.21	benign(0.02)			0/1	0/1						
chr14_58646	DACT1	rs698025	A:0.2702	missense_var	2113.00	697.00	G/S	Ggt/Agt	tolerated(0.43	benign(0)	0/1	0/1					1/1	1/1	0/1	0/1
chr14_59464	GPR135	rs76859844	T:0.0052	missense_var	688.00	191.00	S/N	aGc/aAc	tolerated(1)	benign(0.014)			0/1	0/1						
chr14_59464	GPR135	rs3742646	A:0.0319	missense_var	364.00	83.00	A/V	gCg/gTg	tolerated(0.1)	benign(0)										
chr14_59473	L3HYPDH	rs8660	T:0.4816	missense_var	1135.00	341.00	I/V	Ata/Gta	tolerated(1)	benign(0)					0/1	0/1	0/1	0/1	0/1	0/1
chr14_59473	L3HYPDH	rs1046701	A:0.0319	missense_var	1058.00	315.00	A/V	gCg/gTg	tolerated(0.34	benign(0.001)										
chr14_59521	CCDC175	rs12887189	T:0.2776	missense_var	2122.00	689.00	S/N	aGc/aAc	tolerated(0.21	benign(0.413)					0/1	0/1	0/1	0/1	0/1	0/1
chr14_59538	CCDC175	rs4261431	C:0.4683	missense_var	1576.00	507.00	G/E	gGa/gAa	tolerated(1)	benign(0)	1/1	1/1	1/1	1/1	0/1	0/1	0/1	0/1	0/1	0/1
chr14_59538	CCDC175	rs182831352	T:0.0070	missense_var	1507.00	484.00	I/N	aTt/aAt	deleterious(0)	probably_dam	0/1	0/1								
chr14_59561	CCDC175	rs112952088	T:0.0224	missense_var	939.00	295.00	E/K	Gaa/Aaa	tolerated(0.07	possibly_damaging(0.506)										
chr14_60027	LRRC9	rs574543223	T:0.0002	missense_var	3979.00	1259.00	R/C	Cgc/Tgc	deleterious(0)	probably_dam	0/1	0/1								
chr14_60107	PCNX4	rs78941281	A:0.0150	missense_var	624.00	24.00	V/I	Gtt/Att	tolerated(0.42	benign(0.019)			0/1	0/1						
chr14_60107	PCNX4	rs2296700	G:0.0178	missense_var	646.00	31.00	K/R	aAa/aGa	tolerated(0.31	benign(0.007)										
chr14_60115	PCNX4	rs150688	G:0.3486	missense_var	1785.00	411.00	V/I	Gta/Ata	tolerated(0.17	benign(0.001)	1/1	1/1	1/1	1/1	0/1	0/1	1/1	1/1	0/1	0/1
chr14_60118	PCNX4	rs308998	G:0.3411	missense_var	2217.00	555.00	K/E	Aaa/Gaa	tolerated(0.07	benign(0.011)					0/1	0/1			0/1	0/1
chr14_60124	PCNX4	rs3742642,C	T:0.0485	missense_var	2864.00	770.00	Q/H	caA/caT	tolerated(0.32	benign(0.001)					0/1	0/1			0/1	0/1
chr14_60125	PCNX4	rs167437	A:0.3488	missense_var	3552.00	1000.00	G/S	Ggt/Agt	tolerated(1)	benign(0.001)					0/1	0/1			0/1	0/1
chr14_60437	C14orf39	rs1254319	A:0.4669	missense_var	1730.00	524.00	L/F	Ctt/Ttt	tolerated(1)	benign(0)	0/1	0/1	0/1	0/1	0/1	0/1	0/1	0/1	1/1	1/1
chr14_60457	C14orf39	rs11625921	C:0.0948	missense_var	1370.00	404.00	V/I	Gta/Ata	tolerated(0.21	benign(0)	1/1	1/1	0/1	0/1	1/1	1/1		1/1	1/1	1/1
chr14_60466	C14orf39	rs12586711	A:0.2155	missense_var	1077.00	306.00	A/V	gCg/gTg	tolerated(1)	benign(0)	0/1	0/1			0/1	0/1	0/1	0/1	1/1	1/1
chr14_60509	SIX6	rs33912345	A:0.3415	missense_var	869.00	141.00	H/N	Cac/Aac	tolerated(0.56	benign(0.001)	0/1	0/1	0/1	0/1	0/1	0/1	0/1	0/1		
chr14_60648	SIX1			missense_var	604.00	111.00	V/L	Gtg/Ctg	tolerated(0.32	benign(0.145)										

chr14_60713	SIX4	rs3742636	T:0.3548	missense_var	1874.00	605.00	H/P	cAt/cCt	deleterious_lo	benign(0.367)	1/1	1/1			0/1	0/1	1/1	1/1	1/1	1/1
chr14_61046	SLC38A6	rs117560154	C:0.0054	missense_var	1006.00	281.00	M/T	aTg/aCg	deleterious(0.	probably_damaging(0.998)					0/1	0/1				
chr14_61050	SLC38A6	rs74825213	A:0.0072	missense_var	1116.00	318.00	G/S	Ggt/Agt	tolerated(0.42	benign(0.136)					0/1	0/1				
chr14_61280	TMEM30B	rs117362093,	A:0.0046	missense_var	1736.00	348.00	D/V	gAc/gTc	deleterious(0.	benign(0.14)									0/1	0/1
chr14_61280	TMEM30B	rs137950125	A:0.0142	missense_var	965.00	91.00	P/L	cCc/cTc	tolerated(0.12	benign(0.003)										
chr14_61740	HIF1A	rs11549465,C	T:0.0731	missense_var	2045.00	606.00	P/S	Cca/Tca	tolerated(0.07	benign(0.338)			0/1	0/1						
chr14_61762	SNAPC1	rs74810099	G:0.0497	missense_var	211.00	36.00	M/R	aTg/aGg	tolerated(0.17	benign(0.052)	0/1	0/1								
chr14_61996	SYT16	rs8019076	T:0.2077	missense_var	226.00	10.00	V/A	gTt/gCt	tolerated_low_	benign(0)	1/1		0/1	0/1	1/1	1/1	0/1			
chr14_61996	SYT16	rs17099370,C	T:0.2412	missense_var	589.00	131.00	R/L	cGc/cTc	tolerated_low_	benign(0)	0/1	0/1			1/1	1/1	0/1	0/1	1/1	1/1
chr14_62708	KCNH5	rs4902176	T:0.2212	missense_var	2502.00	745.00	A/T	Gcc/Acc	tolerated_low_	benign(0.003)							0/1	0/1	0/1	0/1
chr14_63312	GPHB5	rs118004333	A:0.0128	missense_var	427.00	124.00	T/S	Acc/Tcc	tolerated(0.26	benign(0.321)			0/1	0/1						
chr14_63981	SYNE2	rs9944035	C:0.1320	missense_var	1933.00	574.00	I/T	aTa/aCa	deleterious(0.	possibly_damaging(0.625)					0/1			0/1		
chr14_64021	SYNE2	rs189676187	G:0.0008	missense_var	5367.00	1719.00	M/V	Atg/Gtg	deleterious(0.	benign(0.038)							0/1	0/1		
chr14_64024	SYNE2	rs4902264	T:0.3211	missense_var	6118.00	1969.00	M/T	aTg/aCg	tolerated(0.6)	benign(0)	0/1	0/1	0/1	0/1	0/1	0/1	0/1	0/1	1/1	1/1
chr14_64030	SYNE2	rs4027402	C:0.3281	missense_var	7063.00	2284.00	A/V	gCg/gTg	deleterious(0.	benign(0.02)	0/1	0/1	0/1	0/1	0/1	0/1			1/1	1/1
chr14_64031	SYNE2	rs34625768	A:0.0491	missense_var	7252.00	2347.00	A/E	gCa/gAa	tolerated(1)	benign(0)			0/1	0/1						
chr14_64031	SYNE2	rs7157465	G:0.0491	missense_var	7287.00	2359.00	S/G	Agc/Ggc	deleterious(0.	benign(0.003)			0/1	0/1						
chr14_64031	SYNE2	rs4027404	G:0.2975	missense_var	7288.00	2359.00	S/N	aGc/aAc	tolerated(0.23	benign(0.012)	0/1	0/1	0/1	0/1	0/1	0/1	0/1	0/1	1/1	1/1
chr14_64031	SYNE2	rs111902869	C:0.0088	missense_var	7336.00	2375.00	L/P	cTg/cCg	tolerated(0.2)	benign(0.01)			0/1	0/1						
chr14_64031	SYNE2	rs4027405	G:0.1627	missense_var	7395.00	2395.00	A/T	Gca/Aca	tolerated(0.11	benign(0.01)	1/1	1/1	1/1	1/1	0/1	0/1	0/1	0/1	1/1	1/1
chr14_64052	SYNE2	rs1890908	A:0.1382	missense_var	8616.00	2802.00	S/G	Agt/Ggt	tolerated(0.17	benign(0.04)	1/1	1/1	1/1	1/1	0/1	0/1	0/1	0/1	1/1	1/1
chr14_64052	SYNE2	rs3829767	A:0.3349	missense_var	9036.00	2942.00	I/V	Ata/Gta	tolerated(0.14	benign(0.005)	0/1	0/1	0/1	0/1	0/1	0/1	0/1	0/1	1/1	1/1
chr14_64055	SYNE2	rs8010911	G:0.3187	missense_var	9969.00	3253.00	D/H	Gat/Cat	deleterious(0)	benign(0.445)	0/1	0/1	0/1	0/1	0/1	0/1		0/1		
chr14_64056	SYNE2	rs8010699	A:0.3177	missense_var	10138.00	3309.00	H/R	cAc/cGc	deleterious(0.	benign(0.043)	1/1	1/1	0/1	0/1	0/1	0/1	0/1	0/1	1/1	1/1
chr14_64062	SYNE2	rs36039501	A:0.0156	missense_var	10408.00	3399.00	R/H	cGc/cAc	tolerated(0.68	benign(0.003)										
chr14_64065	SYNE2	rs35920722	G:0.0174	missense_var	10481.00	3423.00	I/M	atC/atG	tolerated(0.08	benign(0.159)										
chr14_64065	SYNE2	rs144108695	A:0.0010	missense_var	10525.00	3438.00	C/Y	tGt/tAt	tolerated(0.33	possibly_dam	0/1	0/1								
chr14_64070	SYNE2	rs35203186	C:0.0393	missense_var	10779.00	3523.00	K/Q	Aag/Cag	tolerated(0.62	benign(0.005)					0/1	0/1				
chr14_64091	SYNE2	rs76576553	G:0.0164	missense_var	12147.00	3979.00	L/V	Cta/Gta	tolerated(0.26	benign(0.055)					0/1	0/1				
chr14_64091	SYNE2	rs10137972	C:0.0925	missense_var	12156.00	3982.00	N/H	Aat/Cat	tolerated(0.3)	possibly_dam	0/1	0/1			0/1	0/1				
chr14_64093	SYNE2	rs2792205	C:0.1236	missense_var	12213.00	4001.00	W/R	Tgg/Cgg	deleterious(0.	benign(0)					0/1	0/1			0/1	0/1
chr14_64093	SYNE2	rs2781377	A:0.1224	stop_gained	12214.00	4001.00	W/*	tGg/tAg							0/1	0/1			0/1	0/1
chr14_64093	SYNE2	rs75665958	C:0.0102	missense_var	12255.00	4015.00	Y/H	Tat/Cat	tolerated(0.39	benign(0.001)			0/1	0/1						
chr14_64137	SYNE2	rs17766354	G:0.0284	missense_var	14946.00	4912.00	P/A	Cct/Gct	tolerated(0.1)	possibly_dam	0/1	0/1								
chr14_64146	SYNE2	rs10151658	C:0.3682	missense_var	15768.00	5186.00	L/M	Ctg/Atg	tolerated(0.7)	benign(0.012)	1/1	1/1	1/1	1/1			0/1	0/1	1/1	1/1
chr14_64209	SYNE2	rs2275014	T:0.0004	missense_var	18676.00	6155.00	A/V	gCt/gTt	deleterious(0)	possibly_damaging(0.592)			0/1	0/1						
chr14_64211	SYNE2			missense_var	18969.00	6253.00	T/A	Acc/Gcc	tolerated(0.17	probably_damaging(0.956)										
chr14_64415	MTHFD1	rs1950902,CM	A:0.1765	missense_var	479.00	134.00	K/R	aAa/aGa	tolerated(1)	benign(0.017)	0/1	0/1	1/1	1/1	0/1	0/1	1/1	1/1	0/1	0/1
chr14_64442	MTHFD1	rs2236225,CM	A:0.3419	missense_var	2036.00	653.00	R/Q	cGg/cAg	tolerated(0.08	benign(0.015)							1/1	1/1	0/1	0/1
chr14_64469	AKAP5	rs1256149	C:0.0162	missense_var	986.00	203.00	T/I	aCa/aTa	tolerated(1)	benign(0)	1/1	1/1	1/1	1/1	1/1	1/1	1/1	1/1	1/1	1/1
chr14_64469	AKAP5	rs34433837,C	A:0.0094	missense_var	1318.00	314.00	E/K	Gaa/Aaa	tolerated(0.15	benign(0.009)										
chr14_64487	ZBTB25	rs61742301	G:0.0208	missense_var	458.00	89.00	I/T	aTt/aCt	tolerated(1)	benign(0)			0/1	0/1						
chr14_64564	PPP1R36	rs6573560	C:0.3822	missense_var	344.00	83.00	T/I	aCt/aTt	tolerated(0.86	benign(0)	1/1	1/1	0/1	0/1	0/1	0/1	0/1	0/1	1/1	1/1
chr14_64574	PPP1R36	rs200560352	C:0.0002	missense_var	637.00	181.00	V/L	Gta/Cta	tolerated(0.29	benign(0.003)									0/1	0/1
chr14_64775	SPTB	rs138126360	G:0.0028	missense_var	4842.00	1567.00	D/H	Gac/Cac	tolerated(0.12	benign(0.062)										
chr14_64782	SPTB	rs17245552	G:0.1016	missense_var	4365.00	1408.00	G/R	Ggc/Cgc	deleterious(0.	probably_dam	0/1	0/1	0/1	0/1						
chr14_64786	SPTB	rs77806	C:0.4694	missense_var	3594.00	1151.00	N/D	Aat/Gat	tolerated(0.62	benign(0.003)	0/1	0/1	0/1	0/1			0/1	0/1		
chr14_64796	SPTB	rs229587	T:0.4669	missense_var	1459.00	439.00	S/N	aGt/aAt	tolerated(0.35	benign(0.007)	1/1						0/1	0/1		
chr14_65616	FUT8	rs78083604	G:0.0036	missense_var	2184.00	153.00	L/V	Ctt/Gtt	tolerated(0.15	benign(0.007)					0/1	0/1				
chr14_65669	FUT8	rs35949016,C	A:0.1494	missense_var	2527.00	267.00	T/K	aCa/aAa	tolerated(0.99	benign(0.338)			0/1	0/1					0/1	0/1
chr14_67317	MPP5	rs531306493	G:0.0010	missense_var	2019.00	453.00	N/S	aAt/aGt	tolerated(0.59	benign(0.005)										
chr14_67557	PLEKHH1	rs3825723	T:0.1577	missense_var	470.00	113.00	Q/L	cAg/cTg	deleterious(0)	probably_dam	0/1	0/1	0/1	0/1	1/1	1/1			0/1	0/1
chr14_67562	PLEKHH1	rs2236235	A:0.1731	missense_var	1097.00	322.00	R/Q	cGg/cAg	tolerated(0.17	benign(0.003)			0/1	0/1	1/1	1/1	0/1	0/1	0/1	0/1
chr14_67569	PLEKHH1	rs3825725	G:0.2117	missense_var	1420.00	430.00	T/A	Aca/Gca	tolerated(0.08	benign(0.003)	0/1	0/1	0/1	0/1					0/1	0/1
chr14_67569	PLEKHH1	rs17104428	G:0.0387	missense_var	1444.00	438.00	M/V	Atg/Gtg	tolerated(0.95	benign(0)										
chr14_67574	PLEKHH1	rs61534804	A:0.1072	missense_var	2161.00	677.00	G/R	Ggg/Agg	tolerated(0.31	benign(0.018)	0/1	0/1			0/1	0/1	0/1	0/1		
chr14_67575	PLEKHH1	rs11158685	G:0.4786	missense_var	2336.00	735.00	H/R	cAc/cGc	tolerated(1)	benign(0)	0/1	0/1	0/1	0/1	0/1	0/1	1/1		0/1	0/1
chr14_67727	RDH12	rs17852293	A:0.1052	missense_var	806.00	161.00	R/Q	cGg/cAg	tolerated(0.3)	benign(0.037)					0/1	0/1				
chr14_67767	ZFYVE26	rs3742883	T:0.0968	missense_var	5811.00	1891.00	N/S	aAt/aGt	tolerated(1)	benign(0)	0/1	0/1	1/1	1/1	0/1	0/1	1/1	1/1	0/1	0/1
chr14_67782	ZFYVE26	rs2235967	T:0.1601	missense_var	4509.00	1457.00	C/Y	tGt/tAt	tolerated(0.1)	benign(0.003)			0/1	0/1			0/1	0/1		

chr14_67785	ZFYVE26	rs3742885	A:0.0745	missense_var	3447.00	1103.00	P/L	cCc/cTc	tolerated(0.22	benign(0.011)					0/1				0/1	0/1
chr14_67786	ZFYVE26	rs112787369	T:0.0272	missense_var	3257.00	1040.00	S/T	Tca/Aca	tolerated(0.28	benign(0.007)										
chr14_67798	ZFYVE26	rs77129887	C:0.0056	missense_var	2072.00	645.00	M/V	Atg/Gtg	tolerated(0.68	benign(0)			0/1	0/1						
chr14_67804	ZFYVE26	rs556064894		missense_var	1526.00	463.00	L/F	Ctc/Ttc	deleterious(0)	benign(0.12)										
chr14_68650	RAD51B	rs8023214	T:0.4714	missense_var	1078.00	351.00	C/R	Tgc/Cgc	tolerated_low_	benign(0)	0/1	0/1			0/1	0/1	0/1	0/1		
chr14_69237	EXD2	rs45557240	G:0.0054	missense_var	1756.00	479.00	K/E	Aag/Gag	tolerated(1)	benign(0)							0/1	0/1		
chr14_69237	EXD2	rs8007859	G:0.1957	missense_var	1875.00	518.00	Q/H	caG/caT	tolerated(0.18	benign(0)	1/1	1/1	1/1	1/1	1/1	1/1	0/1	0/1	1/1	1/1
chr14_69328	GALNT16	rs12879377	A:0.1755	missense_var	928.00	201.00	V/M	Gtg/Atg	tolerated(0.24	benign(0.027)			0/1	0/1			0/1	0/1		
chr14_69455	SLC39A9	rs2232059	C:0.0286	missense_var	1340.00	221.00	M/T	aTg/aCg	tolerated(0.19	benign(0.003)			0/1	0/1						
chr14_69573	CCDC177	rs4902734	C:0.2809	missense_var	783.00	153.00	E/D	gaG/gaC	tolerated(0.13	benign(0.152)	0/1	0/1								
chr14_70457	ADAM21	rs8010994		missense_var	543.00	95.00	D/E	gaC/gaG	tolerated(0.06	benign(0)	1/1	1/1	0/1	0/1	0/1	0/1	0/1	0/1	0/1	0/1
chr14_70457	ADAM21	rs3829452	G:0.3702	missense_var	608.00	117.00	A/G	gCa/gGa	tolerated(1)	benign(0)			0/1	0/1			0/1	0/1	1/1	1/1
chr14_70457	ADAM21			missense_var	739.00	161.00	I/V	Ata/Gta	tolerated(0.36	benign(0.001)			0/1	0/1			0/1	0/1	1/1	1/1
chr14_70458	ADAM21	rs114864695	C:0.2758	missense_var	997.00	247.00	K/Q	Aag/Cag	tolerated(0.8)	benign(0.01)			0/1	0/1			0/1	0/1	1/1	1/1
chr14_70642	TTC9	rs4902834	C:0.1583	missense_var	650.00	103.00	P/A	Ccg/Gcg	tolerated(0.52	benign(0)	1/1	1/1	1/1	1/1	0/1	0/1	1/1	1/1	1/1	1/1
chr14_70642	TTC9			missense_var	690.00	116.00	S/L	tCg/tTg	deleterious(0)	benign(0.012)	0/1	0/1								
chr14_70671	TTC9	rs45621632	T:0.0761	missense_var	984.00	214.00	C/F	tGc/tTc	tolerated(0.63	possibly_damaging(0.825)			1/1	1/1	0/1	0/1			0/1	0/1
chr14_70734	MAP3K9	rs143859920		missense_var	2278.00	639.00	L/V	Ctc/Gtc	tolerated(0.25	benign(0.132)	0/1	0/1								
chr14_70976	PCNX1	rs143154004	A:0.0022	missense_var	1066.00	207.00	R/Q	cGg/cAg	tolerated(0.15	benign(0)										
chr14_70977	PCNX1	rs141826688	G:0.0008	missense_var	1489.00	348.00	N/S	aAt/aGt	tolerated_low_	benign(0)			0/1	0/1						
chr14_71702	SIPA1L1	rs79937396	C:0.0104	missense_var	3902.00	1185.00	I/T	aTt/aCt	tolerated(0.41	benign(0)								0/1		
chr14_72892	DPF3	rs11158986	A:0.0573	missense_var	413.00	36.00	F/C	tTc/tGc	tolerated_low_	unknown(0)	1/1	1/1	1/1	1/1	1/1	1/1	1/1	1/1	1/1	1/1
chr14_72945	DCAF4	rs17856582	C:0.0214	missense_var	798.00	193.00	K/T	aAa/aCa	tolerated(0.17	benign(0.018)										
chr14_72946	DCAF4	rs755541492		missense_var	894.00	225.00	R/L	cGg/cTg	deleterious(0)	possibly_damaging(0.734)			0/1	0/1						
chr14_72955	DCAF4	rs3815460	G:0.0901	missense_var	1254.00	345.00	S/C	tCt/tGt	deleterious(0.	probably_damaging(0.994)			0/1	0/1	0/1	0/1	0/1	0/1		
chr14_73244	PAPLN	rs2280792	G:0.2380	missense_var	199.00	33.00	S/G	Agc/Ggc	tolerated(1)	benign(0)	0/1	0/1		1/1	0/1	0/1			0/1	0/1
chr14_73251	PAPLN	rs741842	A:0.0437	missense_var	673.00	191.00	A/T	Gct/Act	deleterious(0.	benign(0.143)										
chr14_73252	PAPLN	rs17126331,rs	C:0.0757	missense_var	1087.00	329.00	N/H	Aat/Cat	deleterious(0.	probably_dam	0/1	0/1								
chr14_73254	PAPLN	rs17126354	T:0.0321	missense_var	1403.00	434.00	A/V	gCa/gTa	tolerated(0.5)	benign(0.117)	0/1	0/1								
chr14_73259	PAPLN	rs17182244	G:0.0110	missense_var	1904.00	601.00	H/R	cAc/cGc	tolerated(0.71	benign(0)							0/1	0/1		
chr14_73260	PAPLN	rs177389	T:0.2794	missense_var	2099.00	666.00	M/R	aTg/aGg	tolerated(1)	benign(0)	1/1	1/1	0/1	0/1	0/1	0/1	0/1	0/1	1/1	1/1
chr14_73266	PAPLN			missense_var	3406.00	1102.00	V/I	Gtt/Att	tolerated(0.14	benign(0.212)										
chr14_73268	PAPLN	rs752392296		missense_var	3598.00	1166.00	R/W	Cgg/Tgg	deleterious(0)	possibly_damaging(0.857)							0/1	0/1		
chr14_73268	PAPLN	rs4903104	T:0.1150	missense_var	3623.00	1174.00	T/M	aCg/aTg	deleterious(0)	probably_damaging(1)							0/1	0/1	0/1	0/1
chr14_73276	NUMB	rs374579310,COSM213706		missense_var	2159.00	630.00	R/H	cGt/cAt	deleterious(0.	probably_damaging(0.945)							0/1	0/1		
chr14_73276	NUMB	rs17781919	T:0.0110	missense_var	2054.00	595.00	G/D	gGc/gAc	tolerated(0.37	benign(0.027)										
chr14_73537	ACOT1	rs199627073	T:0.0060	missense_var	730.00	141.00	A/V	gCg/gTg	tolerated(0.05	benign(0)										
chr14_73569	ACOT2	rs56069624	G:0.0355	missense_var	454.00	91.00	E/G	gAg/gGg	tolerated(0.62	benign(0.003)										
chr14_73569	ACOT2	rs774582968		missense_var	736.00	185.00	T/M	aCg/aTg	deleterious(0.	benign(0.315)										
chr14_73575	ACOT2			missense_var	1165.00	328.00	H/R	cAc/cGc	tolerated(0.3)	benign(0.003)				1/1			1/1	1/1		1/1
chr14_73575	ACOT2	rs199835203	G:0.0058	missense_var	1512.00	444.00	I/V	Att/Gtt	tolerated(1)	benign(0)		0/1								
chr14_73575	ACOT2	rs7494		missense_var	1606.00	475.00	H/R	cAc/cGc	tolerated(0.21	benign(0)	0/1	0/1	1/1		0/1	0/1	0/1		1/1	1/1
chr14_73591	ACOT4	rs139476628	T:0.0066	missense_var	259.00	2.00	S/L	tCa/tTa	deleterious(0)	benign(0.043)									0/1	0/1
chr14_73592	ACOT4	rs3742819	T:0.3970	missense_var	423.00	57.00	R/C	Cgc/Tgc	deleterious(0.	possibly_dam	0/1	0/1	0/1	0/1	0/1	0/1	0/1	0/1	1/1	1/1
chr14_73593	ACOT4	rs45522539	C:0.0351	missense_var	793.00	180.00	G/A	gGc/gCc	tolerated(0.06	benign(0.026)										
chr14_73737	ELMSAN1	rs17782124	A:0.1530	missense_var	2444.00	554.00	P/L	cCt/cTt	tolerated(0.32	benign(0)							0/1	0/1		
chr14_73938	FAM161B	rs28927675	C:0.4073	missense_var	1848.00	550.00	K/R	aAa/aGa	tolerated(0.33	benign(0.205)	0/1	0/1					0/1	0/1	1/1	1/1
chr14_73942	FAM161B	rs73301408	C:0.0022	missense_var	1394.00	399.00	S/A	Tct/Gct	tolerated(0.2)	benign(0.105)	0/1	0/1								
chr14_73961	COQ6	rs2074930	T:0.1496	missense_var	1056.00	339.00	D/V	gAt/gTt	tolerated(0.41	benign(0.012)	0/1	0/1							1/1	1/1
chr14_73961	COQ6	rs8500	A:0.3454	missense_var	1256.00	406.00	V/M	Gtg/Atg	tolerated(0.79	benign(0.006)	0/1	0/1					0/1	0/1		
chr14_74047	BBOF1	rs3784038	A:0.3682	missense_var	812.00	230.00	D/V	gAt/gTt	tolerated(1)	benign(0)	1/1		1/1	1/1	1/1				1/1	1/1
chr14_74057	BBOF1	rs3742809,CO	G:0.2416	missense_var	1609.00	496.00	K/E	Aaa/Gaa	tolerated(0.74	benign(0)	0/1	0/1							0/1	0/1
chr14_74292	ABCD4	rs3742801	T:0.2704	missense_var	1246.00	368.00	E/K	Gag/Aag	tolerated(0.31	benign(0)			0/1	0/1						
chr14_74292	ABCD4	rs4148077,CO	T:0.2704	missense_var	1054.00	304.00	A/T	Gca/Aca	tolerated(1)	benign(0.003)			0/1	0/1						
chr14_74409	SYNDIG1L	rs61734850	C:0.1040	missense_var	341.00	31.00	S/R	agC/agG	tolerated(0.13	benign(0.147)			0/1	0/1						
chr14_74509	LTBP2	rs61505039	T:0.0603	missense_var	3649.00	1088.00	G/S	Ggc/Agc	deleterious(0.	benign(0.342)	0/1	0/1								
chr14_74521	LTBP2	rs765072306		missense_var	3088.00	901.00	R/C	Cgc/Tgc	deleterious(0)	possibly_dam	0/1	0/1								
chr14_74585	LTBP2	rs149952751,	A:0.0004	missense_var	1187.00	267.00	S/L	tCg/tTg	tolerated(0.36	benign(0)							0/1	0/1		
chr14_74723	FCF1	rs41309250	C:0.0030	missense_var	374.00	107.00	M/T	aTg/aCg	deleterious(0.	possibly_damaging(0.491)							0/1			
chr14_74763	YLPM1	rs201738923	C:0.0002	missense_var	210.00	29.00	E/A	gAg/gCg	deleterious_lo	benign(0.37)							0/1	0/1		

chr14_747642	YLPM1	rs45599947	T:0.0268	missense_var	885.00	254.00	P/L	cCt/cTt	deleterious_lc	benign(0.01)										
chr14_747813	YLPM1	rs138920894	T:0.0138	missense_var	1479.00	452.00	Y/F	tAc/tTc	tolerated(0.4)	benign(0.005)	0/1	0/1								
chr14_749198	RPS6KL1	rs2286913	A:0.2979	missense_var	847.00	121.00	P/L	cCg/cTg	deleterious(0.	benign(0.03)			0/1	0/1	0/1	0/1			0/1	0/1
chr14_749214	RPS6KL1	rs7156590	T:0.3518	missense_var	547.00	21.00	R/Q	cGa/cAa	deleterious(0.	benign(0.054)	0/1	0/1	0/1	0/1	0/1	0/1			0/1	0/1
chr14_750468	MLH3	rs17102999,C	A:0.0188	missense_var	3041.00	942.00	T/I	aCa/aTa	tolerated(0.32	benign(0.023)	0/1	0/1								
chr14_750471	MLH3	rs175080	A:0.3638	missense_var	2747.00	844.00	P/L	cCt/cTt	tolerated(0.18	benign(0)							0/1	0/1		
chr14_750471	MLH3	rs175081	T:0.0096	missense_var	2692.00	826.00	N/D	Aac/Gac	tolerated(0.2)	benign(0)	1/1	1/1	1/1	1/1	1/1	1/1	1/1	1/1	1/1	1/1
chr14_750474	MLH3	rs28756990,C	A:0.0260	missense_var	2437.00	741.00	V/F	Gtc/Ttc	tolerated(0.44	possibly_dam	0/1	0/1								
chr14_750715	ZC2HC1C	rs3742778	G:0.4772	missense_var	1430.00	314.00	G/V	gGt/gTt	tolerated(0.19	benign(0.003)	0/1	0/1	1/1	1/1	1/1	1/1	0/1	0/1	1/1	1/1
chr14_751073	NEK9	rs10146482	C:0.4764	missense_var	1445.00	429.00	R/H	cGt/cAt	tolerated(0.32	benign(0.001)	0/1	0/1	1/1	1/1	1/1	1/1		0/1	1/1	1/1
chr14_754379	JDP2	rs3625	A:0.3936	missense_var	263.00	24.00	T/A	Aca/Gca	tolerated(1)	benign(0)		1/1	0/1	0/1	0/1	0/1	0/1	0/1	1/1	1/1
chr14_755790	FLVCR2	rs2287015	C:0.4738	missense_var	403.00	16.00	V/A	gTg/gCg	tolerated_low	benign(0)	0/1	0/1	1/1	1/1	1/1	1/1	0/1	0/1	0/1	0/1
chr14_756902	TTLL5	rs2303345	T:0.3944	missense_var	651.00	149.00	A/V	gCt/gTt	tolerated(0.09	benign(0.326)	0/1				0/1	0/1	0/1	0/1	0/1	0/1
chr14_759022	TTLL5	rs1133834	C:0.4255	missense_var	4005.00	1267.00	F/S	tTt/tCt	tolerated_low	benign(0)	1/1	1/1	0/1	0/1	0/1	0/1			1/1	1/1
chr14_760766	IFT43	rs17783366	A:0.1689	missense_var	285.00	94.00	D/N	Gat/Aat	tolerated(0.29	benign(0.001)										
chr14_764393	ESRRB	rs143477571	G:0.0054	missense_var	224.00	6.00	R/G	Agg/Ggg	deleterious_lc	probably_damaging(0.997)					0/1	0/1				
chr14_764983	ESRRB	rs146351534,	A:0.0052	missense_var	1445.00	413.00	V/I	Gtc/Atc	tolerated(0.58	benign(0.015)							0/1	0/1		
chr14_768034	ANGEL1	rs2075773	C:0.1290	missense_var	1657.00	515.00	F/C	tTc/tGc	deleterious(0)	probably_dam	0/1	0/1					0/1	0/1		
chr14_768079	ANGEL1	rs139731348	T:0.0004	missense_var	962.00	283.00	M/I	atG/atA	deleterious(0.	benign(0.012)									0/1	0/1
chr14_768283	LRRC74A	rs148747129	T:0.0066	missense_var	282.00	53.00	P/L	cCg/cTg	tolerated(0.28	benign(0.001)									0/1	0/1
chr14_768660	LRRC74A	rs7160583	C:0.2286	missense_var	1473.00	450.00	V/A	gTg/gCg	tolerated(1)	benign(0)	0/1	0/1					0/1	0/1		
chr14_772488	TMEM63C			missense_var	1966.00	608.00	S/G	Agc/Ggc	tolerated(0.05	benign(0.014)					0/1	0/1				
chr14_773268	GSTZ1	rs7975	A:0.3115	missense_var	379.00	32.00	E/K	Gag/Aag	deleterious(0.	benign(0.072)	0/1	0/1	0/1	0/1	1/1	1/1	0/1	0/1	0/1	0/1
chr14_773268	GSTZ1	rs7972,CM00	A:0.0306	missense_var	409.00	42.00	G/R	Ggg/Agg	deleterious(0)	benign(0.359)										
chr14_773279	GSTZ1	rs1046428	T:0.1470	missense_var	530.00	82.00	M/T	aTg/aCg	tolerated(1)	benign(0)	1/1	1/1	1/1	1/1	1/1	1/1	1/1	1/1	1/1	1/1
chr14_773770	TMED8	rs10141317	T:0.2496	missense_var	100.00	15.00	P/H	cCc/cAc	deleterious_lc	benign(0.397)	0/1	0/1								
chr14_773770	TMED8	rs3742737	T:0.2486	missense_var	69.00	5.00	Q/K	Cag/Aag	tolerated_low	benign(0.001)	0/1	0/1								
chr14_774847	ISM2	rs3742728	C:0.4103	missense_var	337.00	94.00	A/T	Gcc/Acc	tolerated_low	benign(0.007)	0/1	0/1	1/1	1/1	1/1	1/1	1/1	1/1	1/1	1/1
chr14_776740	ALKBH1	rs6494	A:0.1186	missense_var	986.00	324.00	M/L	Atg/Ttg	tolerated(0.48	benign(0)							0/1	0/1		
chr14_782437	NRXN3	rs11626446	A:0.1999	missense_var	665.00	203.00	G/D	gGt/gAt	tolerated(0.42	benign(0.087)	0/1	0/1								
chr14_802032	DIO2	rs225014,CM	C:0.4581	missense_var	410.00	128.00	T/A	Aca/Gca	tolerated(0.78	benign(0)									1/1	1/1
chr14_805049	CEP128	rs61744330,C	T:0.0631	missense_var	3551.00	1059.00	V/M	Gtg/Atg	tolerated(0.23	benign(0)										
chr14_807849	CEP128	rs327463	C:0.3323	missense_var	2571.00	732.00	H/R	cAt/cGt	tolerated(0.2)	possibly_dam	0/1	0/1	0/1	0/1			0/1	0/1		
chr14_809165	CEP128	rs7160694	A:0.0343	missense_var	423.00	16.00	R/L	cGa/cTa	deleterious(0.	benign(0.275)									0/1	0/1
chr14_811442	TSHR	rs1991517,CM	G:0.1032	missense_var	2503.00	727.00	E/D	gaG/gaC	tolerated(1)	benign(0)	1/1	1/1	1/1	1/1	1/1	1/1	0/1	0/1	1/1	1/1
chr14_812618	STON2	rs17111619	A:0.0371	missense_var	3108.00	899.00	V/L	Gtg/Ttg	tolerated_low	benign(0)										
chr14_812707	STON2	rs2241621,CM	C:0.4329	missense_var	2964.00	851.00	S/A	Tct/Gct	tolerated(0.13	probably_dam	1/1	1/1	0/1	0/1	1/1	1/1	0/1	0/1	1/1	1/1
chr14_812772	STON2	rs36017951	C:0.0371	missense_var	2503.00	697.00	N/S	aAt/aGt	tolerated(0.29	benign(0.015)			0/1	0/1					0/1	0/1
chr14_812773	STON2	rs34323725	T:0.0687	missense_var	2350.00	646.00	R/H	cGt/cAt	tolerated(0.91	benign(0.014)							0/1	0/1		
chr14_812783	STON2	rs3813535,CM	A:0.2550	missense_var	1332.00	307.00	S/P	Tca/Cca	tolerated(1)	benign(0)	1/1	1/1	1/1	1/1	1/1	1/1	1/1	1/1	1/1	1/1
chr14_879348	GALC	rs421262,CM	T:0.0593	missense_var	2028.00	641.00	T/A	Acc/Gcc	tolerated(1)	benign(0)	1/1	1/1	1/1	1/1	1/1	1/1	1/1	1/1	1/1	1/1
chr14_879415	GALC	rs398607	G:0.4475	missense_var	1792.00	562.00	I/T	aTa/aCa	deleterious(0)	benign(0.361)	0/1	0/1	1/1	1/1			0/1	0/1		
chr14_879656	GALC	rs74887188,C	C:0.0182	missense_var	1020.00	305.00	I/V	Atc/Gtc	deleterious(0.	benign(0.014)		0/1							0/1	0/1
chr14_879763	GALC	rs34362748	T:0.0795	missense_var	849.00	248.00	D/N	Gat/Aat	deleterious(0.	benign(0.029)										
chr14_879931	GALC	rs111887056,	G:0.0789	missense_var	168.00	21.00	A/P	Gcg/Ccg	tolerated_low	probably_damaging(0.955)										
chr14_881856	KCNK10	rs17762463	T:0.1022	missense_var	2000.00	517.00	A/T	Gct/Act	tolerated_low	benign(0.005)							1/1	1/1		
chr14_883858	SPATA7	rs4904448	A:0.1899	missense_var	293.00	2.00	D/N	Gat/Aat	tolerated(0.07	benign(0.385)	0/1	0/1								
chr14_883914	SPATA7			missense_var	344.00	19.00	P/A	Ccg/Gcg	tolerated(0.6)	possibly_damaging(0.606)										
chr14_883961	SPATA7	rs3179969	A:0.4075	missense_var	509.00	74.00	V/M	Gtg/Atg	tolerated(1)	benign(0)							0/1	0/1		
chr14_884723	PTPN21	rs2274736,CM	G:0.3958	missense_var	3092.00	936.00	V/A	gTg/gCg	tolerated(0.51	benign(0)					0/1	0/1	0/1	0/1		
chr14_884802	PTPN21	rs2401751,CM	A:0.3564	missense_var	1438.00	385.00	L/F	Ctc/Ttc	tolerated(0.25	benign(0.03)					0/1	0/1	0/1	0/1		
chr14_887389	EML5	rs17188228	C:0.2460	missense_var	1054.00	269.00	I/V	Att/Gtt	tolerated(0.36	benign(0.207)					1/1	1/1	0/1	0/1	0/1	0/1
chr14_901846	KCNK13	rs3814848	A:0.1564	missense_var	1354.00	305.00	G/R	Gga/Aga	tolerated(0.76	benign(0)			0/1	0/1			0/1	0/1	0/1	0/1
chr14_902633	PSMC1			missense_var	325.00	74.00	M/I	atG/atC	tolerated(0.51	benign(0.049)										
chr14_902790	NRDE2	rs3737035	C:0.3243	missense_var	3586.00	1118.00	N/S	aAt/aGt	tolerated(0.16	benign(0.003)	0/1	0/1			1/1	1/1	0/1	0/1		
chr14_903041	NRDE2	rs150585386	C:0.0002	missense_var	1032.00	267.00	T/A	Acc/Gcc	tolerated(0.11	benign(0)							0/1	0/1		
chr14_909784	RPS6KA5			missense_var	464.00	97.00	H/R	cAt/cGt	tolerated(0.13	probably_damaging(0.999)										
chr14_911701	C14orf159	rs4900072	T:0.1671	missense_var	1133.00	148.00	A/V	gCg/gTg	tolerated(1)	benign(0.001)	0/1	0/1								
chr14_911760	C14orf159	rs34302825	A:0.0250	missense_var	1415.00	242.00	S/N	aGc/aAc	tolerated(0.19	benign(0.037)										
chr14_912047	C14orf159	rs2295524	A:0.1845	missense_var	2209.00	507.00	D/N	Gat/Aat	deleterious(0.	possibly_damaging(0.731)			0/1	0/1	0/1	0/1			0/1	0/1

chr14_912727	CCDC88C	rs941920	A:0.1941	missense_var	6062.00	1992.00	L/P	cTc/cCc	tolerated(0.52	benign(0)	1/1	1/1	0/1	0/1	1/1	1/1	1/1	1/1		
chr14_912733	CCDC88C	rs201814255	T:0.0008	missense_var	5477.00	1797.00	R/H	cGc/cAc	deleterious(0.	possibly_damaging(0.862)									0/1	0/1
chr14_913071	CCDC88C	rs1970911	A:0.1663	missense_var	3170.00	1028.00	A/V	gCg/gTg	tolerated(0.59	benign(0)							0/1	0/1		
chr14_919747	TRIP11	rs1051340	T:0.2714	missense_var	5853.00	1827.00	G/S	Ggt/Agt	tolerated(0.07	benign(0.351)	0/1	0/1		1/1	0/1	0/1	0/1		1/1	1/1
chr14_919938	TRIP11	rs80200454	T:0.0351	missense_var	5460.00	1696.00	E/K	Gaa/Aaa	deleterious(0.	benign(0.006)	0/1	0/1			0/1	0/1				
chr14_919994	TRIP11	rs35007347	T:0.0080	missense_var	5101.00	1576.00	R/H	cGt/cAt	tolerated(0.09	benign(0.299)										
chr14_920060	TRIP11	rs59635749	C:0.0351	missense_var	2278.00	635.00	S/C	tCt/tGt	deleterious(0.	possibly_dam	0/1	0/1			0/1	0/1				
chr14_920824	ATXN3	rs1048755	T:0.3251	missense_var	692.00	212.00	V/M	Gtg/Atg	tolerated(0.13	benign(0.036)	0/1	0/1	1/1	1/1	0/1	0/1	0/1	0/1	1/1	1/1
chr14_924564	SLC24A4	rs62623445	A:0.0188	missense_var	1311.00	362.00	S/N	aGc/aAc	tolerated(0.18	benign(0.006)							0/1	0/1		
chr14_924921	SLC24A4	rs45587635	C:0.0673	missense_var	1880.00	552.00	K/Q	Aag/Cag	tolerated(0.31	benign(0.051)										1/1
chr14_926516	RIN3	rs3829947	G:0.4425	missense_var	803.00	215.00	H/R	cAt/cGt	tolerated(0.27	benign(0)	1/1	1/1	0/1	0/1	0/1	0/1				
chr14_926523	RIN3	rs3742717	T:0.2925	missense_var	1433.00	425.00	T/M	aCg/aTg	tolerated(0.13	benign(0.003)	0/1	0/1	0/1	0/1	0/1	0/1	0/1	0/1	0/1	0/1
chr14_927327	LGMN	rs2236264,C(	T:0.2338	missense_var	390.00	18.00	V/I	Gtt/Att	tolerated(0.27	benign(0)									0/1	0/1
chr14_927976	GOLGA5	rs17128572,C	G:0.1194	missense_var	456.00	67.00	A/G	gCt/gGt	tolerated(0.07	possibly_dam	0/1	0/1	0/1	0/1					0/1	0/1
chr14_928103	GOLGA5	rs1040835	T:0.1404	missense_var	1304.00	350.00	F/L	Ttt/Ctt	tolerated(1)	benign(0)	1/1	1/1	1/1	1/1	1/1	1/1	0/1		1/1	1/1
chr14_928333	GOLGA5	rs150295315	A:0.0010	missense_var	2195.00	647.00	G/S	Ggt/Agt	tolerated(0.37	benign(0.011)			0/1	0/1						
chr14_944465	SERPINA11	rs1885137	G:0.2604	missense_var	754.00	230.00	E/A	gAa/gCa	tolerated(0.06	benign(0.098)			0/1	0/1			0/1	0/1		
chr14_944647	SERPINA9	rs11628722	A:0.3239	missense_var	1118.00	348.00	V/A	gTc/gCc	tolerated(1)	benign(0.003)			0/1	0/1			1/1	1/1		
chr14_944671	SERPINA9	rs28618118	A:0.3223	missense_var	1004.00	310.00	R/I	aGa/aTa	tolerated(0.36	benign(0.003)										
chr14_944673	SERPINA9	rs28583900	T:0.3225	missense_var	837.00	254.00	H/Q	caT/caA	tolerated(0.36	benign(0)										
chr14_944673	SERPINA9	rs17090921	A:0.3225	missense_var	782.00	236.00	P/L	cCt/cTt	tolerated(0.09	benign(0.072)										
chr14_944695	SERPINA9	rs12879019	C:0.1783	missense_var	406.00	111.00	Q/E	Cag/Gag	tolerated(0.11	possibly_damaging(0.73)			0/1	0/1	0/1	0/1				
chr14_944697	SERPINA9	rs4905204,C(	A:0.0727	missense_var	200.00	42.00	A/V	gCc/gTc	tolerated(0.4)	benign(0)							0/1	0/1		
chr14_944697	SERPINA9	rs45438398	A:0.0585	missense_var	197.00	41.00	P/L	cCg/cTg	deleterious(0.	benign(0.01)										
chr14_945874	SERPINA5	rs141185188		missense_var	340.00	35.00	H/Q	caT/caG	tolerated(0.49	benign(0.062)										
chr14_945875	SERPINA5	rs6118	T:0.2760	missense_var	399.00	55.00	A/V	gCc/gTc	tolerated(0.14	benign(0.13)					0/1	0/1			0/1	0/1
chr14_945875	SERPINA5	rs6115	G:0.3788	missense_var	426.00	64.00	S/N	aGc/aAc	tolerated(1)	benign(0)			0/1	0/1	0/1	0/1	1/1	1/1	1/1	1/1
chr14_945876	SERPINA5	rs6119	G:0.2690	missense_var	548.00	105.00	K/E	Aag/Gag	tolerated(1)	benign(0)					0/1	0/1			0/1	0/1
chr14_945901	SERPINA5	rs140138746,	C:0.0008	missense_var	917.00	228.00	E/Q	Gag/Cag	deleterious(0.	benign(0.366)										
chr14_946144	SERPINA3	rs4934,CM95	A:0.3994	missense_var	1173.00	9.00	A/T	Gct/Act	tolerated(0.22	benign(0.019)	0/1	0/1			0/1	0/1	0/1	0/1	0/1	0/1
chr14_954179	SYNE3	rs12434757	G:0.4499	missense_var	2783.00	923.00	A/V	gCg/gTg	tolerated(1)	benign(0.003)	0/1			1/1	1/1	1/1	1/1			1/1
chr14_954399	SYNE3	rs9671369	A:0.4125	missense_var	2018.00	668.00	T/M	aCg/aTg	tolerated(0.13	probably_dam	0/1	0/1	0/1	0/1	0/1	0/1				
chr14_955440	GLRX5	rs11628901	A:0.0831	missense_var	933.00	146.00	A/T	Gcc/Acc	deleterious(0.	benign(0.065)							0/1			
chr14_956908	TCL1B	rs1064017	A:0.2622	missense_var	328.00	93.00	G/R	Ggg/Agg	deleterious(0.	benign(0.347)										
chr14_957123	TCL1A	rs17093294	T:0.0435	missense_var	296.00	56.00	V/I	Gtc/Atc	tolerated(0.33	benign(0)										
chr14_957139	TCL1A			missense_var	221.00	31.00	Q/E	Cag/Gag	tolerated(0.07	benign(0.244)										
chr14_963056	ATG2B	rs2289622	A:0.1064	missense_var	5594.00	1567.00	I/T	aTa/aCa	tolerated(0.99	benign(0)	0/1				0/1	0/1	1/1			
chr14_963111	ATG2B	rs3759601	C:0.2498	missense_var	5041.00	1383.00	Q/E	Caa/Gaa	tolerated(0.9)	benign(0.003)	0/1	0/1	0/1	0/1			0/1			
chr14_963155	ATG2B	rs9323945	T:0.0935	missense_var	4264.00	1124.00	N/D	Aat/Gat	tolerated(1)	benign(0)	0/1	0/1	1/1	1/1	0/1	0/1	1/1	1/1	1/1	1/1
chr14_963471	ATG2B	rs78100888	T:0.0030	missense_var	1214.00	107.00	R/H	cGc/cAc	deleterious(0.	probably_dam	0/1	0/1								
chr14_964047	AK7	rs2275554	A:0.2135	missense_var	349.00	102.00	R/Q	cGa/cAa	tolerated(0.11	benign(0.037)					0/1	0/1	0/1	0/1	0/1	
chr14_964564	AK7	rs2369679	C:0.1154	missense_var	1211.00	389.00	N/K	aaC/aaG	tolerated(1)	benign(0)	0/1	0/1	1/1	1/1	1/1	1/1	1/1	1/1	1/1	1/1
chr14_987161	C14orf177	rs17097718	T:0.0863	missense_var	426.00	3.00	R/W	Cgg/Tgg	tolerated_low	benign(0.157)	0/1	0/1			0/1	0/1				
chr14_987162	C14orf177	rs8009159	A:0.0266	missense_var	450.00	11.00	E/K	Gag/Aag	deleterious_lo	benign(0.007)			0/1	0/1	0/1	0/1				
chr14_995171	CCDC85C	rs58668399	A:0.0066	missense_var	1074.00	336.00	S/L	tCg/tTg	tolerated(0.09	benign(0.213)	0/1	0/1								
chr14_996753	HHIPL1	rs7158073	T:0.4838	missense_var	2173.00	692.00	V/A	gTg/gCg	tolerated_low	benign(0.003)			0/1	0/1	0/1	0/1	0/1	0/1	0/1	0/1
chr14_999093	EML1	rs34198557	T:0.2502	missense_var	1321.00	396.00	A/V	gCg/gTg	tolerated(0.92	benign(0.023)	0/1	0/1	1/1	1/1						
chr14_999146	EML1	rs2250718	T:0.3359	missense_var	1857.00	575.00	S/P	Tct/Cct	tolerated(0.82	benign(0)	0/1	0/1	1/1	1/1						

Supplementary Table S2. Numerical aberrations according to CGH analysis.

	AT-1<br>BCP-ALL at 1st relapse	AT-2<br>BCP-ALL at 2nd relapse	NALM-20<br>BCP-ALL at diagnosis	NALM-21<br>BCP-ALL at relapse	NALM-27<br>BCP-ALL at diagnosis	NALM-30<br>BCP-ALL at relapse	PC-53<br>BCP-ALL at 3rd relapse	PC-53A<br>PCP-ALL at final stage	YCUB-4<br>BCP-ALL at diagnosis	YCUB-4R<br>BCP-ALL at relapse
chr1	del(1p36.31-36.11)	del(1p36.33), del(1p36.31-36.11)	no	no	no	no	amp, del, del	amp, del	del(1p36), amp(1p36)	amp(1p36), LOH(1p), amp(1q)
chr2	del(q37.1-37.1)	no	del	del	no	no	no	no	amp	amp
chr3	no	no	no	no	del	del	del(p24), del(p14)	del(q12/13)	no	no
chr4	no	no	no	no	no	no	no	del	amp, del	amp, del
chr5	no	no	no	no	no	no	del(q31.3), del(q32)	del(q31.3), del(q11.2)	no	no
chr6	del	del	no	no	del	del	no	no	del(q27)	del(q27)
chr7	del	del	del	del	del	del	del	del	no	no
chr8	no	no	amp(8q), partial del(p21.3)	amp(8q), amp(8p), del(8p)	amp 8q	amp 8q	chr8 3n, (q13.3-q24.3) 4n	chr 8 3n	no	chr 8 3n
chr9	LOH on p;del(p21.3)	LOH on p; del(p21.3)	del (p21.3)	del (p21.3)	del(p21.3), del(q34.11)	del(p21.3), del(q34.11)	del(p21.3), del(q33.1)	del(p21.3)	p 1n; del(p21.3) (0n), del(p23)	p 2n LOH; del(p21.3) (0n), del(p23)
chr10	del	del	no	no	del(q22.1-q22.2)	del(q22.1-q22.2)	del	del	no	no
chr11	no	no	del	del	del	del	del	del	no	no
chr12	del	del	del	del	del	del	no	no	no	no
chr13	del	del	del	del	no	no	del	del, amp(q12.3-q34)	del(q22.3)	del(q22.3), chr 13 3n
chr14	del	del	del(q32.33)	del(q32.33)	del	del	amp	amp	del, amp	del, amp
chr15	no	no	del	del	no	no	del	amp(q14-q26.3)	no	no
chr16	amp	amp	no	no	no	no	no	no	no	no
chr17	no	amp	no	no	no	no	del	del	no	no
chr18	no	no	no	no	no	no	no	no	no, partial LOH	chr 18 3n, partial LOH
chr19	no	no	no	no	del	del	no	no	no	no
chr20	no	no	del	del	del	del	no	no	no	chr 20 3n
chr21	chr 21 4n LOH	chr 21 4n LOH	del	del	no	no	no	no	no	no
chr22	amp(q11.22) and del(q11.22)	amp(q11.22) and del(q11.22)	no	no	amp(q11.22) and del(q11.22)	amp(q11.22) and del(q11.22)	amp(q11.22) and del(q11.22)	amp(q11.22) and del(q11.22)	no	chr 22 3n

Green: numerical aberrations common to both sisters, red: aberration only in later sister cell line; purple: aberration in earlier sister only; del: deleted; amp: amplified; LOH: loss of heterozygosity.

Supplementary Table S3. Sequences of PCR primers.

Gene	Direction	RefSeq	Primer
BCL9 exon 8	forward	NM_004326.4	5´- CTG GAT GGA CCT CTT CTC C - 3´
BCL9 exon 9	reverse	NM_004326.4	5´- TGG CTC TGG AGG CAT GGT A - 3´
ETV6 exon 5	forward	NM_001987.5	5´- TCG GGA AGA CCT GGC TTA C - 3´
ETV6 exon 6	reverse	NM_001987.5	5´- CAG TCC GTT GGG ATC CAC T - 3´
MEF2D exon 5	forward	NM_005920.4	5´ - CAT CCC TCA CGG ACC CG - 3´
MEF2D exon 6	reverse	NM_005920.4	5´- GGC TCC GTT AGC ACT GTT C - 3´
NSMAF1 exon 1	forward	NM_003580	5´- CTC CAT GGC GTT TAT CCG G - 3´
NUCKS exon 3	reverse	NM_022731.5	5´- AGG GCC CGA ATC TCT TCC A - 3´
PAX5 intron 1/2	forward	NM_016734.3	5´- GTG ACT GGT TCT AAC TAC CCT - 3´
PAX5 intron 2/3	reverse	NM_016734.3	5´- AAG CCT CGA GCT ACT GCC T - 3´
RUNX1 exon 3	forward	NM_001754.5	5´- TGC ATA CTT GGA ATG AAT CCT TC - 3´
RUNX1 exon 4	reverse	NM_001754.5	5´- CAA CGC CTC GCT CAT CTT G - 3´

PCR conditions: 59°C, 36 cycles.
