# Supplementary material for: Molecular Genetics of Pre-B Acute Lymphoblastic Leukemia Sister Cell Lines during Disease Progression
Source: Curr Issues Mol Biol. 2021 Nov 30;43(3):2147–56. doi: 10.3390/cimb43030149 (PMC8929001; doi:10.3390/cimb43030149)
Supplement: Supplementary file 1 [file cimb-43-00149-s001.zip › cimb-1467938-supplementary.pdf]

Supplementary Files

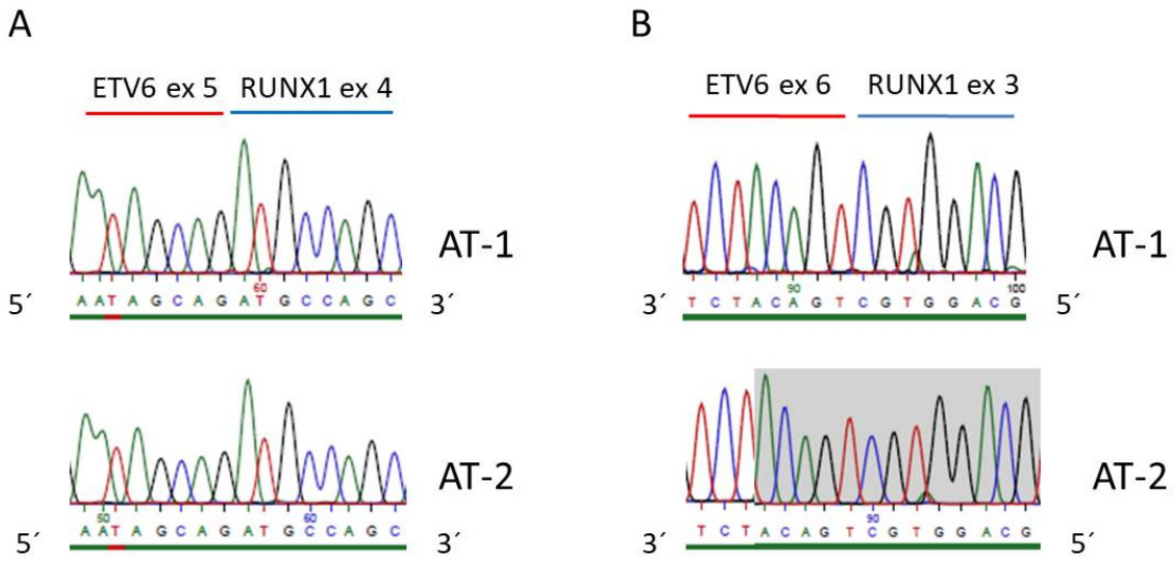

Figure S1. Fusion transcripts joining *ETV6* and *RUNX1*. Pre-B ALL cell lines AT-1 and AT-2 express A) *ETV6* exon 5 / *RUNX1* exon 4 and B) *RUNX1* exon 3 / ETV6 exon 6 fusion transcirpts. The *ETV6*/*RUNX1* PCR product was sequenced with the ETV6 exon 5 FRW primer, the *RUNX1*/*ETV6* PCR fusion transcript was sequenced with the ETV6 exon 6 reverse primer (Suppl. Table 3). The fusion transcripts are in frame.

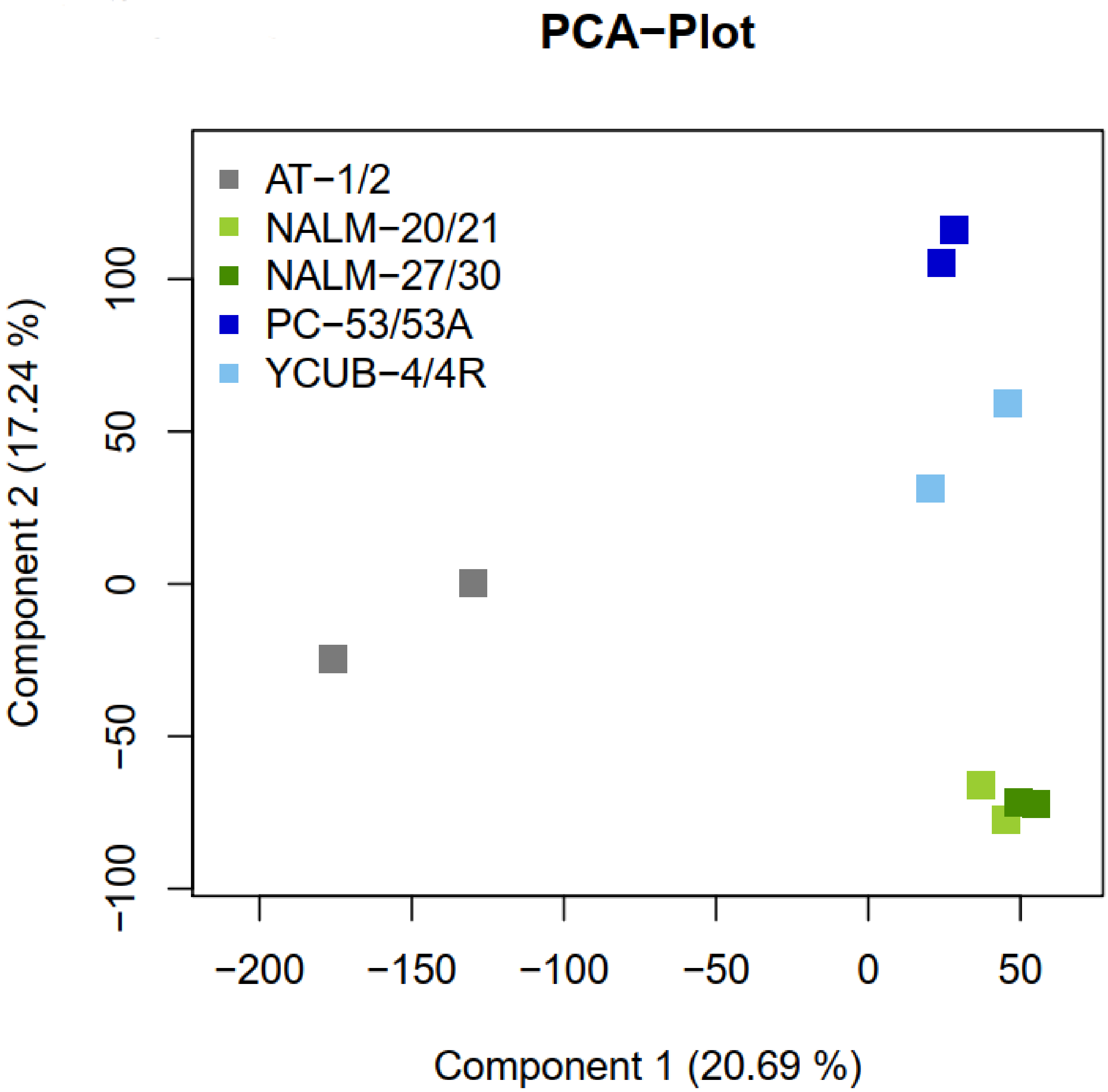

Figure S2. Principal component analysis plot. PCA plot shows that sister cell lines show related expression profiles, in most cases distinguishable from those of unrelated pre-B ALL cell lines.

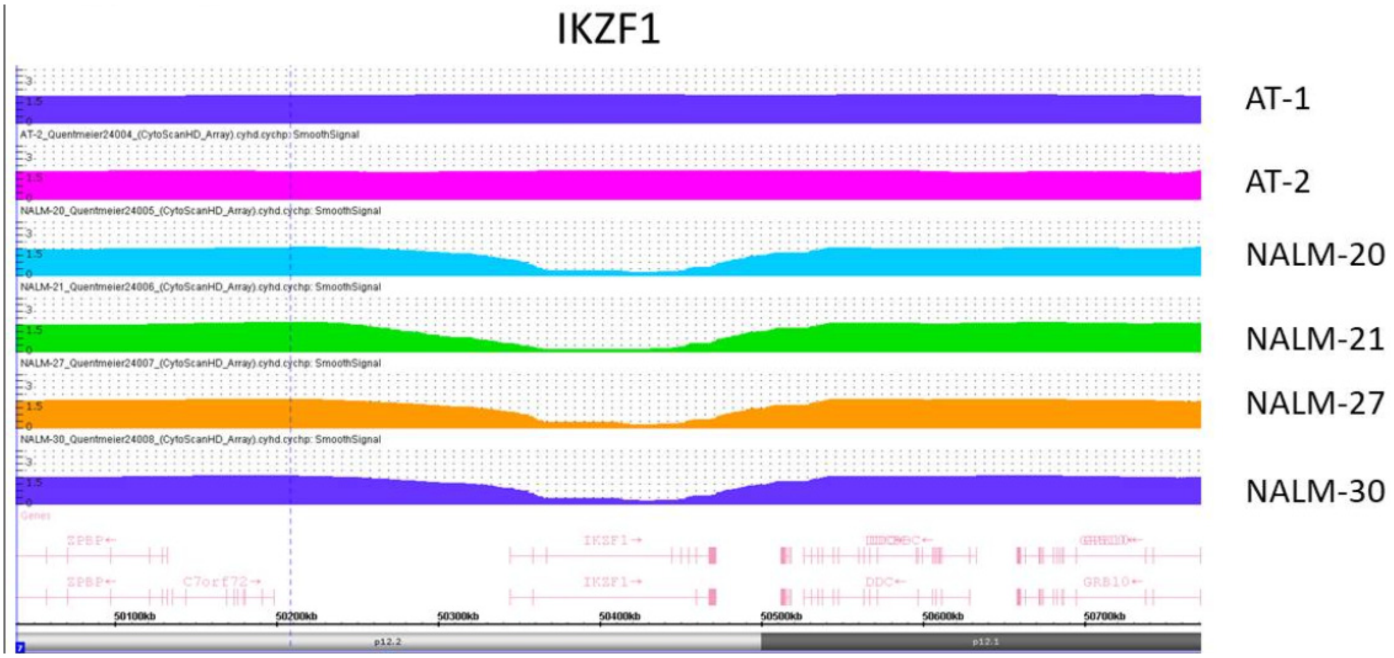

Figure S3. ***IKZF1* deletions.** CGH analysis detects partial deletions (0n) of *IKZF-1* in NALM-20, NALM-21, and NALM-27, NALM-30.

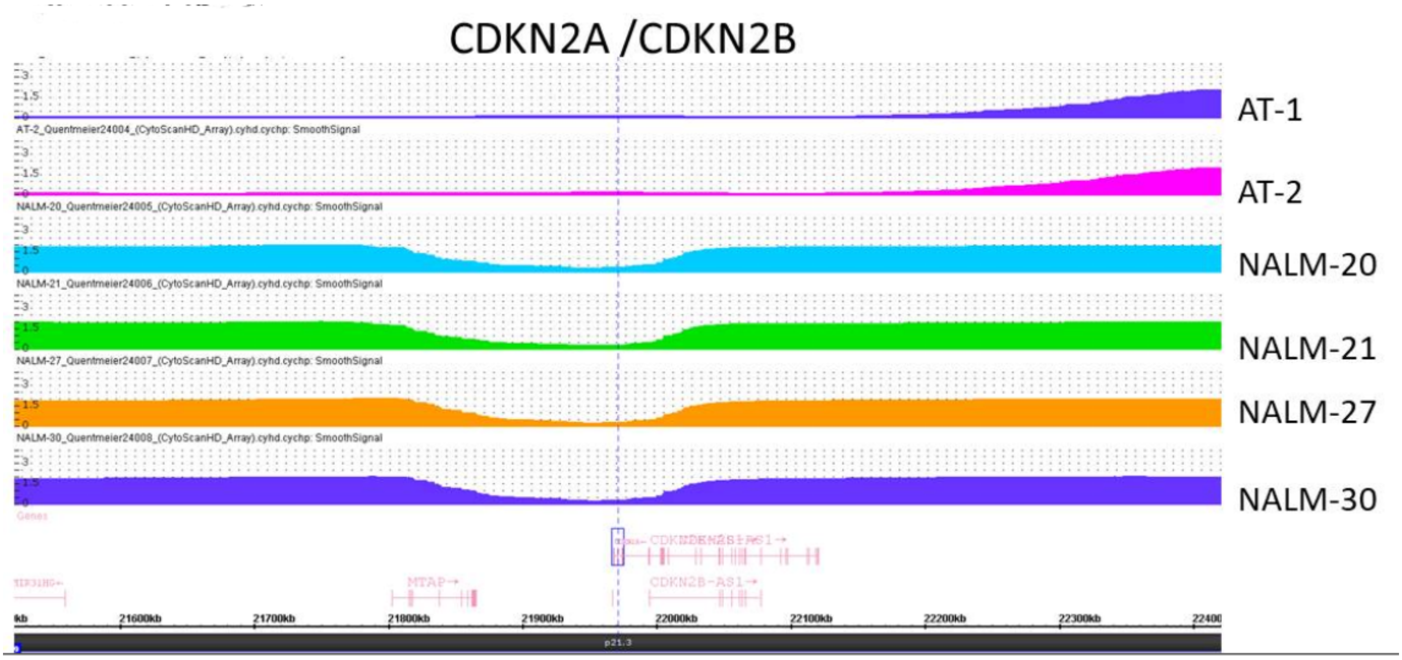

Figure S4. ***CDKN2A* / *CDKN2B* deletions.** CGH analysis detects deletions (0n) of *CDKN2A* and *CDKN2B* in all five pairs of sister cell lines. Shown are exemplary results.

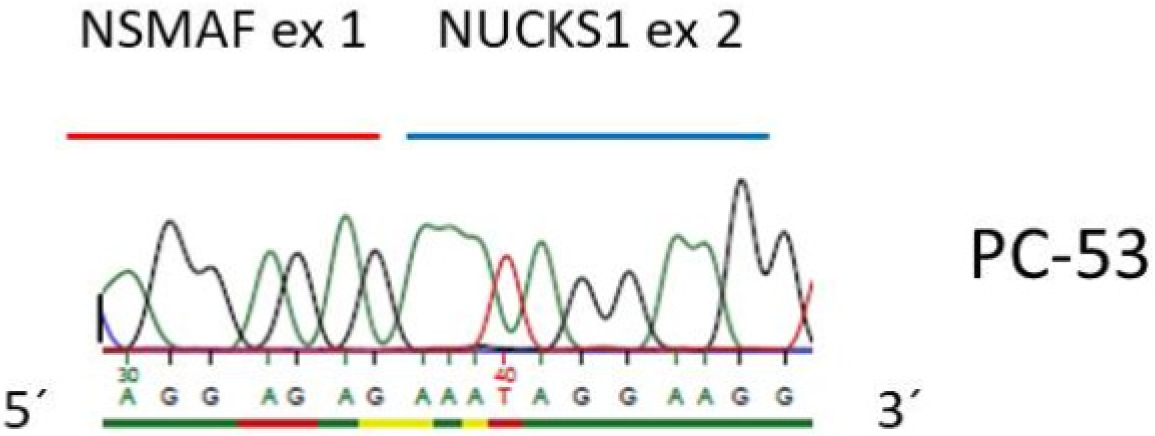

Figure S5. ***NSMAF / NUCKS1* fusion mRNA.** Pre-B ALL cell line PC-53 expresses the in frame *NSMAF* exon 1 / *NUCKS1* exon 2 fusion transcript. The MEF2D/BCL9 PCR product was sequenced with the *MSMAF* exon 1 forward primer (Suppl. Table 3).

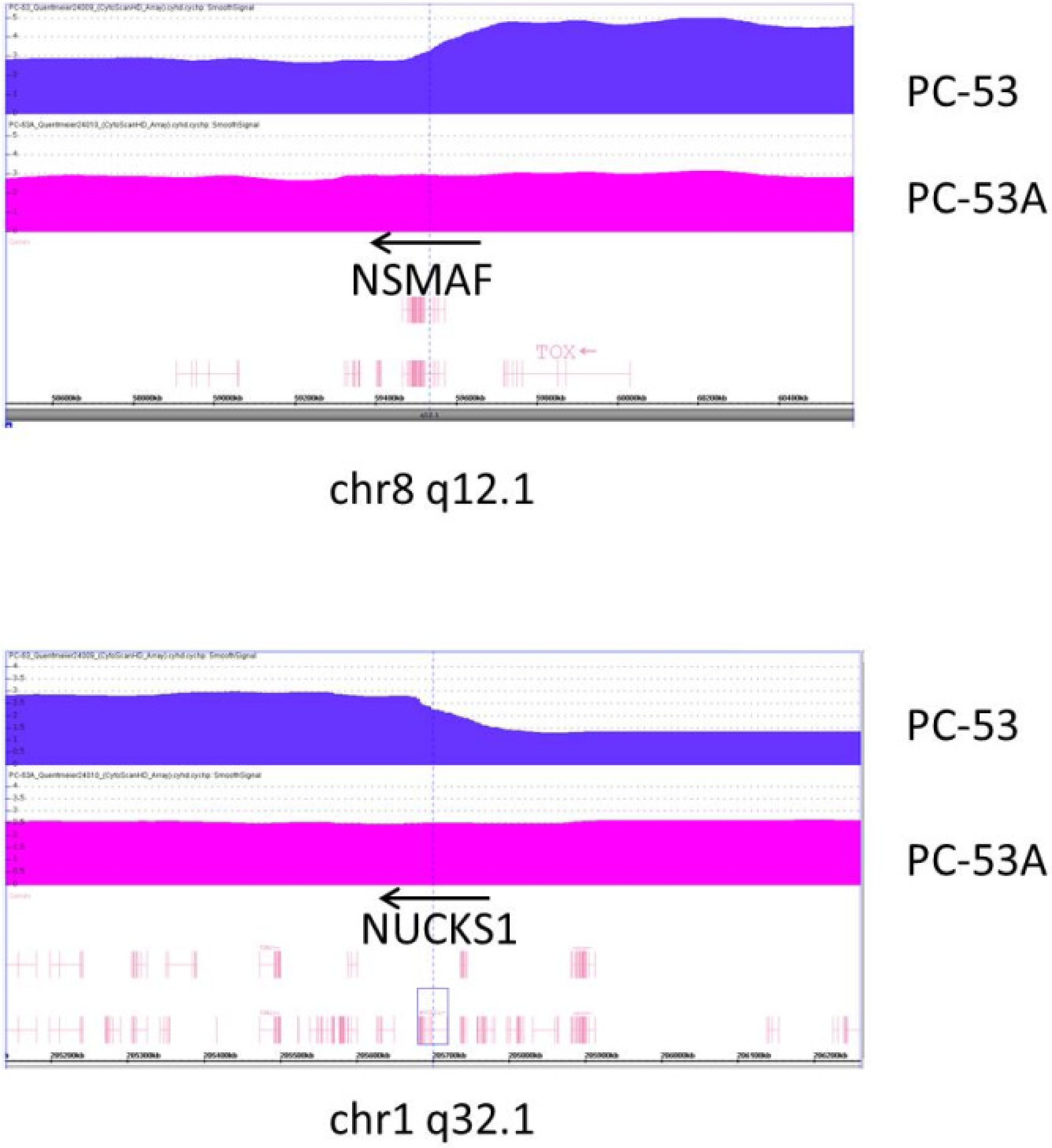

Figure S6. Chromosomal aberrations affecting *NSMAF* and *NUCKS1*. According to CgH analysis, the *NSMAF* / *NUCKS1* positive pre-B ALL cell line PC-53 shows copy number aberrations in the regions of *NSMAF* on chr 8 (q12.1) and of *NUCKS1* on chr 1 (q32.1). The *NSMAF* / *NUCK1* negative sister cell line PC-53A does is not affected by these abnormalities.

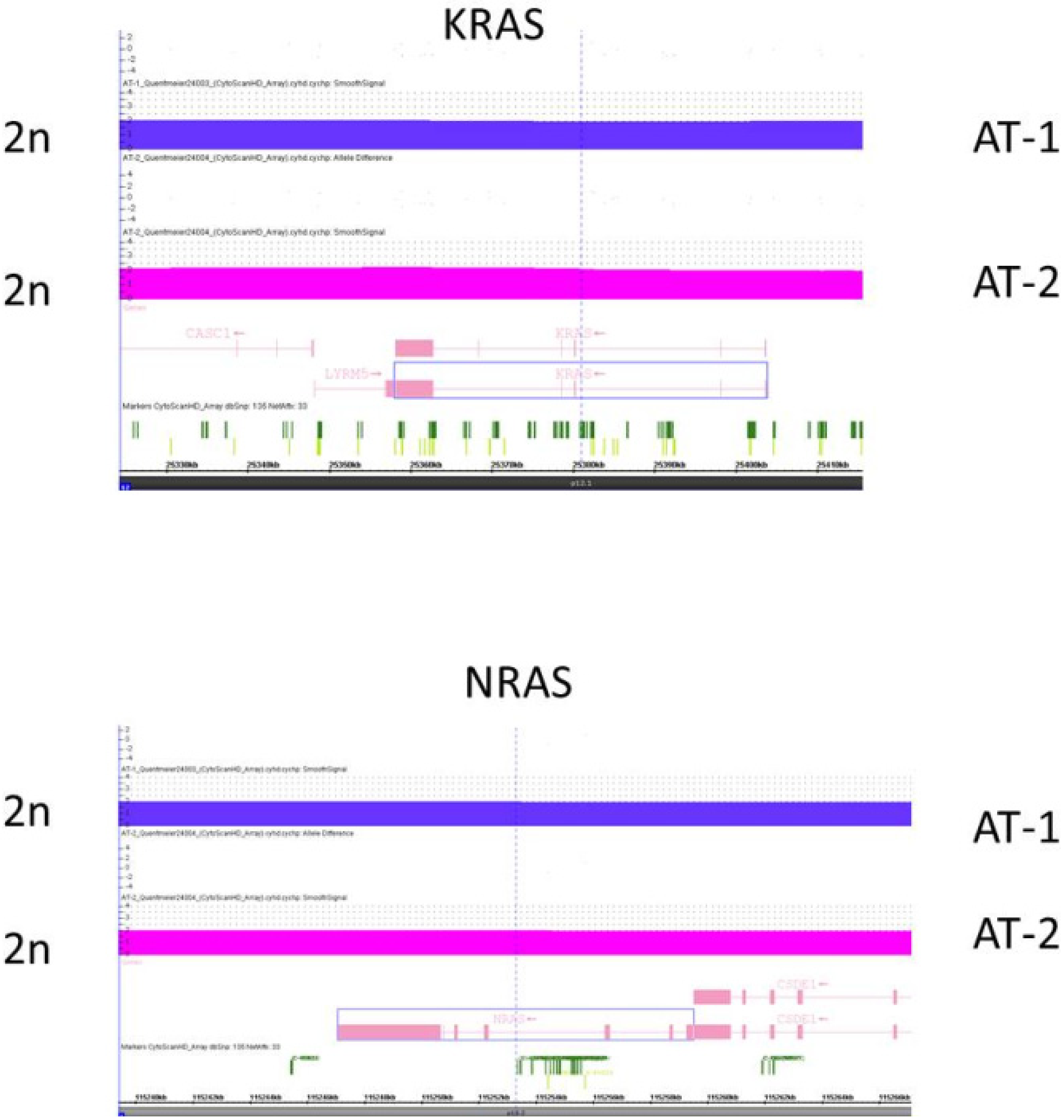

Figure S7. *KRAS* and *NRAS* diploid in AT-1 and AT-2. CGH analysis shows that the pre-B ALL sister cell lines AT-1 and AT-2 are 2n for *KRAS* and *NRAS*.

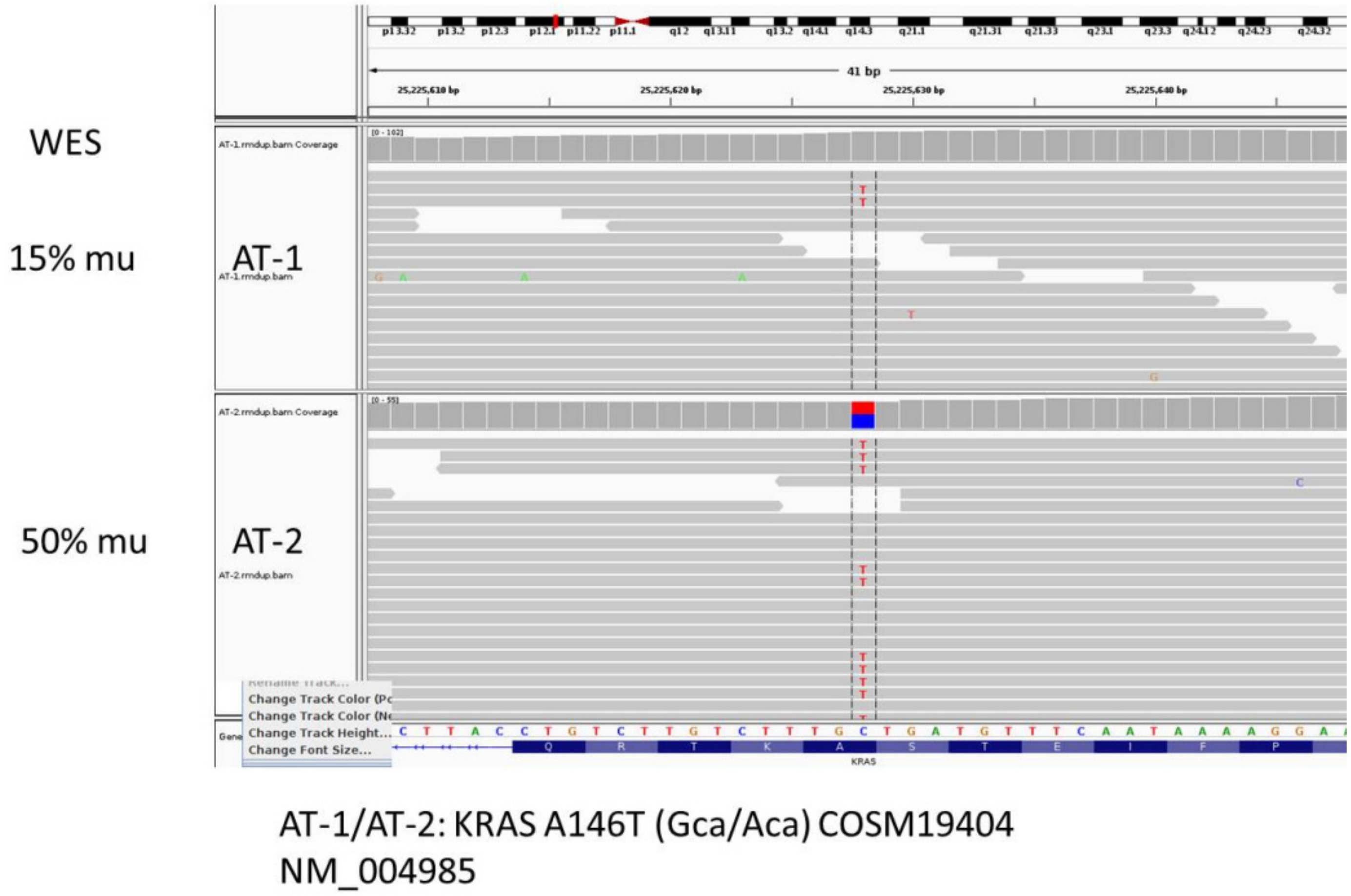

Figure S8. ***KRAS*** **A146T (Gca/Aca) mutation in AT-1 and AT-2.** WES analysis reveals *KRAS* A146T (Gca/Aca) (COSM19494) mutation in pre-B ALL cell lines AT-1 and AT-2. In AT-1, 15% of reads are mutant, in AT-1, 50% are mutant. Sequencing data are visualised by IGV.

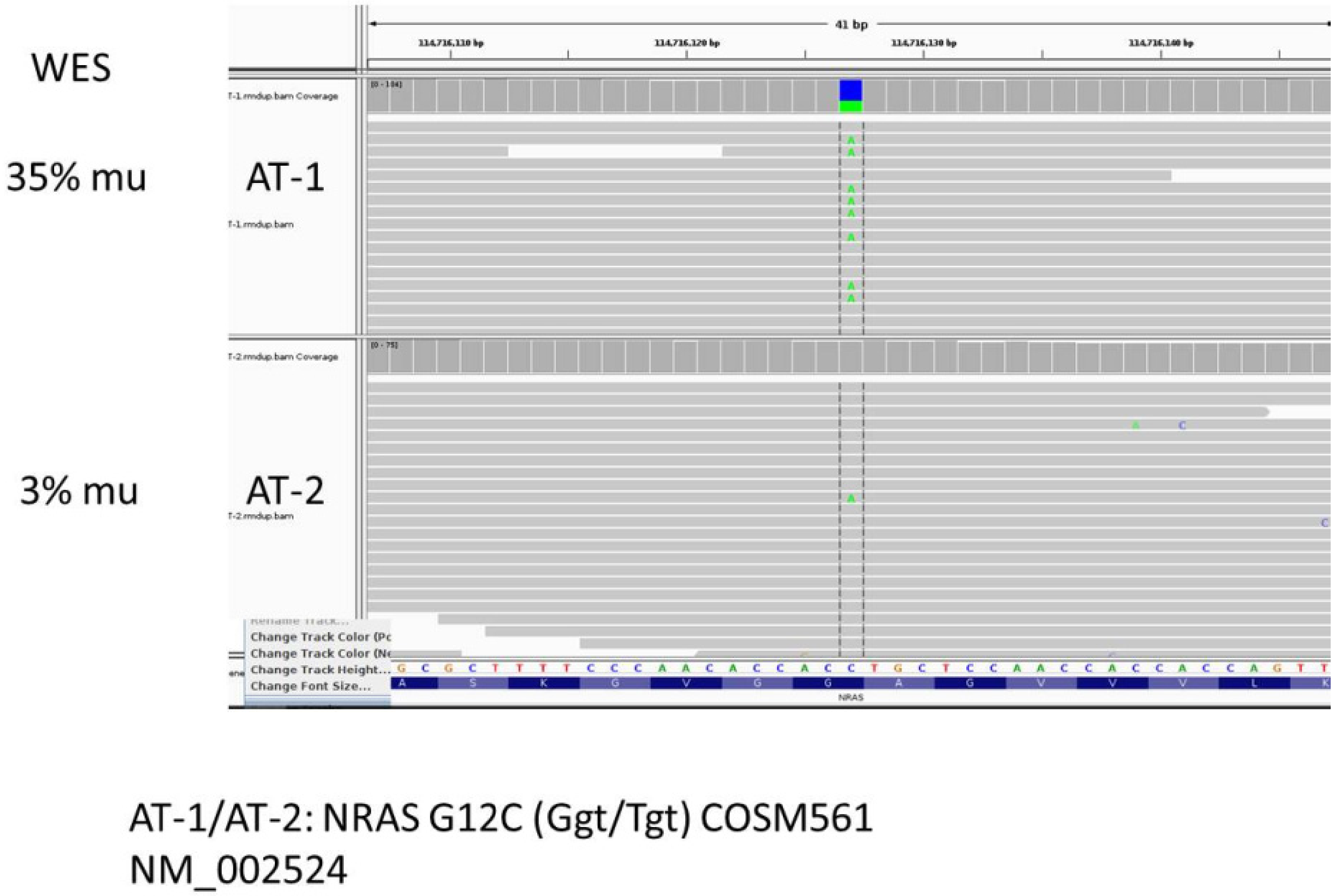

Figure S9. ***NRAS*** **G12C (Gct/Tgt) mutation in AT-1 and AT-2.** WES analysis reveals *NRAS* G12C (Ggt/Tgt) (COSM561) mutation in pre-B ALL cell lines AT-1 and AT-2. In AT-1, 35% of reads are mutant, in AT-2, 3% are mutant. Sequencing data are visualised by IGV.
